# Silent Infection, Early Warning: Animals as Candidate Sentinels for Crimean–Congo Haemorrhagic Fever Surveillance—A Systematic Review

**DOI:** 10.3390/ani16203221

**Published:** 2026-10-13

**Authors:** Wei Yin Vinnie-Siow, Tiong Kai Tan, Norhidayu Sahimin, Sing-Sin Sam, Boon Teong Teoh, Vunjia Tiong, Van Lun Low

**Affiliations:** 1 Higher Institution Centre of Excellence, Tropical Infectious Diseases Research and Education Centre (TIDREC), Universiti Malaya, Kuala Lumpur 50603, Malaysia; vinnie_siow@um.edu.my (W.Y.V.-S.); ayusahimin@um.edu.my (N.S.); singsin@um.edu.my (S.-S.S.); boonteong@um.edu.my (B.T.T.); evationg@um.edu.my (V.T.); 2 Department of Parasitology, Faculty of Medicine, Universiti Malaya, Kuala Lumpur 50603, Malaysia

**Keywords:** Crimean–Congo haemorrhagic fever, CCHFV, *Hyalomma*, livestock, synanthropic animals, zoonosis, One Health, tick-borne disease, seroprevalence, surveillance

## Abstract

Crimean–Congo haemorrhagic fever virus (CCHFV) is a widely distributed tick-borne arbovirus maintained in an enzootic cycle involving wild and domestic mammals and ixodid ticks, particularly *Hyalomma*. This systematic review synthesised 390 eligible publication-level reports published from 2015 through 31 August 2026, comprising 351 independent quantitative reports and 39 linked/nonadditive publications. The evidence tables contained 150 animal, 106 tick and 180 human records across 69 countries. Serology dominated animal investigations; among reports with species-specific raw counts, antibody positivity was highest in camels (72.9%; 3918/5377 across 23 reports), followed by red deer (41.4%) and cattle (25.1%). Among 106 tick reports, CCHFV-positive *Hyalomma* and *Rhipicephalus* were reported in 80 (75.5%) and 41 (38.7%), respectively. Human sensitivity analyses yielded median estimates of 3.8% for antibody positivity, 13.6% for direct/composite viral detection and 20.0% for case fatality. These summaries are descriptive rather than pooled prevalence estimates. Overall, the evidence supports livestock and tick surveillance as complementary components of One Health surveillance, while prospective temporal studies are needed to establish predictive early-warning performance.

## Simple Summary

Crimean–Congo haemorrhagic fever is a tick-borne disease that can cause severe illness in people. The virus circulates silently in many domestic and wild animals, while ticks transmit it between animals and can infect people. We systematically reviewed 390 eligible publication-level reports published from 2015 through 31 August 2026, representing 351 independent quantitative reports. The evidence tables contained 150 animal, 106 tick and 180 human records. Animal studies mainly detected antibodies, whereas direct viral detection was less common. Camels had the largest antibody-positive proportion among commonly studied species, and *Hyalomma* was the most frequently reported CCHFV-positive tick genus. Human sensitivity analyses yielded median estimates of 3.8% for antibody positivity, 13.6% for direct/composite viral detection and 20.0% for case fatality. These findings support integrated animal, tick and human surveillance within a One Health approach, while prospective studies are needed to determine whether animal or vector signals predict subsequent human disease.

## 1. Introduction

Crimean–Congo haemorrhagic fever virus (CCHFV; family *Nairoviridae*, genus *Orthonairovirus*) is a tri-segmented, negative-sense RNA virus that causes Crimean–Congo haemorrhagic fever (CCHF) [1,2]. The World Health Organization (WHO) lists CCHF as a priority disease for research and development in the context of public health emergencies [3]. CCHFV also has the widest documented geographical distribution among tick-borne viruses causing haemorrhagic fever. It circulates across Africa, the Balkans, the Middle East, and Central and South Asia [4], broadly corresponding to the distribution of *Hyalomma* ticks. This broad multi-region distribution has also been documented in a previous global systematic review and meta-analysis [5].

CCHFV is maintained through a tick–vertebrate–tick enzootic cycle [1]. A wide range of wild and domestic vertebrates develop a short period of viraemia, during which feeding ticks can acquire the virus [6]. Although infected animals generally remain asymptomatic, they develop detectable antibody responses, allowing livestock and synanthropic animals to serve as practical indicators of local transmission [6,7]. Seroprevalence reflects previous exposure within a population and may reveal viral circulation before human cases are recognised [7]. Animal surveillance may therefore contribute to earlier indication of local transmission, but indicator value should not be equated with prospectively validated prediction of subsequent human cases.

*Hyalomma* ticks are the principal biological vectors and reservoirs of CCHFV. Transstadial and transovarial transmission allow the virus to persist through tick developmental stages and across generations [8]. The documented geographical range of CCHFV has expanded [2,4,9], with several factors potentially contributing to this pattern. Climate and land-use changes can affect the habitat suitability and seasonal activity of *Hyalomma* ticks, migratory birds can disperse infected immature ticks [10], and livestock movement and trade can transport ticks and viraemic animals between areas. The detection of infected ticks, followed by autochthonous human cases in Spain [10,11], demonstrates the importance of vector surveillance beyond historically recognised endemic areas.

Humans acquire CCHFV infection mainly through tick bites or contact with the blood or tissues of viraemic animals [6]. Although human cases are relatively uncommon, CCHF can cause severe disease [2,3], with case-fatality proportions of 30–40% reported in some clinical series [12]. No vaccine is widely licensed, although an inactivated vaccine is used nationally in Bulgaria [3], and evidence supporting the effectiveness of ribavirin remains limited and contested [13]. The risk of infection is greatest among people with direct exposure to livestock or ticks, including herders, farmers, abattoir workers, and veterinary personnel. Healthcare workers may also be exposed while caring for infected patients, particularly before appropriate infection-prevention and control measures are implemented [12].

The objectives of this systematic review were to synthesise studies published from 2015 through 31 August 2026 reporting CCHFV infection or exposure in animals, ticks and humans; compare the animal species, tick vectors and geographical regions represented in the evidence; evaluate the potential role of animal and vector surveillance as components of early-warning systems; and identify priorities for future One Health surveillance and control.

## 2. Materials and Methods

### 2.1. Search Strategy and Eligibility Criteria

This review used systematic methods for study identification, eligibility assessment, data verification and descriptive synthesis. PRISMA 2020 was used as the reporting framework; no prospective review protocol was registered. PubMed/MEDLINE, Web of Science Core Collection and Scopus were searched through 8 September 2026 for English-language, peer-reviewed original studies published from 2015 through 31 August 2026. Search concepts combined CCHF/CCHFV terminology with animal, tick, human, diagnostic, prevalence and surveillance terms, supplemented by selective citation chasing. Because the exact strings from the earliest search iterations were not archived verbatim, Supplementary Data S1 provides transparent database-specific reconstructions of the documented search concepts and syntax.

Studies were eligible when they reported a primary laboratory-tested CCHFV surveillance or clinical evidence stream in animals, ticks or humans; identified the sampling location and study population or species; and provided usable laboratory results. Reviews, editorials, commentaries, modelling-only studies without distinct primary laboratory data, single-patient reports without usable group-level data, non-English full articles and studies lacking sufficient full-text information for verification were excluded. Eligibility did not depend on whether CCHFV was detected.

### 2.2. Study Selection, Duplicate Handling and Verification

All retrieved records were de-duplicated before screening. Two reviewers independently screened titles/abstracts and assessed full texts, resolving disagreements by discussion. Overlapping publications were reconciled at study-family level: nonadditive reports sharing participants, specimens or case data were linked to a quantitative parent, whereas clearly distinct cohorts, evidence domains or sampling streams were retained separately. Extracted data were verified by the same two reviewers before synthesis.

### 2.3. Data Extraction and Standardisation

For each study, we recorded author, publication year, country or region, population/species, sample size, diagnostic method and positive/tested results for each relevant endpoint. Sampling periods were standardised for human cohorts when explicitly reported; publication year was not used as a surrogate for field-sampling period. Animal antibody and virus-detection findings were recorded separately, with IgG, IgM and multi-marker assays retained as reported by the source.

When a study tested the same animals more than once, they were counted once in overall summaries; confirmation tests or smaller subsets were retained descriptively but not treated as additional samples. Tick taxon summaries included only taxa with CCHFV-positive detections, using current scientific names; AP92, Europe-2 or Aigai-only detections did not contribute to CCHFV-positive taxon summaries.

For human studies, antibody findings and direct evidence of the virus were reported separately. IgM, IgG and other antibody tests were grouped under antibody findings, while RT-PCR, RT-qPCR and similar tests were grouped under virus detection. If a study did not report the number of positive results for each test separately, the combined result was retained in the human evidence table but excluded from summaries of individual test methods.

“NA” indicates that a measure did not apply, while “Not separately reported” indicates that the study did not provide the relevant result on its own.

### 2.4. Data Synthesis and Analysis

Data management, descriptive calculations and reproducibility checks were performed using Python 3.13.5 (Python Software Foundation). Analyses included report/study-family reconciliation, cross-stream counts, descriptive animal summaries, tick-taxon reporting frequencies, human independent-cohort/outcome sensitivity analyses and Wilson 95% confidence intervals. Because study populations, designs, assays and reporting were highly heterogeneous, no pooled meta-analysis or weighting model was applied.

Animal summaries used species-specific positive/tested counts when available; molecular results were summarised only when individual-animal denominators were explicit, otherwise specimen- or pool-level units were retained. Wilson 95% confidence intervals were calculated only for defined tested units. Tick reporting frequency was the number of reports with a positive CCHFV detection for a genus or named taxon divided by all 106 eligible tick reports. Human sensitivity analyses excluded linked/nonadditive publications and retained one explicit positive/tested result per independent cohort and outcome; clearly distinct non-overlapping cohorts could contribute separately. Antibody positivity, direct/composite viral detection and case fatality were analysed separately. Case-fatality calculations used laboratory-confirmed cases or cases with known outcomes as reported by the source.

### 2.5. Study Quality and Potential Bias

Because the evidence spanned heterogeneous observational designs and outcomes, no numerical quality score was assigned. Instead, all 351 independent quantitative reports underwent a structured domain/context assessment covering sampling and selection, representativeness, diagnostic reporting/confirmation, quantitative reporting completeness, temporal reporting and the principal source of selection bias (Supplementary Data S1). Formal funnel-plot/Egger testing and certainty-of-evidence grading were not applied because the review did not estimate a common pooled effect or prevalence.

### 2.6. Generative AI-Assisted Preparation of the Conceptual Framework

The conceptual content of the One Health framework was defined by the authors from the findings of this review and supporting One Health literature. ChatGPT (GPT-6) image-generation functionality (OpenAI, San Francisco, CA, USA; accessed September 2026) was used to assist with the visual rendering of the framework. The resulting graphic was reviewed and edited by the authors for scientific accuracy, consistency with the manuscript and appropriate interpretation. The tool was not used to generate, alter or replace primary study data.

## 3. Results

### 3.1. Study Selection

The database searches identified 4559 records across PubMed/MEDLINE (n = 1239), Scopus (n = 1963) and Web of Science Core Collection (n = 1357). Following the removal of 2422 duplicates, 2137 records underwent title and abstract screening, of which 1430 were excluded. Of the 707 reports progressing to detailed eligibility assessment, 286 were not retained. The remaining 421 reports underwent final source-level assessment, resulting in 31 further exclusions (19 non-English-language publications, eight non-primary reports, three single-patient case reports and one outside the prespecified search capture). Ultimately, 390 eligible publications were retained, comprising 351 independent quantitative reports and 39 linked/non-additive publications (Figure 1). Study-family relationships are documented in Supplementary Data S1.

### 3.2. Overview of Included Studies

The final evidence tables comprised 150 animal, 106 tick and 180 human standardised records. The 351 independent quantitative reports contributed 410 non-mutually exclusive One Health stream contributions (150 animal, 106 tick and 154 human); 43 reports contributed to two streams and eight to all three. The Human_Data table additionally retains 26 linked/nonadditive human publications for transparency. Publication activity increased after 2020 and was highest in 2025 for animal and human evidence, with 27 animal, 10 tick and 30 human records represented that year (Figure 2).

### 3.3. General Overview of Studies by Country and Region

The evidence base comprised 456 study-country mentions across 69 countries (Figure 3, Figure 4 and Figure 5): 153 in Africa, 128 in the Middle East, 91 in Europe, 83 in Asia and one in the Americas. Human evidence was particularly prominent in the Middle East and Asia, whereas animal studies contributed the largest share in Africa and Europe.

At country level, Türkiye had the highest combined number of study-country mentions (45), followed by Iran (37), Pakistan (30), Iraq (28), Spain (27), Senegal (19), Uganda (18) and Kazakhstan (15). These counts describe the distribution of published evidence rather than national disease burden or surveillance intensity.

Several countries contributed animal, tick and human evidence, although these components were often sampled at different sites or times. Each publication was counted once in the overall publication total even when it contributed to multiple evidence domains.

#### 3.3.1. Africa

Africa contributed 153 study-country mentions across 30 countries (65 animal, 31 tick and 57 human). Senegal, Uganda, Nigeria, Cameroon and Kenya contributed the largest numbers, while many countries were represented by only one or two publications. Coverage therefore reflects both surveillance activity and publication availability, and coordinated contemporaneous animal–tick–human sampling remains uncommon.

#### 3.3.2. Middle East

The Middle East contributed 128 study-country mentions across nine countries (32 animal, 34 tick and 62 human). Türkiye (45 mentions) and Iran (37) dominated the regional literature, followed by Iraq (28), making regional coverage heavily influenced by a small number of countries.

Evidence from other Middle Eastern countries was more limited and often restricted to one or two domains. Integrated studies sampling livestock, ticks and exposed human populations at the same place and time would strengthen transmission inference.

#### 3.3.3. Europe

Europe contributed 91 study-country mentions across 19 countries (45 animal, 27 tick and 19 human). Spain had the largest European evidence base, followed by Portugal and several Balkan and Central European countries.

European evidence included both established endemic foci and surveillance in areas of emergence concern. Coverage remained uneven, and several animal studies involved wildlife or zoological collections, requiring ecological context when compared with livestock-based studies.

#### 3.3.4. Asia

Asia contributed 83 study-country mentions across 10 countries (23 animal, 16 tick and 44 human). Pakistan contributed the largest Asian evidence base, followed by Kazakhstan, India and Afghanistan. Human investigations predominated in Pakistan and Afghanistan, while Kazakhstan contributed substantial tick and animal evidence and India contributed both animal and human data.

China and Mongolia were represented mainly by tick and animal studies, whereas Malaysia, Myanmar, Bhutan and Viet Nam contributed relatively few reports, highlighting geographical and thematic gaps across Asia.

### 3.4. CCHFV in Animals

Serology was the predominant approach across the 150 animal reports. Of these, 119 were serology-only, 24 combined serology with molecular testing, five were molecular-only, one combined serology with viral antigen/molecular confirmation and one used antigen detection. Thus, the animal literature primarily describes previous exposure, whereas direct evidence of contemporaneous viraemia or viral detection was less frequently investigated.

Among reports providing species-specific raw counts, camels had the largest antibody-positive proportion (72.9%; 3918/5377 animals across 23 reports), followed by red deer (41.4%; 1124/2716 across six), cattle (25.1%; 11,708/46,612 across 79), buffalo (24.5%; 287/1171 across seven) and wild boar (18.3%; 1729/9456 across eight). Lower proportions were observed in goats (15.1%), sheep (15.0%), donkeys (11.8%), horses (8.9%), dogs (8.7%) and rabbits/hares (0.7%). These are arithmetic summaries of sampled animals in the included literature (Figure 6; Table 1).

Species-specific molecular data were much sparser than serology. Among reports with explicit individual-animal denominators, molecular positivity was 0.7% in cattle (13/1887 across seven reports), 4.0% in camels (48/1199 across six), 0.4% in sheep (2/535 across four) and 0.9% in goats (1/112 across four). Pool-level, mixed-species and specimen-level datasets were not combined with these individual-animal estimates.

#### 3.4.1. Domestic Animals

Domestic livestock dominated the animal evidence base. Species-specific raw data were available most often for cattle, sheep, goats and camels, with additional evidence from buffaloes, horses, donkeys, dogs and other peridomestic animals.

##### Cattle

Cattle provided the largest species-specific evidence base. Across 79 reports with calculable raw serological counts, 11,708/46,612 cattle were antibody-positive (25.1%), with report-level estimates ranging from 0.0% to 98.2%. Seven reports provided explicit species-specific numbers of individual cattle tested, yielding 13/1887 positive cattle (0.7%). The broad serological range indicates marked local heterogeneity rather than a uniform level of exposure.

##### Goats

Goats were represented by 55 publications with species-specific raw serological counts. Overall, 2792/18,543 goats were seropositive (15.1%), with publication-level estimates ranging from 0.0% to 89.2%. Four publications provided explicit species-specific numbers of individual goats tested, with 1/112 goats positive (0.9%).

##### Sheep

Sheep were represented by 61 reports with calculable species-specific serology, comprising 3115/20,739 antibody-positive animals (15.0%). Report-level estimates ranged from 0.0% to 83.3%. Four reports provided explicit species-specific numbers of individual sheep tested and identified 2/535 positive sheep (0.4%).

##### Camels

Across 23 publications with species-specific raw counts, 3918/5377 camels were seropositive (72.9%), with publication-level estimates ranging from 5.3% to 99.6% across studies from Africa, the Middle East and Asia.

Calculable species-specific individual-animal molecular data were available from six camel publications and yielded 48/1199 positive animals (4.0%), with individual study estimates ranging from 0% to 30%. This contrast between frequent antibody detection and less frequent RNA detection is consistent with short-lived viraemia followed by persistent antibody responses and emphasises the importance of sampling timing when interpreting camel surveillance.

##### Buffalo

Buffaloes were represented by seven reports with species-specific raw counts, with 287/1171 animals seropositive (24.5%). Estimates ranged from 0.0% to 75.4%, reflecting substantial geographical heterogeneity across the evidence base.

Some publications combined cattle and buffalo results or did not provide species-specific positive counts; these were not forced into species-level summaries. The table therefore preserves source-reported strata and avoids reconstructing pooled results when the underlying species counts were not available.

##### Horses

Horses were represented by five publications with species-specific raw counts, with 278/3121 seropositive (8.9%). Publication-level estimates ranged from 2.4% to 70.3%, with higher values reported in some African settings than in European surveys.

Horses were less frequently studied than major livestock species, and the available evidence was geographically limited.

##### Dogs

Dogs were represented by four publications with species-specific raw counts, with 63/721 seropositive (8.7%) and estimates ranging from 0.0% to 56.2%.

##### Donkeys

Donkeys were represented by four publications with species-specific raw counts, with 100/848 seropositive (11.8%) and publication-level estimates ranging from 5.8% to 31.4%. As with horses, the number of studies remains small relative to the evidence available for cattle, sheep, goats and camels.

#### 3.4.2. Wildlife

Wildlife evidence was more heterogeneous than livestock evidence and included cervids, wild boar, carnivores, lagomorphs and other mammals. Much of the larger-sample wildlife literature originated from the Iberian Peninsula and France, while studies from Africa and other regions often involved smaller numbers or mixed wildlife assemblages.

##### Red Deer

Red deer were represented by six publications with species-specific raw counts. Across these publications, 1124/2716 animals were seropositive (41.4%), with estimates ranging from 7.9% to 76.1%.

##### Wild Boar

Wild boar were represented by eight publications with species-specific raw counts, with 1729/9456 seropositive (18.3%) and estimates ranging from 1.4% to 40.6%.

##### Other Species

Other wildlife and companion-animal taxa were generally represented by one or two publications, including roe deer, mouflon, foxes, cats, pigs, large felids and Iberian lynx. Because the evidence for these taxa was sparse or geographically restricted, results are presented at study level in Table 2 and were not used for broad species-level inference.

### 3.5. CCHFV in Ticks

A total of 106 eligible tick reports were included. Ninety-seven contained at least one CCHFV-positive tick detection and nine yielded no CCHFV detection in the tested tick material. Molecular testing was used in 103/106 reports; three reports used antigen detection without molecular testing. Hyalomma was explicitly reported as CCHFV-positive in 80/106 eligible tick reports (75.5%). At species level, *H. marginatum* was the most frequently reported named taxon (27/106; 25.5%), followed by *H. asiaticum* (16/106; 15.1%), *H. anatolicum* (15/106; 14.2%), *H. rufipes* (13/106; 12.3%) and *H. dromedarii* (12/106; 11.3%). Genus-level reporting frequencies are summarised in Figure 7.

Thirty-seven named tick taxa were reported as CCHFV-positive in the included studies, of which 28 appeared in at least two publications (Table 3; Figure 8). Nine named taxa were reported once. Genus-only positive identifications were retained in Table 4 but were excluded from named-species frequency counts. Historical *Boophilus decoloratus* records were harmonised as *Rhipicephalus decoloratus*, and *Rhipicephalus sanguineus* sensu lato was harmonised as the *R. sanguineus* complex.

#### 3.5.1. *Hyalomma*

*Hyalomma* was explicitly reported as CCHFV-positive in 80/106 eligible tick reports (75.5%). At species level, *H. marginatum* was the most frequently reported named taxon (27/106; 25.5%), followed by *H. asiaticum* (16/106; 15.1%), *H. anatolicum* (15/106; 14.2%), *H. rufipes* (13/106; 12.3%) and *H. dromedarii* (12/106; 11.3%).

Other repeatedly reported *Hyalomma* taxa included *H. scupense* (7 publications), *H. impeltatum* (6), *H. truncatum* (6), *H. excavatum* (5), *H. lusitanicum* (5) and *H. detritum* (3). Less frequently reported taxa included *H. aegyptium*, *H. impressum*, *H. schulzei* and *H. turanicum*.

*Hyalomma*-positive records spanned Africa, Europe, the Middle East and Asia, with the species detected varying by ecological setting, host community and sampling strategy.

#### 3.5.2. *Rhipicephalus*

*Rhipicephalus* was explicitly reported as CCHFV-positive in 41/106 tick reports (38.7%). The *R. sanguineus* complex appeared in 20/106 (18.9%) and *R. bursa* in 12/106 (11.3%); *R. turanicus* was reported in six reports, *R. annulatus* in five, *R. appendiculatus* in four, and *R. decoloratus* and *R. evertsi evertsi* in three each.

Less frequently reported *Rhipicephalus* taxa included *R. geigyi*, *R. guilhoni*, *R. microplus*, *R. mushamae* and *R. rossicus*.

#### 3.5.3. Other Genera (*Dermacentor*, *Amblyomma*, *Haemaphysalis*, *Boophilus*, *Ixodes*)

Other CCHFV-positive genera were reported less frequently: *Dermacentor* in nine reports, *Amblyomma* in nine, *Haemaphysalis* in eight, *Ixodes* in six and *Argas* in one. *Amblyomma variegatum* was reported in 9/106 reports (8.5%), *Dermacentor marginatus* in seven and *Ixodes ricinus* in six.

Additional taxa included *Haemaphysalis punctata* (five publications), *H. parva* (two), and *Dermacentor nuttalli*, *D. pictus*, *Haemaphysalis sulcata* and *Amblyomma lepidum* (one each).

The 97 reports with at least one CCHFV-positive tick detection are summarised in Table 4; the remaining nine tick surveillance studies are presented in Table 5.

#### 3.5.4. Geographic Distribution of CCHFV-Positive Tick Records

CCHFV-positive tick records were geographically widespread (Figure 9a), with included studies from 37 countries. Iran contributed the largest number of tick publications (17), followed by Türkiye (8), Kazakhstan and Senegal (7 each), Spain (6), and Ghana (4).

For the country–taxon matrix (Figure 9b), only CCHFV-positive ticks identified to named species and assigned to a single study country were included. Based on the corrected Table 4 positive-taxon fields, 36 of the 37 countries contributed at least one named country-specific taxon record. India was represented among CCHFV-positive tick investigations, but its available positive tick result was not attributed to a named species. Genus-only identifications and unattributed positive pools were not assigned to species in the matrix.

Countries with larger and more sustained tick-surveillance programmes generally reported a broader range of taxa. Overall, *Hyalomma* remained the principal surveillance target, while other ixodid genera contributed additional locally relevant detections.

### 3.6. CCHFV in Humans

Sensitivity analysis of human antibody positivity included 118 cohort/outcome estimates from 104 independent publications, with a median of 3.8% (range 0.0–100.0%; Table 6; Figure 10).

Direct/composite viral-detection positivity included 72 cohort/outcome estimates from 71 independent publications, with a median of 13.6% (range 0.0–100.0%). Direct viral testing was concentrated in clinically suspected, febrile and outbreak-associated populations.

Case fatality included 54 cohort/outcome estimates from 54 independent publications, with a median of 20.0% (range 0.0–100.0%). The human evidence records underlying these summaries are presented in Table 7.

## 4. Discussion

One Health recognises the interdependence of human, animal and environmental health and the need for coordinated action across veterinary, entomological, environmental and public health sectors [390]. CCHFV exemplifies this interdependence through asymptomatic infection in many vertebrate hosts [6], viral maintenance and transmission by ticks [8], environmental influences on tick distribution [9], and human infection through tick bites, animal contact and healthcare-associated exposure [2].

Figure 11 presents a conceptual One Health framework integrating the animal, tick and human evidence in this review with established literature on environmental and climate-related drivers. It illustrates how complementary surveillance signals could support earlier risk detection, while recognising that predictive relationships require prospective validation.

### 4.1. Livestock and Synanthropic Animals as Candidate Sentinels for CCHFV Surveillance

Frequent CCHFV antibody detection in livestock supports their use as indicators of viral circulation, but frequent exposure is not equivalent to validated early-warning performance [6,7]. A useful sentinel should provide a temporally interpretable, geographically relevant and actionable signal; key considerations include age, antibody persistence, animal movement, seroconversion, sampling accessibility and linkage to local tick and human surveillance [391].

Age strongly influences livestock serology because older animals have had more opportunities for exposure. Age-associated increases have been reported in Mauritanian livestock [124], Ugandan livestock [87] and camels in the United Arab Emirates [128]. Age-standardised sampling or predefined younger age groups would therefore improve temporal and geographical comparability.

Antibody persistence further limits the temporal interpretation of a single IgG-positive sample, and repeated tick exposure may maintain or boost responses. Maternal anti-CCHFV antibodies have also been demonstrated in sheep [391]. Baseline-seronegative animals are therefore more informative for detecting subsequent seroconversion.

Animal movement can weaken the geographical interpretation of serology because the place of sampling may differ from the place of infection. Studies at a large United Arab Emirates livestock market detected CCHFV RNA-positive camels and associated ticks and demonstrated the importance of recording animal origin and trade history [114,128]. Market, abattoir and movement-checkpoint surveillance should therefore document recent transport and origin wherever possible.

For early warning, prospective monitoring of young or baseline-seronegative animals is more temporally informative than repeated cross-sectional seroprevalence. Fixed cohorts could be resampled across periods of tick activity and interpreted alongside molecular testing of animals and ticks and contemporaneous human surveillance [391].

Spatial linkage and operational timeliness are also important. Ugandan livestock studies demonstrated marked geographical heterogeneity and the value of animal serology for risk mapping [87,392]. Validation of sentinel performance requires repeated animal, tick and human surveillance at common sites and reporting of practical measures such as sampling frequency, laboratory turnaround time and the interval between a signal and response.

Cattle provided the largest species-specific evidence base and may be practical candidates for prospective surveillance where geographically stable herds are routinely accessible to veterinary services. Their usefulness depends on local management, movement and tick exposure rather than the magnitude of cross-sectional seropositivity alone.

Camels had the largest antibody-positive proportion in the included literature (72.9%), whereas molecular detection was much less frequent (4.0%). Studies in the United Arab Emirates have similarly reported high camel antibody positivity with occasional CCHFV RNA detection in camels or associated *Hyalomma* ticks [114,128], and abattoir studies demonstrate opportunities to link camel and occupational surveillance [55]. Markets, transport routes and abattoirs may therefore be informative surveillance nodes, particularly when younger, recently arrived or baseline-seronegative camels are followed over time.

Dogs may provide complementary information on peridomestic exposure because they live close to people and livestock and encounter local ticks. However, only four publications provided species-specific raw serological counts, so their sentinel value remains uncertain and requires prospective validation.

Wildlife surveillance can identify enzootic circulation and wildlife–livestock interfaces [6], but irregular access, animal movement and difficulty with repeated sampling limit its operational use for early warning. Wildlife serology may therefore be most useful for ecological surveillance and geographical risk mapping.

Overall, candidate sentinel species should be validated prospectively using repeated, spatially linked animal–tick–human surveillance and demonstrating that animal signals occur early enough to support intervention.

### 4.2. Tick Vector Ecology and Control Implications

The frequent detection of CCHFV in *Hyalomma* across the included studies is consistent with its established role in viral maintenance and transmission [2,8,393]. Control measures may include acaricide treatment of livestock, management of pastures and animal-holding areas, and inspection or treatment at markets, abattoirs and movement checkpoints, tailored to local tick ecology and livestock practices.

CCHFV was also detected in *Rhipicephalus*, *Dermacentor*, *Amblyomma* and *Haemaphysalis*, but field detection of viral RNA or antigen does not by itself establish vector competence. Positive ticks may contain virus acquired during a recent blood meal; experimental transmission studies are needed to determine which species acquire, maintain and transmit infectious virus [393].

Animal and tick studies also measured different biological signals: most tick studies used RT-PCR to detect viral RNA at collection, whereas animal studies relied mainly on serology reflecting previous exposure. Coordinated sampling of animals and ticks at the same sites and times would make these complementary indicators easier to interpret together.

### 4.3. Human Risk Profile and Clinical Burden

Human evidence spanned community surveys, occupational groups, febrile or suspected patients, outbreak/contact investigations and healthcare workers. Antibody positivity was generally low in community cohorts but higher in some occupationally exposed or selected populations, while clinically confirmed CCHF remained potentially severe [2,12].

Evidence of increased occupational exposure was reported in several geographical settings. In Nigeria, seroprevalence reached 92.3% among herdsmen, compared with 7.1% among febrile hospital patients in the same investigation [285]. Seroprevalence was also reported in 29.1% of abattoir workers in the United Arab Emirates [55]. In Türkiye, the corresponding estimates were 12.5% among animal breeders and 4.0% among abattoir workers, while a study from Cameroon reported 17.8% among herders compared with 8.3% among febrile patients [77,321]. By contrast, seroprevalence among animal handlers in Ghana was 3.7% [219]. These differences indicate that occupational category alone does not determine exposure; local tick abundance, animal infection levels, working practices, and the frequency and duration of animal contact are also likely to be important [394].

Healthcare-associated transmission represents a separate risk pathway. Among 96 healthcare workers investigated following suspected occupational exposure in Iran, 3.13% were seropositive and 11.46% were RT-PCR-positive. Two deaths occurred among the 12 confirmed cases, corresponding to a case fatality of 16.67% [286]. Unlike infections associated with ticks or livestock, healthcare-associated transmission generally results from unprotected contact with the blood or body fluids of an infected patient. Because the early clinical presentation of CCHF is non-specific and resembles that of other febrile illnesses, healthcare workers may be exposed before the disease is suspected. Appropriate infection-prevention and control measures should therefore be available in areas where CCHF is endemic or considered a plausible diagnosis, rather than introduced only after laboratory confirmation [395].

In the independent-cohort sensitivity analysis, the median case fatality was 20.0% (range 0.0–100.0%). The widest estimates generally arose from small cohorts, underscoring the importance of interpreting percentages alongside case counts and confidence intervals. Consistent case definitions and complete outcome reporting would improve comparability across studies.

### 4.4. An Integrated One Health Framework for Surveillance and Control

Integrating animal, tick and human surveillance requires coordination among veterinary, entomological, environmental, and public health authorities. The Quadripartite One Health Joint Plan of Action, developed by the WHO, World Organisation for Animal Health (WOAH), Food and Agriculture Organization (FAO) and United Nations Environment Programme (UNEP), provides an established framework through which CCHFV surveillance and control activities could be coordinated [390]. Three priorities arise from the findings of this review.

First, diagnostic methods should be standardised. The included studies used varied serological platforms and RT-PCR assays, complicating direct comparison; similar concerns have been raised in recent reviews of CCHFV diagnostics [396]. Harmonised, validated protocols and compatible veterinary and public-health laboratory methods would improve cross-sector interpretation and data exchange.

Second, animal, tick and human sampling should be coordinated at common sites and periods. Integrated sentinel sites at livestock holdings, markets, abattoirs or movement routes could combine animal serology, tick testing and surveillance of occupationally exposed groups, supported by compatible databases and joint risk mapping.

Third, geographical gaps should be addressed. Evidence from the Americas was particularly limited, and coverage remained sparse in parts of Southeast and East Asia and uneven in several Balkan and Anatolian countries. Targeted field studies can help distinguish limited surveillance or publication from true absence of documented circulation.

These priorities can build on existing veterinary, public-health, vector-control, diagnostic, wildlife and environmental systems. Shared protocols, compatible data standards and jointly defined surveillance objectives would allow animal and tick findings to contribute more directly to human risk assessment, while predictive early-warning performance should be established prospectively.

### 4.5. Limitations

Several limitations should be considered. The review was restricted to English-language, peer-reviewed studies indexed in PubMed/MEDLINE, Web of Science Core Collection and Scopus and published from 2015 through 31 August 2026, which may introduce language, database and publication bias. The 2015 boundary was a pragmatic contemporary-scope decision, and reconstructed database strategies limit exact reproduction of the earliest searches. Study designs, populations, diagnostics and reporting varied substantially, and the structured context assessment does not substitute for a design-specific certainty framework. Sampling periods were standardised for human cohorts but not systematically across animal and tick datasets, so publication-year trends should not be interpreted as field-sampling trends. Most animal and tick evidence was cross-sectional, limiting temporal inference about whether these signals precede changes in human risk.

## 5. Conclusions

This systematic review synthesised 390 eligible publication-level reports, including 351 independent quantitative reports and 39 linked/nonadditive publications, with 150 animal, 106 tick and 180 human records. The evidence demonstrates heterogeneous animal exposure, frequent CCHFV detection in *Hyalomma* ticks and substantial human exposure and clinical burden across diverse settings. Together, these findings support integrated livestock, tick and human surveillance within a One Health framework. Animals are best regarded as indicators of CCHFV circulation and candidate sentinels rather than validated predictors of subsequent human cases.

Future work should prioritise prospective validation of sentinel performance, standardised diagnostics, coordinated animal–tick–human sampling and improved geographical coverage. Serial sampling of young or baseline-seronegative animals linked to contemporaneous tick and human surveillance could determine whether seroconversion or viraemia provides a useful early signal. Genomic and phylogeographical analyses and climate-informed risk models may further strengthen surveillance, provided their predictive relationships are validated prospectively.

## Figures and Tables

**Figure 1:**
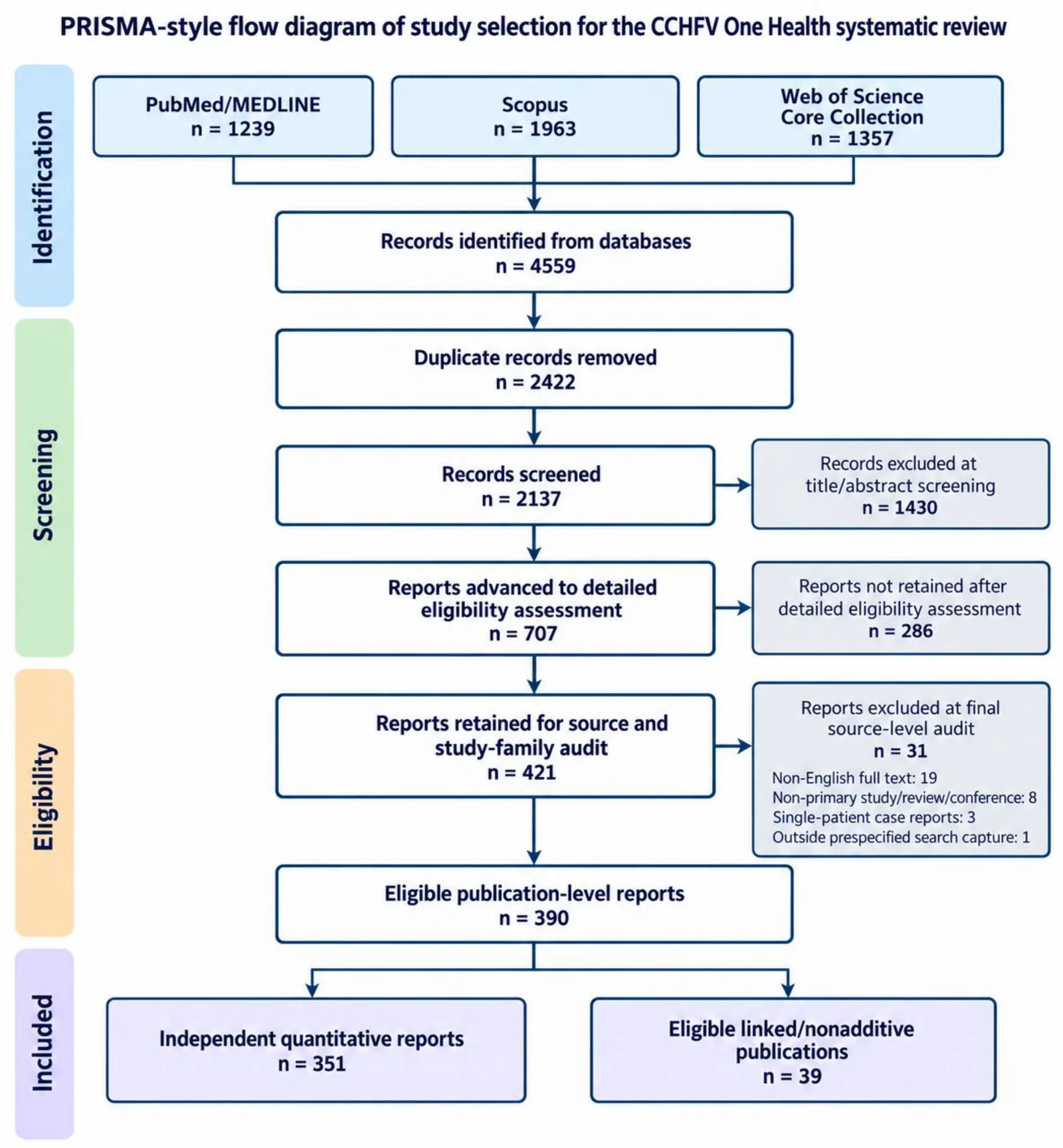
PRISMA-style flow diagram for identification, screening, eligibility assessment, source-level reconciliation and inclusion of eligible publication-level reports.

**Figure 2:**
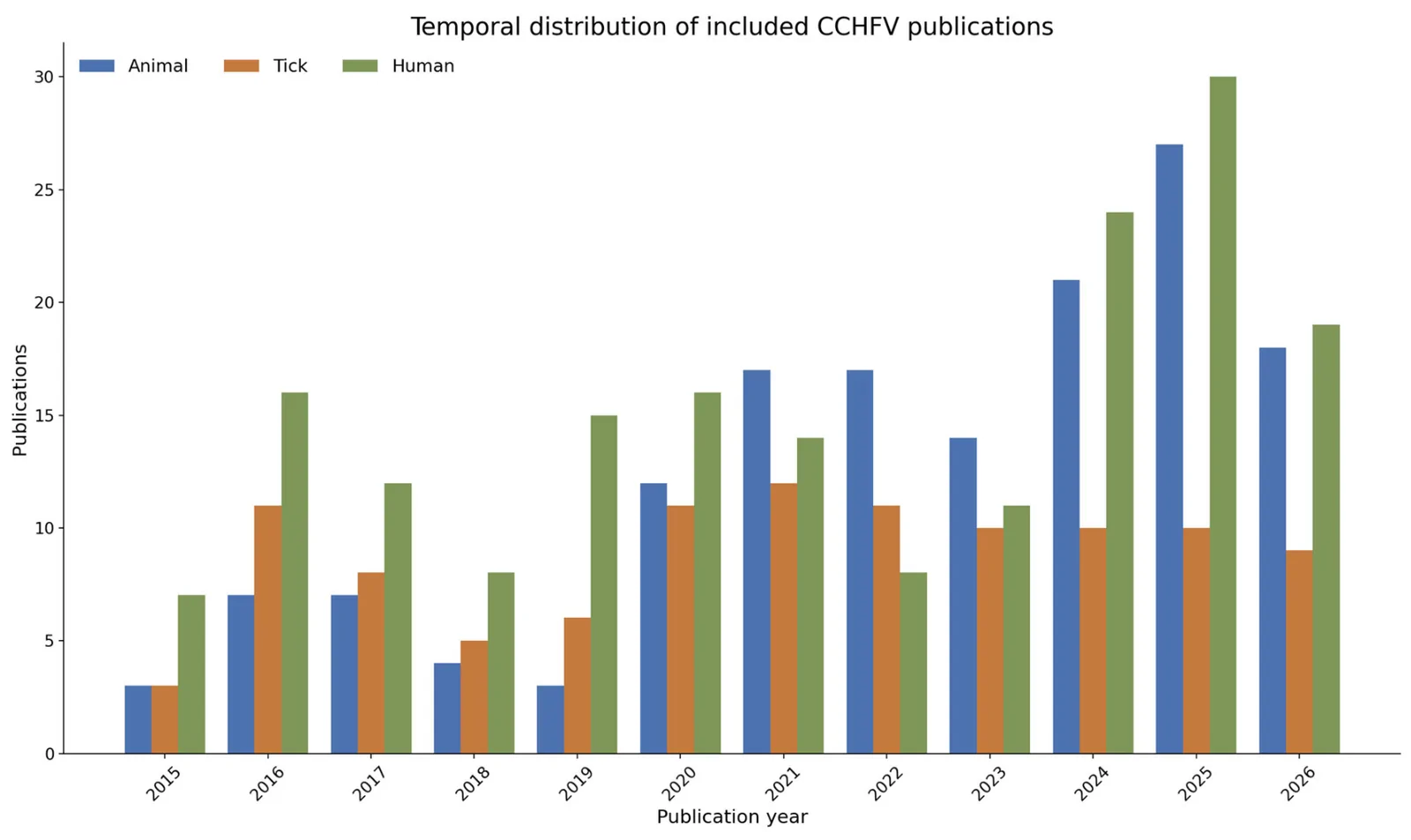
Temporal distribution of included CCHFV evidence by stream (animal n = 150; tick n = 106; human n = 180), 2015–31 August 2026.

**Figure 3:**
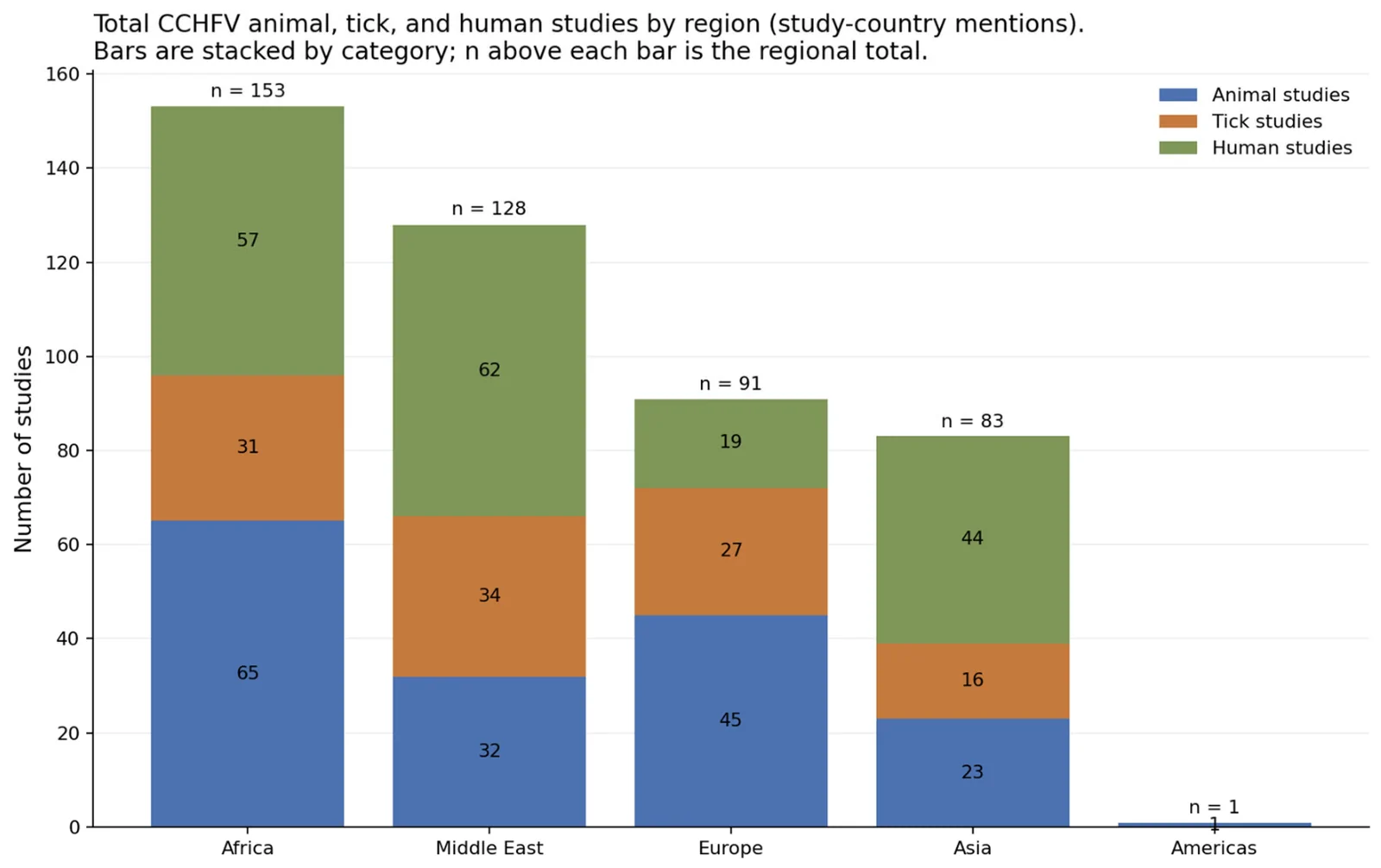
Regional distribution of CCHFV study-country mentions, grouped by animal, tick and human evidence.

**Figure 4:**
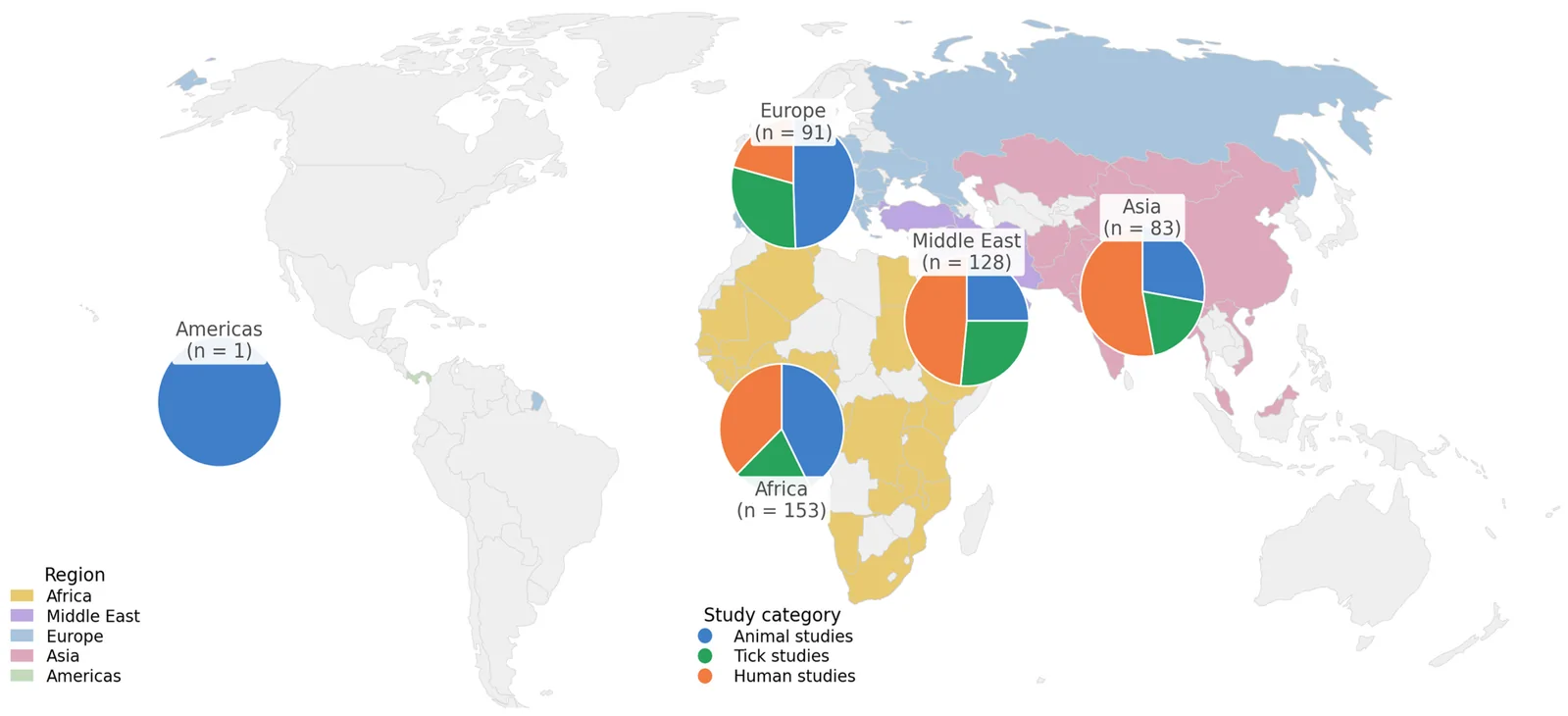
Global overview of CCHFV evidence included in this review. Each regional pie chart shows the relative contribution of animal, tick and human study-country mentions; n indicates the total number of study-country mentions in that region.

**Figure 5:**
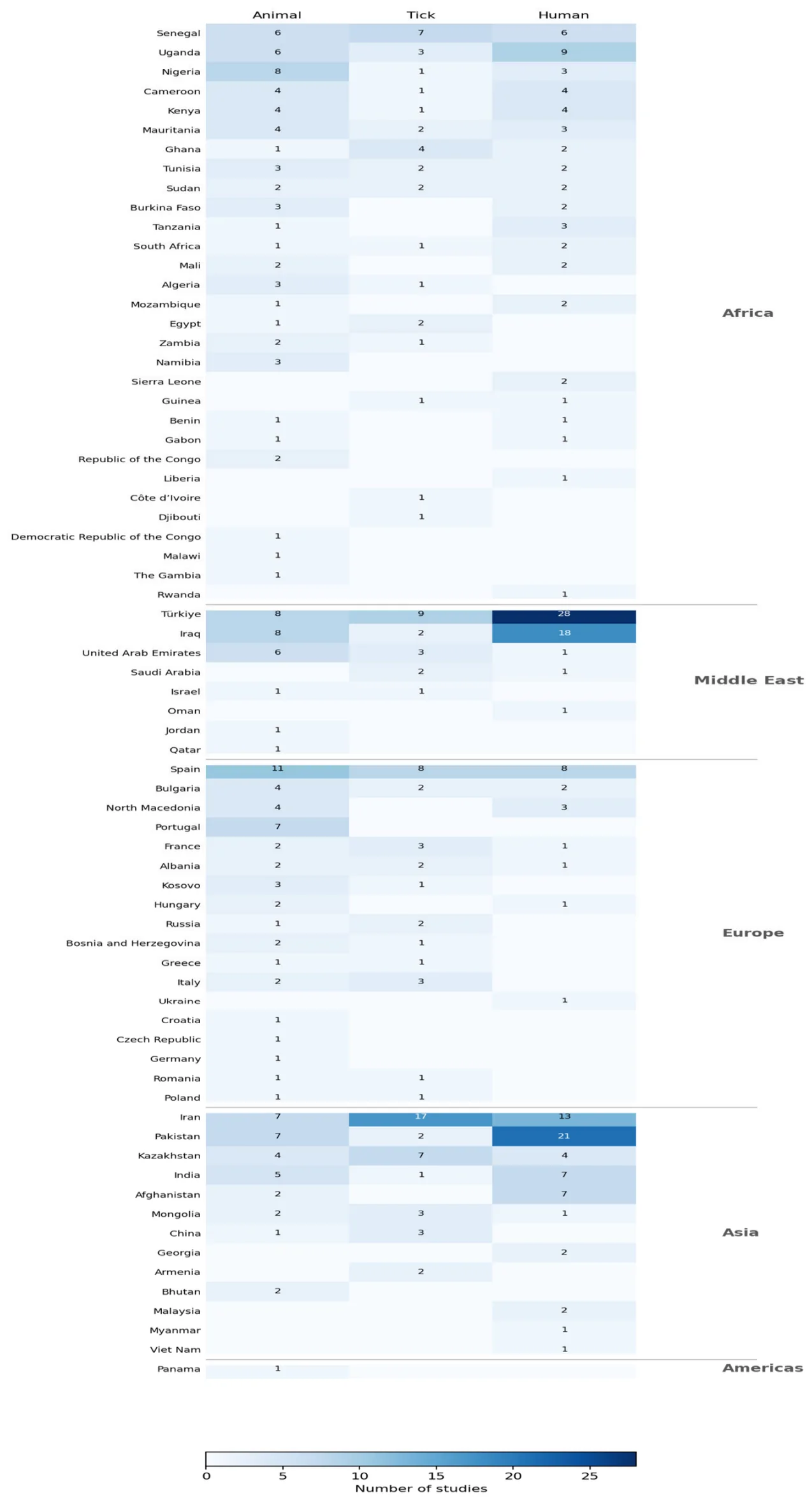
Heatmap of CCHFV study-country mentions by country and evidence type, grouped by region.

**Figure 6:**
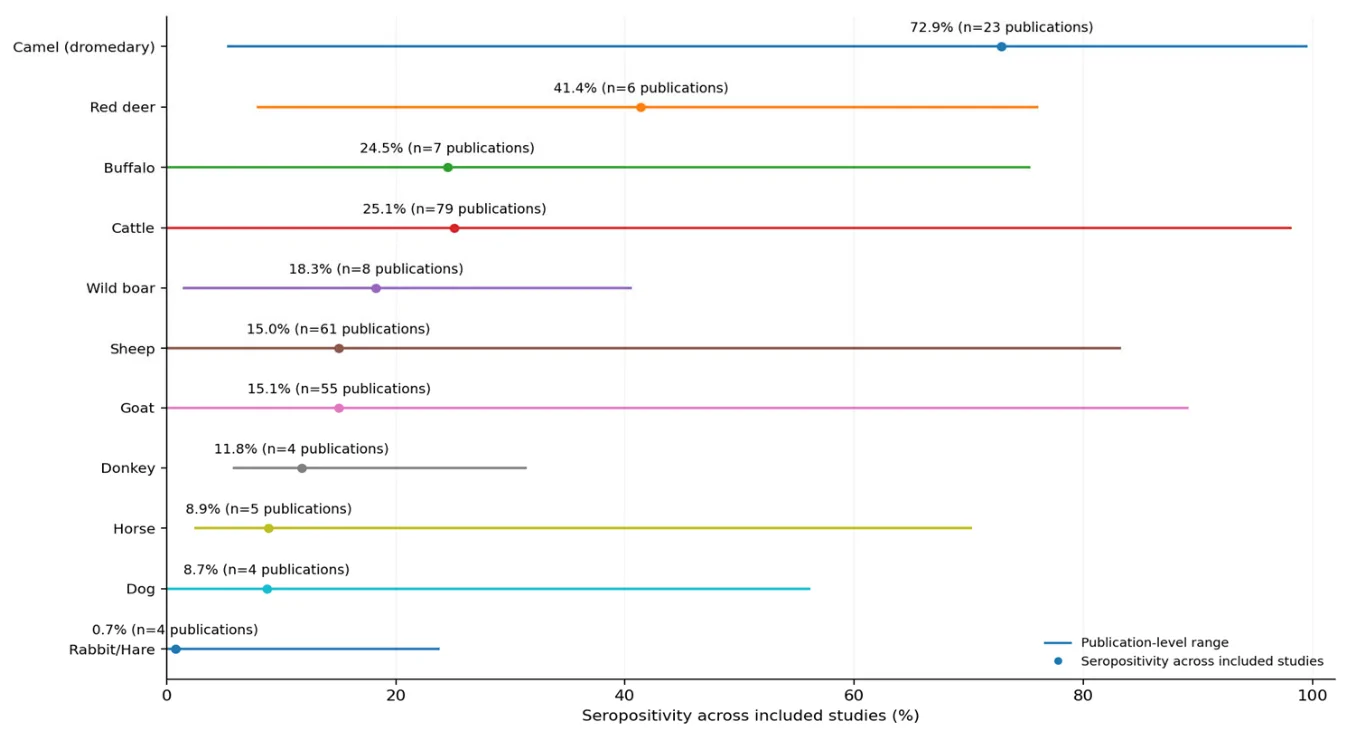
Descriptive anti-CCHFV antibody positivity among sampled animals by species with at least three reports providing species-specific raw counts. Point = Σ positive/Σ tested; line = report-level range; n indicates contributing reports.

**Figure 7:**
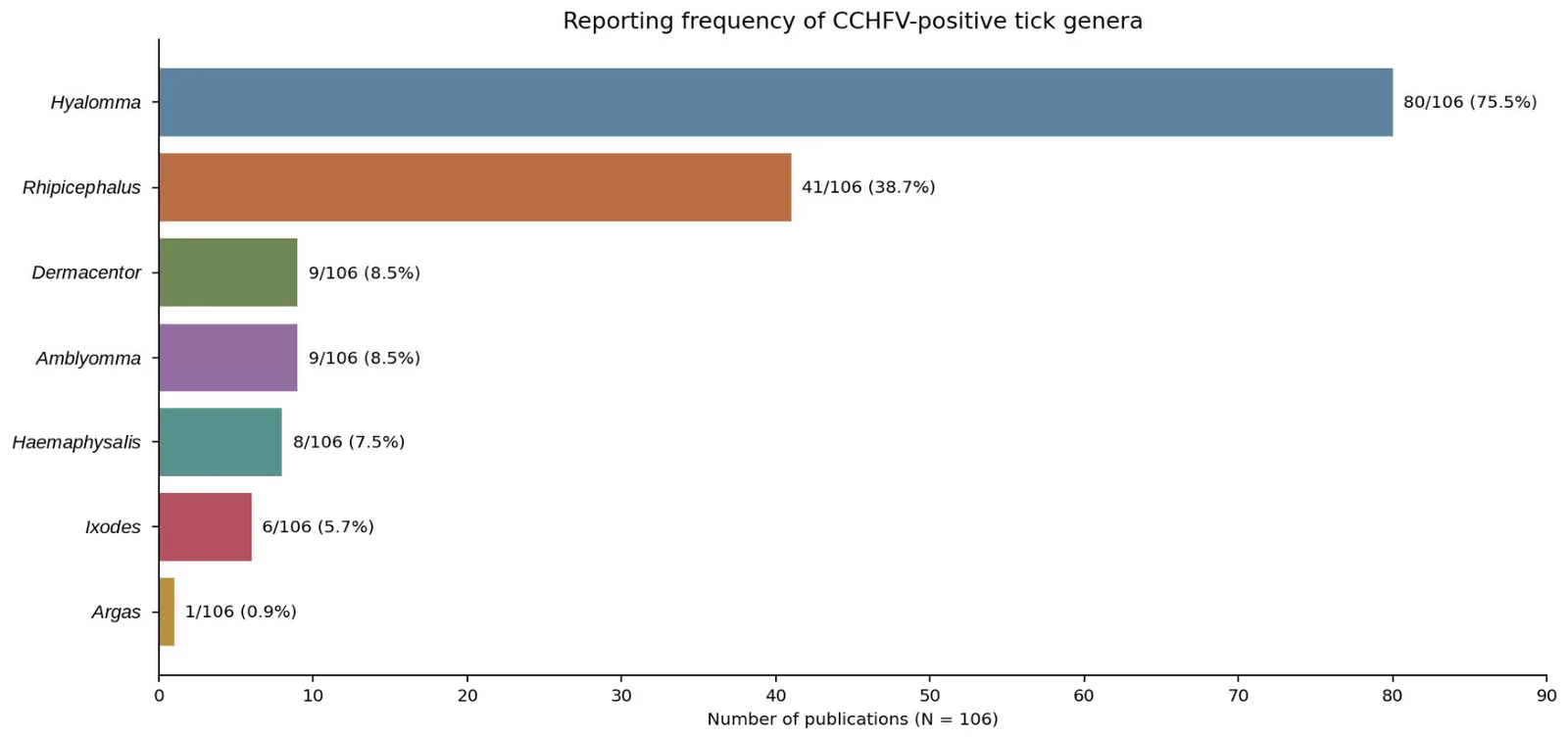
Publication-reporting frequency of CCHFV-positive tick genera across all 106 eligible tick reports. Values are not tick-level positivity estimates.

**Figure 8:**
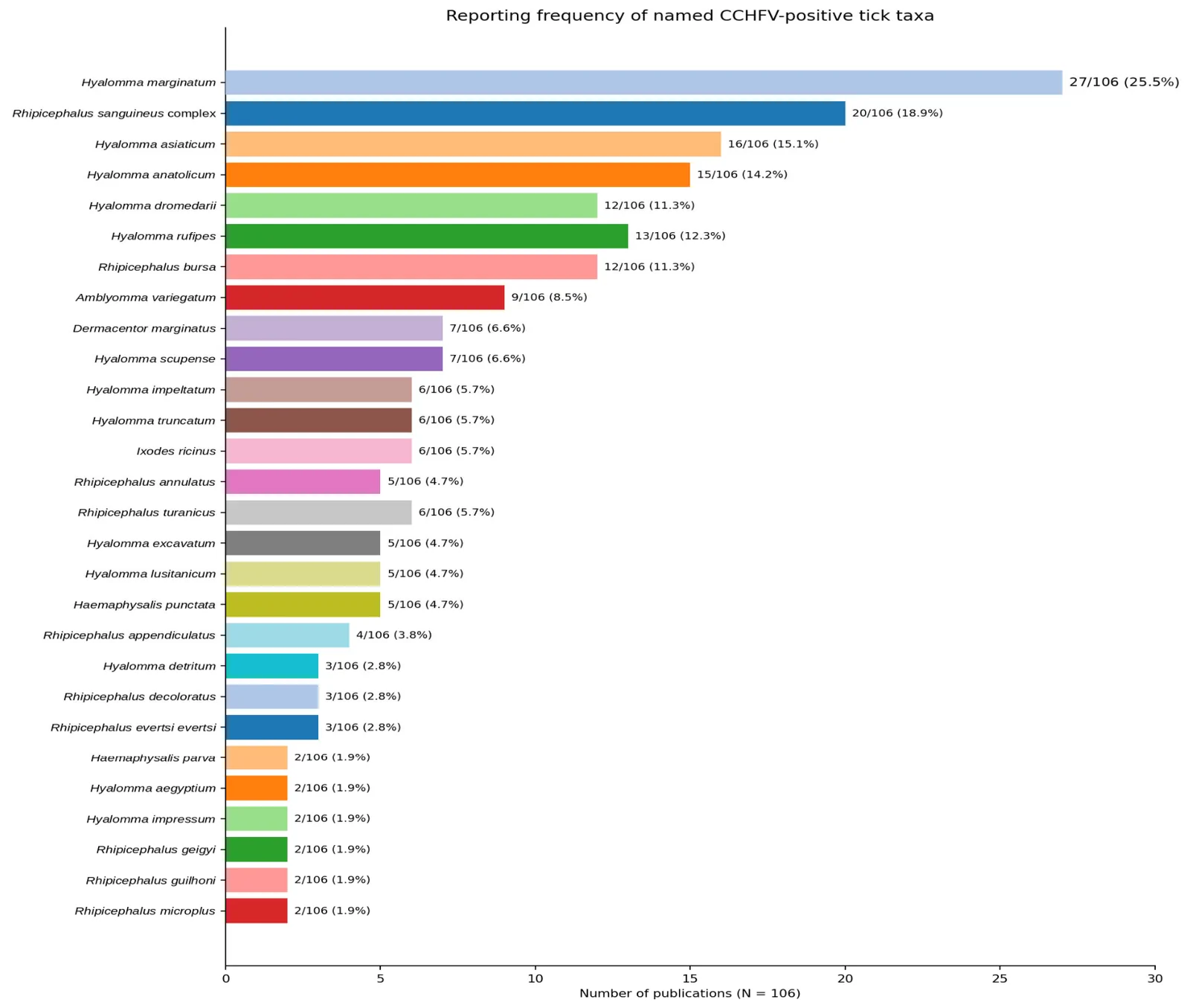
Publication-reporting frequency of named CCHFV-positive tick taxa among 106 eligible tick reports, reconciled against Table 3. Only taxa appearing in at least two reports are shown. These are not tick-level positivity estimates.

**Figure 9:**
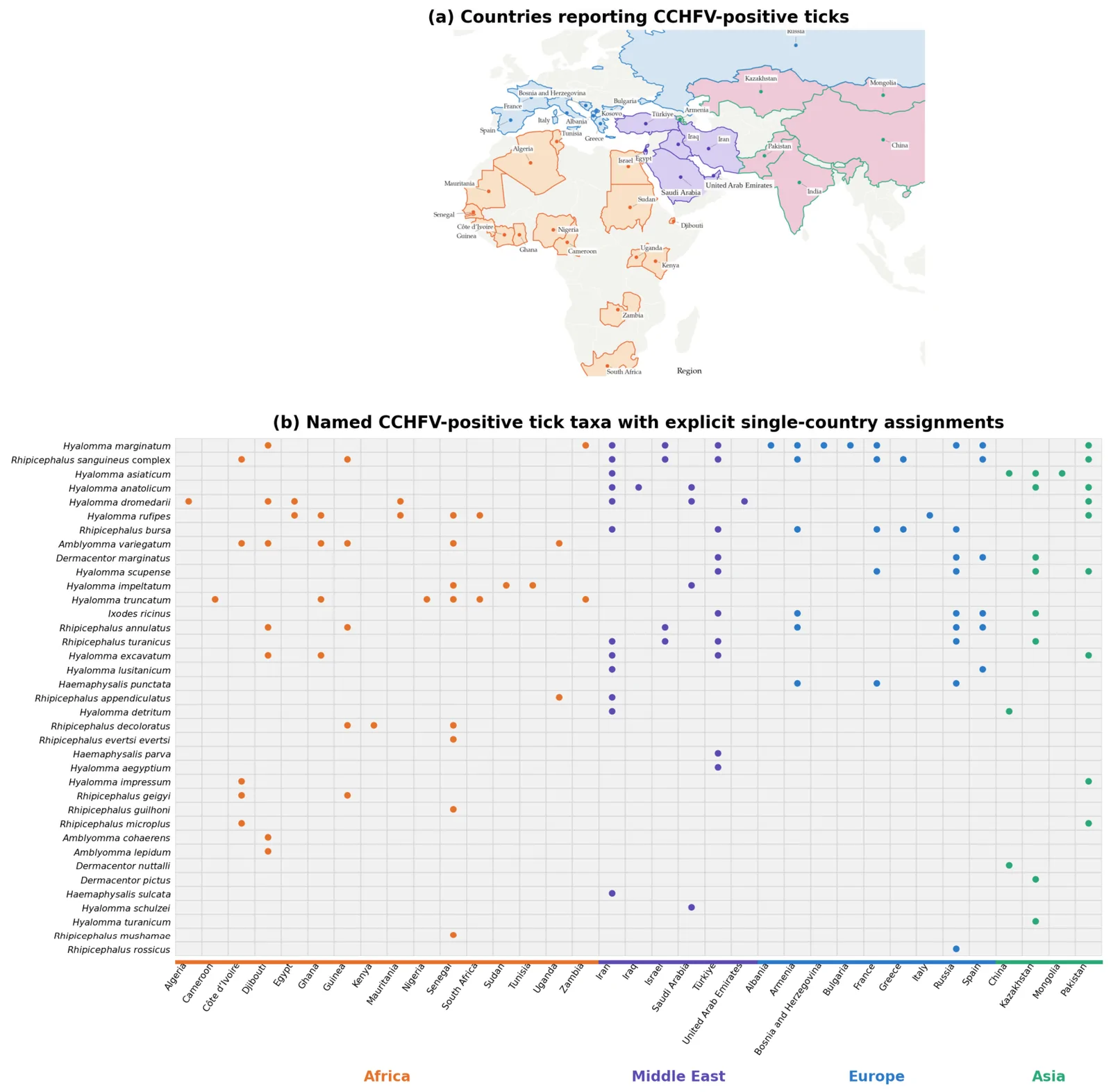
Geographic and taxonomic distribution of CCHFV-positive tick evidence. (**a**) Countries represented among the 97 positive-detection tick reports within the 106 eligible tick reports, shaded by region. (**b**) Selected named CCHFV-positive tick taxa with explicit single-country assignments; dots indicate at least one positive report from that country.

**Figure 10:**
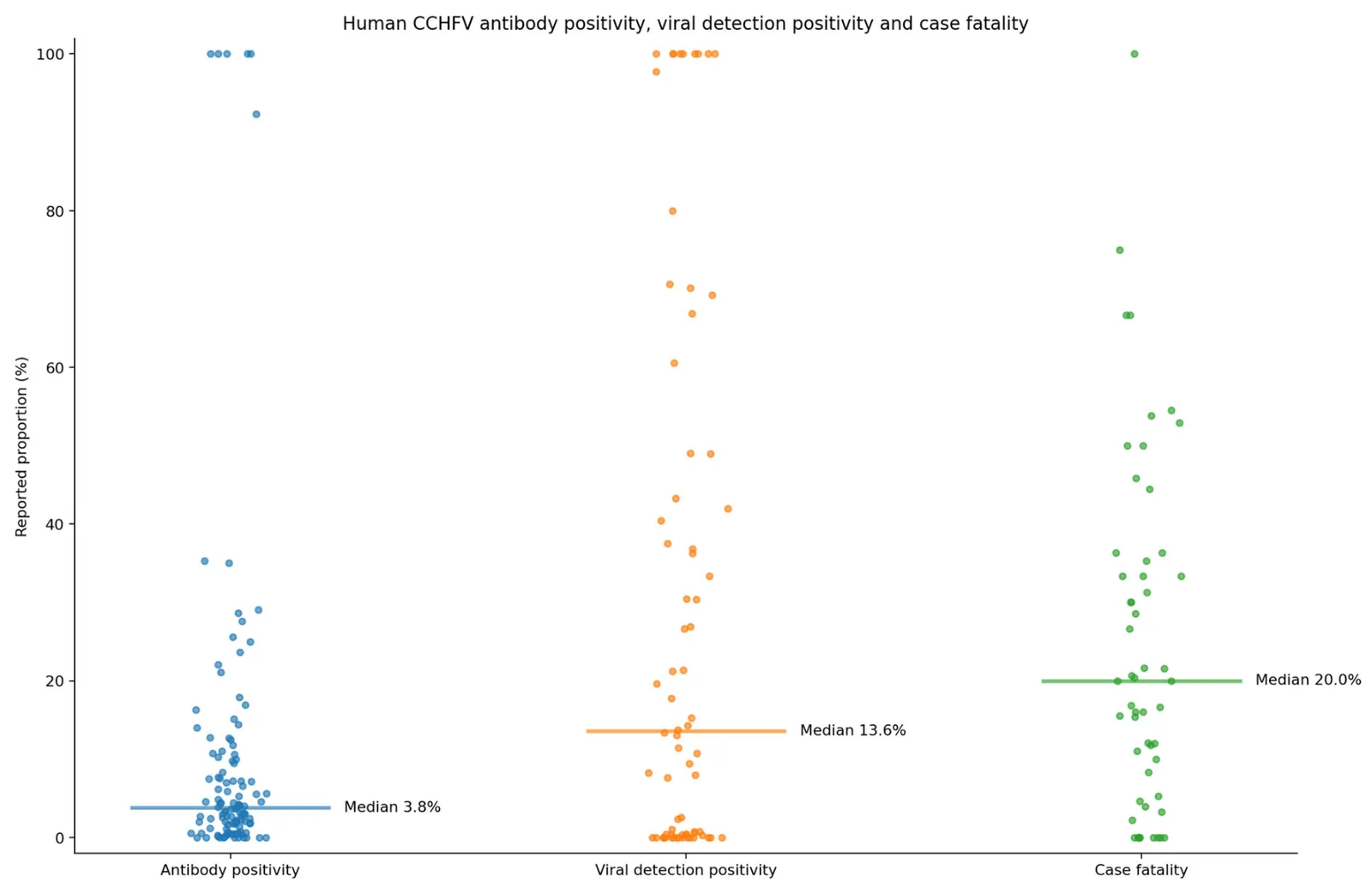
Distribution of human CCHFV antibody positivity, direct/composite viral-detection positivity and case-fatality sensitivity estimates. Points represent one estimate per independent cohort/outcome; horizontal bars indicate medians.

**Figure 11:**
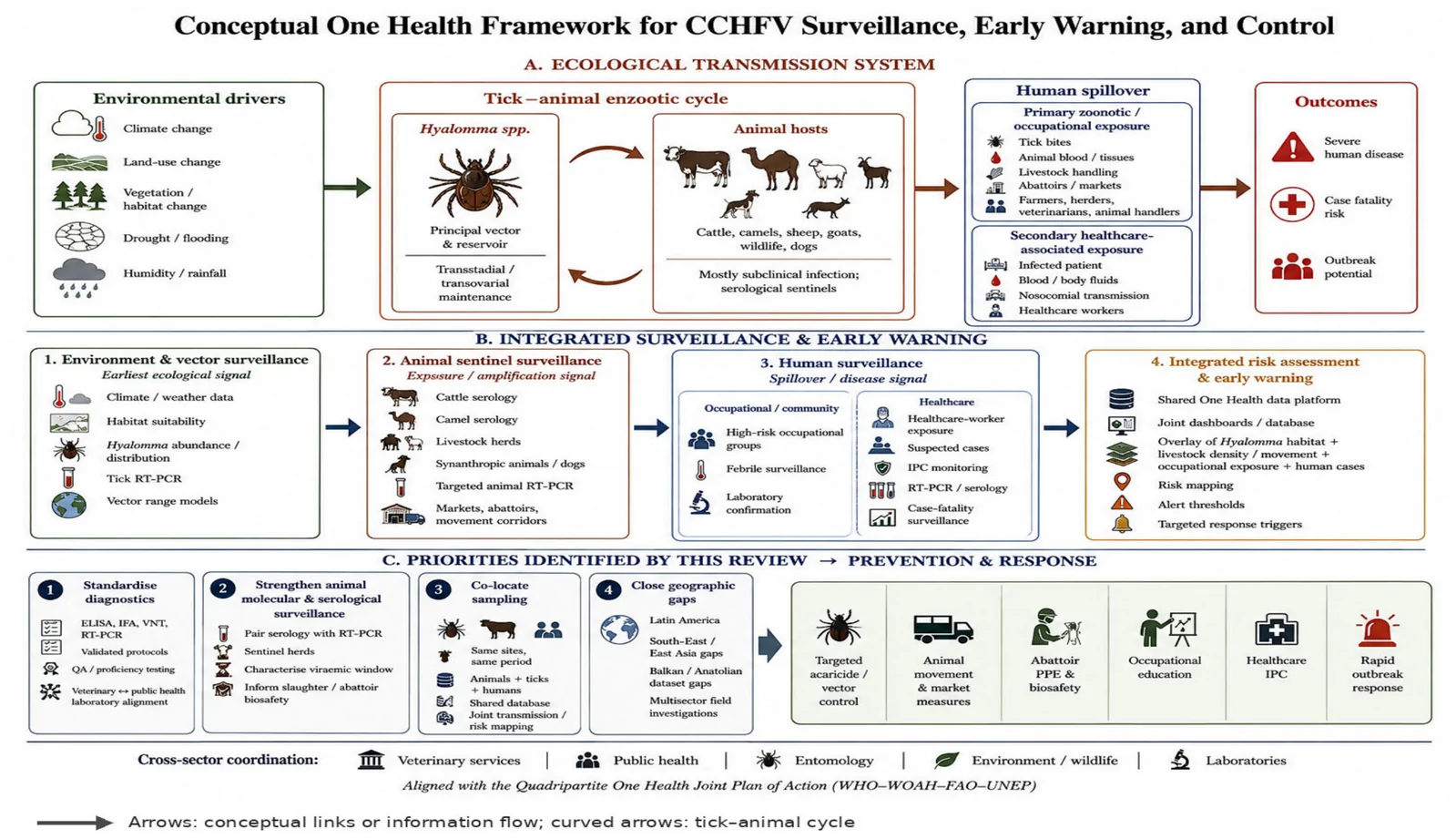
Proposed conceptual One Health framework for CCHFV surveillance.

**Table 1:** Descriptive overall anti-CCHFV antibody positivity among sampled animals by species with ≥3 publications providing species-specific raw counts. Overall proportion (%) = Σ positive/Σ tested; range = publication-level proportions.

Species	Studies (n)	Animals Tested (Σ)	Seropositivity Across Included Studies (%)	Study-Level Range (%)
Camel	23	5377	72.9	5.3–99.6
Red deer	6	2716	41.4	7.9–76.1
Buffalo	7	1171	24.5	0.0–75.4
Cattle	79	46,612	25.1	0.0–98.2
Wild boar	8	9456	18.3	1.4–40.6
Sheep	61	20,739	15.0	0.0–83.3
Goat	55	18,543	15.1	0.0–89.2
Donkey	4	848	11.8	5.8–31.4
Horse	5	3121	8.9	2.4–70.3
Dog	4	721	8.7	0.0–56.2
Rabbit/hare	4	1340	0.7	0.0–23.8

**Table 2:** Animal studies included in this review (n = 150).

Author, Year	Country/Region	Animal Species (n)	Diagnostic Method	Antibody Result	Antibody 95% CI	Viral Detection	Viral 95% CI
AlSaiari et al., 2026 [14]	Qatar	Camel: 182	ID Screen^®^ CCHF Double Antigen Multi-species ELISA (IDvet)	102/182 (56.0%)	48.8–63.1%	NA	NA
Beato-Benítez et al., 2026 [15]	Spain	White rhinoceros; camel—total: 956	ID Screen^®^ CCHF Double Antigen Multi-species ELISA (IDvet) + VNT	ELISA: 3/956 (0.3%); camel: 1/19 (5.3%); VNT: 1/3 (33.3%)	ELISA: 0.1–0.9%; camel: 0.9–23.6%; VNT: 6.1–79.2%	NA	NA
Beyit et al., 2026 [16]	Mauritania	Cattle: 174; camel: 143	ID Screen^®^ CCHF Double Antigen Multi-species ELISA (IDvet)	Overall: 222/317 (70.0%); cattle: 105/174 (60.3%); camel: 117/143 (81.8%)	Overall: 64.8–74.8%; cattle: 52.9–67.3%; camel: 74.7–87.3%	NA	NA
Ehizibolo et al., 2026 [17]	Nigeria	Horse: 305; donkey: 503	ID Screen^®^ CCHF Double Antigen Multi-species ELISA (IDvet)	Overall: 188/808 (23.3%); horse: 159/305 (52.1%); donkey: 29/503 (5.8%)	Overall: 20.5–26.3%; horse: 46.5–57.7%; donkey: 4.0–8.2%	NA	NA
Ehizibolo et al., 2026 [18]	Nigeria	Camel: 812	ID Screen^®^ CCHF Double Antigen Multi-species ELISA (IDvet)	726/812 (89.4%)	87.1–91.3%	NA	NA
Elmahallawy et al., 2026 [19]	Egypt	Camel: 513	ID Screen^®^ CCHF Double Antigen Multi-species ELISA (IDvet)	511/513 (99.6%)	98.6–99.9%	NA	NA
Ilboudo et al., 2026 [20]	Burkina Faso	Cattle: 666; sheep: 659; goat: 970	ID Screen^®^ CCHF Double Antigen Multi-species ELISA (IDvet)	Cattle: 360/666 (54.1%); sheep: 60/659 (9.1%); goat: 25/970 (2.6%)	Cattle: 50.3–57.8%; sheep: 7.1–11.5%; goat: 1.8–3.8%	NA	NA
Italiya et al., 2026 [21]	Bulgaria; Czech Republic; Romania	Bulgaria: Horse: 314; donkey: 265; Romania: Horse: 1534; Czech Republic: Horse: 576	ID Screen^®^ CCHF Double Antigen Multi-species ELISA (IDvet)	Bulgaria: Donkey: 50/265 (18.9%); horse: 34/314 (10.8%); Romania: Horse: 23/1534 (1.5%); Czech Republic: Horse: 0/576 (0.0%)	Bulgaria: Donkey: 14.6–24.0%; horse: 7.9–14.8%; Romania: Horse: 1.0–2.2%; Czech Republic: Horse: 0.0–0.7%	NA	NA
Italiya et al., 2026 [22]	Kosovo	Cattle: 100	ID Screen^®^ CCHF Double Antigen Multi-species ELISA (IDvet)	23/100 (23.0%)	15.8–32.2%	NA	NA
Koshemetov et al., 2026 [23]	Central Kazakhstan	Cattle: 350; small ruminants: 350	ID Screen^®^ CCHF Double Antigen Multi-species ELISA (IDvet) + RT-qPCR	Overall: 8/700 (1.1%)	0.6–2.2%	0/700 (0.0%)	0.0–0.5%
Lombe et al., 2026 [24]	Democratic Republic of Congo	Cattle: 706; sheep: 51; goat: 357	In-house CCHFV NP IgG ELISA	Overall: 322/1114 (28.9%); cattle: 302/706 (42.8%); sheep: 1/51 (2.0%); goat: 19/357 (5.3%)	Overall: 26.3–31.6%; cattle: 39.2–46.5%; sheep: 0.3–10.3%; goat: 3.4–8.2%	NA	NA
Cicculli et al., 2026 [25]	North Macedonia	Sheep: 1452; goat: 540	ID Screen^®^ CCHF Double Antigen Multi-species ELISA (IDvet)	Overall: 493/1992 (24.7%); sheep: 397/1452 (27.3%); goat: 96/540 (17.8%)	Overall: 22.9–26.7%; sheep: 25.1–29.7%; goat: 14.8–21.2%	NA	NA
Messahel et al., 2026 [26]	Eastern Algerian Sahara	Dromedary camel: 73	ID Screen^®^ CCHF Double Antigen Multi-species ELISA (IDvet)	Dromedary camel: 55/73 (75.3%)	Dromedary camel: 64.4–83.8%	NA	NA
Myrzakhmetova et al., 2026 [27]	Turkestan Region, Kazakhstan	Cattle: 840	ID Screen^®^ CCHF Double Antigen Multi-species ELISA (IDvet)	Overall: 304/840 (36.2%); spring: 181/420 (43.09%); autumn: 123/420 (29.28%)	Overall: 33.0–39.5%; spring: 38.4–47.9%; autumn: 25.1–33.8%	NA	NA
Nyakarahuka et al., 2026 [28]	Uganda	Goat: 26	CDC in-house CCHFV IgG ELISA + RT-PCR	23/26 (88.5%)	71.0–96.0%	0/26 (0.0%)	0.0–12.9%
Rasheid et al., 2026 [29]	Iraq	Cattle: 806; sheep: 1820; goat: 1713	CCHFV IgG ELISA (Vector-Best)	Overall: 276/4339 (6.4%); cattle: 141/806 (17.5%); sheep: 53/1820 (2.9%); goat: 82/1713 (4.8%)	Overall: 5.7–7.1%; cattle: 15.0–20.3%; sheep: 2.2–3.8%; goat: 3.9–5.9%	NA	NA
Sulaiman et al., 2026 [30]	Sulaymaniyah, Iraq	Cattle: 150; sheep: 149; goat: 151	ID Screen^®^ CCHF Double Antigen Multi-species ELISA (IDvet)	Cattle: 41/150 (27.3%); sheep: 46/149 (30.9%); goat: 58/151 (38.4%)	Cattle: 20.8–35.0%; sheep: 24.0–38.7%; goat: 31.0–46.4%	NA	NA
Xavier et al., 2026 [31]	Portugal; Spain	Red deer: 447; wild boar: 1014	ID Screen^®^ CCHF Double Antigen Multi-species ELISA (IDvet)	Red deer: 322/447 (72.0%); wild boar: 328/1014 (32.3%)	Red deer: 67.7–76.0%; wild boar: 29.5–35.3%	NA	NA
Ammar et al., 2025 [32]	Pakistan	Buffalo: 90; cattle: 200; sheep: 5; goat: 12; dog: 14	CCHFV NP LIPS + Western blot + microneutralization	LIPS: 10/321 (3.1%); neutralization: 1/321 (0.3%)	LIPS: 1.7–5.6%; neutralization: 0.1–1.7%	NA	NA
Baptista et al., 2025 [33]	Portugal	Wild European rabbit: 667	ID Screen^®^ CCHF Double Antigen Multi-species ELISA (IDvet)	4/667 (0.6%)	0.2–1.5%	NA	NA
Bennia et al., 2025 [34]	Northeastern Algeria	Sheep: 276	ID Screen^®^ CCHF Double Antigen Multi-species ELISA (IDvet)	108/276 (39.1%)	33.6–45.0%	NA	NA
Bernard et al., 2025 [35]	Southern continental France	Cattle: 8609; wild boar: 869; roe deer: 977; red deer: 252; mouflon: 22; fox: 62	ID Screen^®^ CCHF Double Antigen Multi-species ELISA (IDvet) + PPRNT	Cattle: 176/8609 (2.0%); wildlife: 49/2182 (2.2%); red deer: 20/252 (7.9%); roe deer: 16/977 (1.6%); wild boar: 12/869 (1.4%); mouflon: 0/22 (0.0%); fox: 1/62 (1.6%)	Cattle: 1.8–2.4%; wildlife overall: 1.7–3.0%; red deer: 5.2–11.9%; roe deer: 1.0–2.6%; wild boar: 0.8–2.4%; mouflon: 0.0–14.9%; fox: 0.3–8.6%	NA	NA
Chinyere et al., 2025 [36]	Nigeria (Abia, Kano, Plateau and Sokoto States)	Cattle: 105; goat: 111; sheep: 91; camel: 160	ID Screen^®^ CCHF Double Antigen Multi-species ELISA (IDvet)	Overall: 176/467 (37.7%); cattle: 22/105 (21.0%); goat: 12/111 (10.8%); sheep: 7/91 (7.7%); camel: 135/160 (84.4%)	Overall: 33.4–42.2%; cattle: 14.3–29.7%; goat: 6.3–18.0%; sheep: 3.8–15.0%; camel: 78.0–89.2%	NA	NA
de Villiers et al., 2025 [37]	Namibia	Dog: 374; cat: 238	ID Screen^®^ CCHF Double Antigen Multi-species ELISA (IDvet)	Overall: 47/612 (7.7%); dog: 43/374 (11.5%); cat: 4/238 (1.7%)	Overall: 5.8–10.1%; dog: 8.6–15.1%; cat: 0.7–4.2%	NA	NA
Dos Santos et al., 2025 [38]	Northeastern Portugal	Red deer: 10; cattle: 94; sheep: 30; goat: 4	ID Screen^®^ CCHF Double Antigen Multi-species ELISA (IDvet) + IFA + RT-qPCR (S segment)	Overall: 43/138 (31.2%); red deer: 6/10 (60.0%); cattle: 36/94 (38.3%); sheep: 1/30 (3.3%); goat: 0/4 (0.0%)	Overall: 24.0–39.3%; red deer: 31.3–83.2%; cattle: 29.1–48.4%; sheep: 0.6–16.7%; goat: 0.0–49.0%	0/90 (0.0%)	0.0–4.1%
Ehizibolo et al., 2025 [39]	Nigeria	Cattle: 1150; sheep: 1150; goat: 1150	ID Screen^®^ CCHF Double Antigen Multi-species ELISA (IDvet)	Overall: 936/3450 (27.1%); cattle: 637/1150 (55.4%); sheep: 200/1150 (17.4%); goat: 99/1150 (8.6%)	Overall: 25.7–28.6%; cattle: 52.5–58.2%; sheep: 15.3–19.7%; goat: 7.1–10.4%	NA	NA
Fafetine et al., 2025 [40]	Mozambique	Cattle: 460	ID Screen^®^ CCHF Double Antigen Multi-species ELISA (IDvet)	230/460 (50.0%)	45.4–54.6%	NA	NA
Gasparine et al., 2025 [41]	Cameroon (Centre Region)	Cattle: 386; goat: 148	ID Screen^®^ CCHF Double Antigen Multi-species ELISA (IDvet) + RT-qPCR (L segment)	Overall: 47/534 (8.8%); cattle: 42/386 (10.9%); goat: 5/148 (3.4%)	Overall: 6.7–11.5%; cattle: 8.2–14.4%; goat: 1.5–7.7%	0/534 (0.0%)	0.0–0.7%
Halbrook et al., 2025 [42]	Democratic Republic of Congo	Cattle: 1118; pig: 1020	In-house CCHFV NP IgG ELISA	Cattle: 637/1118 (57.0%); pig: 134/1020 (13.1%)	Cattle: 54.1–59.9%; pig: 11.2–15.3%	NA	NA
Jakimovski et al., 2025 [43]	North Macedonia	Sheep: 8; goat: 9	ID Screen^®^ CCHF Double Antigen Multi-species ELISA (IDvet)	10/17 (58.8%)	36.0–78.4%	NA	NA
Leil et al., 2025 [44]	Wasit Governorate, Iraq	Sheep: 104; goat: 92	ID Screen^®^ CCHF Double Antigen Multi-species ELISA (IDvet)	Overall: 73/196 (37.2%); sheep: 37/104 (35.6%); goat: 36/92 (39.1%)	Overall: 30.8–44.2%; sheep: 27.0–45.1%; goat: 29.8–49.3%	NA	NA
Limon et al., 2025 [45]	Bulgaria	Sheep: 1200	In-house CCHFV NP/Gc IgG ELISAs	Weighted NP IgG: 38.5%	35.3–42.0%	NA	NA
Miletic et al., 2025 [46]	Croatia	Cattle: 276; sheep: 336; goat: 57; horse: 304; dog: 304; cat: 196	ID Screen^®^ CCHF Double Antigen Multi-species ELISA (IDvet)	Overall: 109/1473 (7.4%); cattle: 0/276 (0.0%); sheep: 95/336 (28.3%); goat: 0/57 (0.0%); horse: 13/304 (4.3%); dog: 0/304 (0.0%); cat: 1/196 (0.5%)	Overall: 6.2–8.9%; cattle: 0.0–1.4%; sheep: 23.7–33.3%; goat: 0.0–6.3%; horse: 2.5–7.2%; dog: 0.0–1.2%; cat: 0.1–2.8%	NA	NA
Mohammed et al., 2025 [47]	Nineveh Province, Iraq	Cattle: 124	ID Screen^®^ CCHF Double Antigen Multi-species ELISA (IDvet)	60/124 (48.4%)	39.8–57.1%	NA	NA
Plebani et al., 2025 [48]	Namibia	Leopard: 250; lion: 7	ID Screen^®^ CCHF Double Antigen Multi-species ELISA (IDvet)	Leopard: 36/250 (14.4%); lion: 3/7 (42.9%)	Leopard: 10.6–19.3%; lion: 15.8–75.0%	NA	NA
Raheemi et al., 2025 [49]	Afghanistan	Cattle: 384; sheep: 384; goat: 384	ID Screen^®^ CCHF Double Antigen Multi-species ELISA (IDvet)	Overall: 144/1152 (12.5%); cattle: 21.9%; sheep: 5.2%; goat: 10.6%	Overall: 10.7–14.5%; cattle: 18.0–26.3%; sheep: 3.4–7.9%; goat: 8.0–14.2%	NA	NA
Rinchen et al., 2025 [50]	Southern Bhutan	Goat: 472	In-house CCHFV ELISA + IFA	Overall: 163/472 (34.5%); Samtse: 67/153 (43.8%); Sarpang: 49/81 (60.5%); Chukha: 39/123 (31.7%); Dagana: 6/74 (8.1%); Tsirang: 2/41 (4.9%)	Overall: 30.4–38.9%; Samtse: 36.2–51.7%; Sarpang: 49.6–70.4%; Chukha: 24.1–40.4%; Dagana: 3.8–16.6%; Tsirang: 1.3–16.1%	NA	NA
Rudoler et al., 2025 [51]	Israel	Cattle: 536; wildlife: 200	ID Screen^®^ CCHF Double Antigen Multi-species ELISA (IDvet)	Cattle: 181/536 (33.8%); herds: 16/19 (84.2%); wildlife: 2/200 (1.0%)	Cattle: 29.9–37.9%; herds: 62.4–94.5%; wildlife: 0.3–3.6%	NA	NA
Sadio et al., 2025 [52]	Kédougou, southeastern Senegal	Cattle: 15; sheep: 35; goat: 35	ID Screen^®^ CCHF Double Antigen Multi-species ELISA (IDvet) + RT-PCR	Overall: 12/85 (14.1%); cattle: 7/15 (46.7%); sheep: 1/35 (2.9%); goat: 4/35 (11.4%)	Overall: 8.3–23.1%; cattle: 24.8–69.9%; sheep: 0.5–14.5%; goat: 4.5–26.0%	NR	—
Saleh et al., 2025 [53]	Thi-Qar Governorate, Iraq	Sheep: 242; goat: 62	ID Screen^®^ CCHF Double Antigen Multi-species ELISA (IDvet)	Overall: 150/304 (49.3%); sheep: 128/242 (52.9%); goat: 22/62 (35.5%)	Overall: 43.8–54.9%; sheep: 46.6–59.1%; goat: 24.7–47.9%	NA	NA
Sana et al., 2025 [54]	Pakistan (multiple regions)	Goats; sheep; cattle; water buffaloes; rats Balochistan: 200; Khyber Pakhtunkhwa: 150; Sindh: 201; Kashmir: 50; Punjab: 434	In-house CCHFV NP ELISA + ELISA	Balochistan: 20/200 (10.0%); Khyber Pakhtunkhwa: 14/150 (9.3%); Sindh: 8/201 (4.0%); Kashmir: 1/50 (2.0%); Punjab: 55/434 (12.7%)	Balochistan: 6.6–14.9%; Khyber Pakhtunkhwa: 5.6–15.1%; Sindh: 2.0–7.7%; Kashmir: 0.4–10.5%; Punjab: 9.9–16.1%	NA	NA
Sheek-Hussein et al., 2025 [55]	United Arab Emirates	Dromedary camel: 393	ID Screen^®^ CCHF Double Antigen Multi-species ELISA (IDvet) + RT-qPCR	256/393 (65.1%)	60.3–69.7%	4/393 (1.0%)	0.4–2.6%
Sánchez-Sánchez et al., 2025 [56]	Iberian Peninsula (Spain/Portugal conservation population)	Iberian lynx: 554	CCHFV NP double-antigen ELISA + VNT + nested RT-PCR	66/554 (11.9%)	9.5–14.9%	0/48 (0.0%)	0.0–7.4%
Yessinou et al., 2025 [57]	Benin	Wild: 254; domestic: 112	ID Screen^®^ CCHF Double Antigen Multi-species ELISA (IDvet)	Overall: 41/366 (11.2%); domestic: 38/112 (33.9%); cattle: 34/81 (42.0%); horse: 4/24 (16.7%); wild: 3/254 (1.2%); giant rat: 1/84 (1.2%); squirrel: 1/27 (3.7%); hare: 1/17 (5.9%)	Overall: 8.4–14.8%; domestic: 25.8–43.1%; cattle: 31.8–52.8%; horse: 6.7–35.9%; wild: 0.4–3.4%; giant rat: 0.2–6.4%; squirrel: 0.7–18.3%; hare: 1.0–27.0%	NA	NA
Sruthy et al., 2025 [58]	Wayanad, Kerala, India	Buffalo: 4; cattle: 198; goat: 98	ID Screen^®^ CCHF Double Antigen Multi-species ELISA (IDvet)	Overall: 7/300 (2.3%); buffalo: 0/4 (0.0%); cattle: 2/198 (1.0%); goat: 5/98 (5.1%)	Overall: 1.1–4.7%; buffalo: 0.0–49.0%; cattle: 0.3–3.6%; goat: 2.2–11.4%	NA	NA
Adamu et al., 2024 [59]	Nigeria	Dromedary camel: 184	ID Screen^®^ CCHF Double Antigen Multi-species ELISA (IDvet)	179/184 (97.3%)	93.8–98.8%	NA	NA
Baz-Flores et al., 2024 [60]	Andalusia, Spain	Sheep: 1220; goat: 1220	ID Screen^®^ CCHF Double Antigen Multi-species ELISA (IDvet)	Overall: 84/2440 (3.4%); sheep: 36/1220 (3.0%); goat: 48/1220 (3.9%)	Overall: 2.8–4.2%; sheep: 2.1–4.1%; goat: 3.0–5.2%	NA	NA
Baz-Flores et al., 2024 [61]	Portugal; Spain	Wild boar: 5291	ID Screen^®^ CCHF Double Antigen Multi-species ELISA (IDvet)	1026/5291 (19.4%)	18.3–20.5%	NA	NA
Cevidanes et al., 2024 [62]	Basque Country, northern Spain	Red deer: 36; roe deer: 36; wild boar: 1190	ID Screen^®^ CCHF Double Antigen Multi-species ELISA (IDvet)	Overall: 32/1262 (2.5%); red deer: 8/36 (22.2%); roe deer: 3/36 (8.3%); wild boar: 21/1190 (1.8%)	Overall: 1.8–3.6%; red deer: 11.7–38.1%; roe deer: 2.9–21.8%; wild boar: 1.2–2.7%	NA	NA
Dahourou et al., 2024 [63]	Kénédougou and Mouhoun Provinces, Burkina Faso	Cattle: 371	ID Screen^®^ CCHF Double Antigen Multi-species ELISA (IDvet)	268/371 (72.2%)	67.5–76.5%	NA	NA
Daodu et al., 2024 [64]	Kwara State, Nigeria	Cattle: 877	In-house CCHFV NP IgG ELISA + CCHFV IgG ELISA (Vector-Best) + ID Screen^®^ CCHF Double Antigen Multi-species ELISA (IDvet)	631/877 (71.9%)	68.9–74.8%	NA	NA
Degui et al., 2024 [65]	Southern Algeria	Dromedary camel: 269	ID Screen^®^ CCHF Double Antigen Multi-species ELISA (IDvet)	255/269 (94.8%)	91.5–96.9%	NA	NA
Deézsi-Magyar et al., 2024 [66]	Hungary	Cattle: 1391; sheep: 514	Cattle: Cow CCHFV IgG ELISA (Abbexa) + in-house IgG IIFA + CCHFV Mosaic 2 IgG IFA (Euroimmun); sheep: in-house IgG IIFA + CCHFV Mosaic 2 IgG IFA (Euroimmun)	Overall: 11/1905 (0.6%); cattle: 8/1391 (0.6%); sheep: 3/514 (0.6%)	Overall: 0.3–1.0%; cattle: 0.3–1.1%; sheep: 0.2–1.7%	NA	NA
Diakite et al., 2024 [67]	Mopti Region, Mali	Cattle: 99; sheep: 100	ID Screen^®^ CCHF Double Antigen Multi-species ELISA (IDvet)	Overall: 85/199 (42.7%); cattle: 58/99 (58.6%); sheep: 27/100 (27.0%)	Overall: 36.0–49.7%; cattle: 48.7–67.8%; sheep: 19.3–36.4%	NA	NA
Frías et al., 2024 [68]	Spain	Iberian pig: 251; wild boar: 267	ID Screen^®^ CCHF Double Antigen Multi-species ELISA (IDvet) + in-house CCHFV NP IgG ELISA + RT-qPCR (S segment)	Overall: 113/518 (21.8%); Iberian pigs: 7/251 (2.8%); wild boar: 106/267 (39.7%)	Overall: 18.5–25.6%; Iberian pigs: 1.4–5.6%; wild boar: 34.0–45.7%	0/462 (0.0%)	0.0–0.8%
Gahn et al., 2024 [69]	Senegal (national)	Sheep: 1130; goat: 997	ID Screen^®^ CCHF Double Antigen Multi-species ELISA (IDvet)	Overall: 297/2127 (14.0%); sheep: 183/1130 (16.2%); goat: 114/997 (11.4%).	Overall: 12.6–15.5%; sheep: 14.2–18.5%; goat: 9.6–13.6%	NA	NA
Hughes et al., 2024 [70]	Northern Tanzania	Cattle: 3015; sheep: 2059; goat: 2382	ID Screen^®^ CCHF Double Antigen Multi-species ELISA (IDvet)	Cattle: 49.6%; sheep: 27.8%; goat: 33.8%	Cattle: 40.0–59.2%; sheep: 17.0–40.6%; goat: 21.7–47.5%	NA	NA
Manjunathachar et al., 2024 [71]	Jabalpur, Madhya Pradesh, India	Cattle: 155; goat: 238	CCHFV IgG ELISA (Zydus Diagnostics)	Overall: 63/393 (16.0%); cattle: 18/155 (11.6%); goat: 45/238 (18.9%)	Overall: 12.7–20.0%; cattle: 7.5–17.6%; goat: 14.4–24.4%	NA	NA
Mukhaye et al., 2024 [72]	Isiolo County, Kenya	Cattle: 160; sheep: 750; goat: 841; camel: 97; giraffe: 12; buffalo: 16; zebra: 15; warthog: 15; waterbuck: 6; oryx: 18; impala: 3	ID Screen^®^ CCHF Double Antigen Multi-species ELISA (IDvet) + RT-qPCR	Cattle: 100/160 (62.5%); sheep: 66/750 (8.8%); goat: 96/841 (11.4%); camel: 87/97 (89.7%); giraffe: 9/12 (75.0%); buffalo: 10/16 (62.5%); zebra: 6/15 (40.0%); warthog: 3/15 (20.0%); waterbuck: 1/6 (16.7%); oryx: 6/18 (33.3%); impala: 0/3 (0.0%)	Cattle: 54.8–69.6%; sheep: 7.0–11.0%; goat: 9.4–13.7%; camel: 82.1–94.3%; giraffe: 46.8–91.1%; buffalo: 38.6–81.5%; zebra: 19.8–64.3%; warthog: 7.0–45.2%; waterbuck: 3.0–56.4%; oryx: 16.3–56.3%; impala: 0.0–56.2%	Cattle: 6/42 (14.3%); sheep: 0/16 (0.0%); goat: 1/26 (3.8%); camel: 24/80 (30.0%); wild animals: 0/7 (0.0%)	Cattle: 6.7–27.8%; sheep: 0.0–19.4%; goat: 0.7–18.9%; camel: 21.1–40.8%; wild animals: 0.0–35.4%
Ngom et al., 2024 [73]	Podor, northern Senegal	Cattle: 56; sheep: 79; goat: 66	ID Screen^®^ CCHF Double Antigen Multi-species ELISA (IDvet)	Overall: 87/201 (43.3%); cattle: 45/56 (80.4%); sheep: 28/79 (35.4%); goat: 14/66 (21.2%)	Overall: 36.6–50.2%; cattle: 68.2–88.7%; sheep: 25.8–46.4%; goat: 13.1–32.5%	NA	NA
Raheemi et al., 2024 [74]	Nangarhar and Kunar Provinces, Afghanistan	Cattle: 384; sheep: 192; goat: 192	ID Screen^®^ CCHF Double Antigen Multi-species ELISA (IDvet)	Overall: 146/768 (19.0%); cattle: 86/384 (22.4%); sheep: 25/192 (13.0%); goat: 35/192 (18.2%)	Overall: 16.4–21.9%; cattle: 18.5–26.8%; sheep: 9.0–18.5%; goat: 13.4–24.3%	NA	NA
Samkange et al., 2024 [75]	Omaheke Region, Namibia	Cattle: 100; sheep: 100	ID Screen^®^ CCHF Double Antigen Multi-species ELISA (IDvet)	Overall: 58/200 (29.0%); cattle: 36/100 (36.0%); sheep: 22/100 (22.0%)	Overall: 23.2–35.6%; cattle: 27.3–45.8%; sheep: 15.0–31.1%	NA	NA
Sene et al., 2024 [76]	Bokidiawe and Koumpentoum, eastern Senegal	Bokidiawe — Sheep: 73; goat: 22; Koumpentoum — Sheep: 18; goat: 57	In-house recombinant CCHFV glycoprotein Gn IgG ELISA + RT-PCR	Bokidiawe: 43/95 (45.3%) [Sheep: 27/73 (37.0%); goat: 16/22 (72.7%)]. Koumpentoum: 7/75 (9.3%) [Sheep: 2/18 (11.1%); goat: 2/57 (3.5%)]	Bokidiawe: 35.6–55.3%; sheep: 26.8–48.5%; goat: 51.8–86.8%. Koumpentoum: 7/75: 4.6–18.0%; sheep: 3.1–32.8%; goat: 1.0–11.9%	0/170 (0.0%)	0.0–2.2%
Simo et al., 2024 [77]	West and Centre Cameroon	Cattle: 423	ID Screen^®^ CCHF Double Antigen Multi-species ELISA (IDvet)	116/423 (27.4%)	23.4–31.9%	NA	NA
Dahourou et al., 2024 [78]	Mouhoun and Kénédougou Provinces, Burkina Faso	Sheep: 364	ID Screen^®^ CCHF Double Antigen Multi-species ELISA (IDvet)	54/364 (14.8%)	11.6–18.9%	NA	NA
Castro-Scholten et al., 2024 [79]	Spain	European wild rabbit: 473; Iberian hare: 162	IDvet Screen^®^ multi-species double-antigen ELISA	0/635 (0.0%)	0.0–0.6%	NA	NA
Altaliby et al., 2023 [80]	Iraq	Sheep: 120; goat: 80	ID Screen^®^ CCHF Double Antigen Multi-species ELISA (IDvet)	Overall: 28/200 (14.0%); sheep: 23/120 (19.2%); goat: 5/80 (6.3%)	Overall: 9.9–19.5%; sheep: 13.1–27.1%; goat: 2.7–13.8%	NA	NA
Atim et al., 2023 [81]	Lyantonde District, Uganda	Cattle: 117; goat: 93	ID Screen^®^ CCHF Double Antigen Multi-species ELISA (IDvet) + RT-qPCR + NGS	Overall: 193/210 (91.9%); cattle: 110/117 (94.0%); goat: 83/93 (89.2%)	Overall: 87.4–94.9%; cattle: 88.2–97.1%; goat: 81.3–94.1%	0/210 (0.0%)	0.0–1.8%
Babaoglu et al., 2023 [82]	Van Province, Türkiye	Cattle: 276; sheep: 166; goat: 49	ID Screen^®^ CCHF Double Antigen Multi-species ELISA (IDvet) + RT-PCR	Overall: 179/491 (36.5%); cattle: 32/276 (11.6%); sheep: 107/166 (64.5%); goat: 40/49 (81.6%)	Overall: 32.3–40.8%; cattle: 8.3–15.9%; sheep: 56.9–71.3%; goat: 68.6–90.0%	6/491 (1.2%)	0.6–2.6%
El Ghassem et al., 2023 [83]	Mauritania	Sheep: 502; goat: 477; dromedary camel: 161; cattle: 240	ID Screen^®^ CCHF Double Antigen Multi-species ELISA (IDvet)	Overall: 457/1380 (33.1%); sheep: 91/502 (18.1%); goat: 43/477 (9.0%); camel: 144/161 (89.4%); cattle: 179/240 (74.6%)	Overall: 30.7–35.6%; sheep: 15.0–21.7%; goat: 6.8–11.9%; camel: 83.7–93.3%; cattle: 68.7–79.7%	NA	NA
Guidoum et al., 2023 [84]	Northeastern Saharan Algeria	Dromedary camel: 294	ID Screen^®^ CCHF Double Antigen Multi-species ELISA (IDvet)	Animal-level: 222/294 (75.5%); herds: 22/23 (95.7%)	Animal-level: 70.3–80.1%; herd-level: 79.0–99.2%	NA	NA
Matthews et al., 2023 [85]	The Gambia	Sheep: 470; goat: 544; cattle: 399	ID Screen^®^ CCHF Double Antigen Multi-species ELISA (IDvet)	Overall: 377/1413 (26.7%); sheep: 89/470 (18.9%); goat: 49/544 (9.0%); cattle: 239/399 (59.9%)	Overall: 24.4–29.0%; sheep: 15.7–22.7%; goat: 6.9–11.7%; cattle: 55.0–64.6%	NA	NA
Mostafa et al., 2023 [86]	Iraq (slaughterhouse setting)	Sheep: 279; goat: 130	ID Screen^®^ CCHF Double Antigen Multi-species ELISA (IDvet)	Overall: 79/409 (19.3%); sheep: 61/279 (21.9%); goat: 18/130 (13.8%)	Overall: 15.8–23.4%; sheep: 17.4–27.1%; goat: 8.9–20.8%	NA	NA
Nyakarahuka et al., 2023 [87]	Uganda	Cattle: 1732; sheep: 358; goat: 1091	In-house CCHFV IgG ELISA	Overall: 999/3181 (31.4%); cattle: 292/1732 (16.9%); sheep: 176/358 (49.2%); goat: 531/1091 (48.7%)	Overall: 29.8–33.0%; cattle: 15.2–18.7%; sheep: 44.0–54.3%; goat: 45.7–51.6%	NA	NA
Omoga et al., 2023 [88]	Kenya	Cattle: 310; sheep: 295; goat: 295; donkey: 51; rodent: 93	ID Screen^®^ CCHF Double Antigen Multi-species ELISA (IDvet) + RT-PCR (S segment) + sequencing	Overall: 113/951 (11.9%); cattle: 44/310 (14.2%); sheep: 29/295 (9.8%); goat: 24/295 (8.1%); donkey: 16/51 (31.4%); rodent: 6/93 (6.5%)	Overall: 10.0–14.1%; cattle: 10.7–18.5%; sheep: 6.9–13.8%; goat: 5.5–11.8%; donkey: 20.3–45.0%; rodent: 3.0–13.4%	Sheep pooled sera: 4/280 (1.4%); rodent tissues: 4/480 (0.8%); cattle/goat/donkey pools negative	Sheep pooled sera: 0.6–3.6%; rodent tissues: 0.3–2.1%
Sajid et al., 2023 [89]	District Mardan, Pakistan	Cattle: 164; buffalo: 124; goat: 73; sheep: 20	CCHFV IgG indirect ELISA	Overall: 58/381 (15.2%); cattle: 23/164 (14.0%); buffalo: 24/124 (19.4%); goat: 8/73 (11.0%); sheep: 3/20 (15.0%)	Overall: 12.0–19.2%; cattle: 9.5–20.2%; buffalo: 13.4–27.2%; goat: 5.7–20.2%; sheep: 5.2–36.0%	NA	NA
Sarangi et al., 2023 [90]	India	Buffalo: 253; cattle: 551	ID Screen^®^ CCHF Double Antigen Multi-species ELISA (IDvet)	Overall apparent: 76/804 (9.5%); buffalo: 17/253 (6.7%); cattle: 59/551 (10.7%).	Overall: 7.6–11.7%; buffalo: 4.2–10.5%; cattle: 8.4–13.6%	NA	NA
Simo Tchetgna et al., 2023 [91]	Cameroon	Cattle: 441; sheep: 147; goat: 168	ID Screen^®^ CCHF Double Antigen Multi-species ELISA (IDvet) + tecVLP neutralization + RT-qPCR (S segment)	Overall: 467/756 (61.8%); cattle: 433/441 (98.2%); sheep: 23/147 (15.6%); goat: 11/168 (6.5%)	Overall: 58.3–65.2%; cattle: 96.5–99.1%; sheep: 10.7–22.4%; goat: 3.7–11.3%	0 positive	—
Wernery et al., 2023 [92]	United Arab Emirates; Mongolia; Pakistan	UAE racing dromedary: 44; UAE breeding dromedary: 50; hybrid camel: 9; Bactrian camel: 9; Pakistan dromedary: 38	Double-antigen indirect ELISA	Overall: 90/150 (60.0%); UAE racing: 9/44 (20.5%); UAE breeding 28/50 (56.0%); UAE hybrids: 7/9 (77.8%); Bactrian camels: 8/9 (88.9%); Pakistan dromedaries: 38/38 (100.0%)	Overall: 52.0–67.5%; UAE racing: 11.2–34.5%; UAE breeding: 42.3–68.8%; UAE hybrids: 45.3–93.7%; Bactrian: 56.5–98.0%; Pakistan dromedaries: 90.8–100.0%	NA	NA
Weidinger et al., 2023 [93]	United Arab Emirates	Wild rodents: 64 sampled (52 gerbils, 1 jird, 10 house mice, 1 Arabian spiny mouse)	RT-qPCR using RealStar CCHFV RT-PCR Kit 1.0 (altona Diagnostics)	NA	NA	No CCHFV RNA detected in tested rodent-derived samples	Not calculated (no single tested-unit denominator reported)
Atim et al., 2022 [94]	Uganda	Cattle: 666; goat: 549; dog: 32	ID Screen^®^ CCHF Double Antigen Multi-species ELISA (IDvet) + in-house CCHFV NP IgG ELISA	Overall: 1043/1247 (83.6%); cattle: 612/666 (91.9%); goat: 413/549 (75.2%); dog: 18/32 (56.2%)	Overall: 81.5–85.6%; cattle: 89.6–93.7%; goat: 71.4–78.7%; dog: 39.3–71.8%	NA	NA
Bryant-Genevier et al., 2022 [95]	Zhambyl Region, Kazakhstan	Sheep: 521; cattle: 454	CDC in-house CCHFV IgG ELISA	Sheep: 65/521 (12.5%); cattle: 132/454 (29.1%)	Sheep: 9.9–15.6%; cattle: 25.1–33.4%	NA	NA
Carrera-Faja et al., 2022 [96]	Valencia Region, Spain	Iberian ibex: 126; mouflon: 48; wild boar: 332	ID Screen^®^ CCHF Double Antigen Multi-species ELISA (IDvet)	Overall: 220/506 (43.5%); Iberian ibex: 121/126 (96.0%); mouflon: 48/48 (100.0%); wild boar: 51/332 (15.4%)	Overall: 39.2–47.8%; Iberian ibex: 91.0–98.3%; mouflon: 92.6–100.0%; wild boar: 11.9–19.6%	NA	NA
Cuadrado-Matías et al., 2022 [97]	Doñana National Park, Spain	Red deer: 527; wild boar: 453	ID Screen^®^ CCHF Double Antigen Multi-species ELISA (IDvet)	Overall: 585/980 (59.7%); red deer: 401/527 (76.1%); wild boar: 184/453 (40.6%)	Overall: 56.6–62.7%; red deer: 72.3–79.5%; wild boar: 36.2–45.2%	NA	NA
Cuadrado-Matías et al., 2022 [98]	Portugal; Spain	Red deer: 1444	ID Screen^®^ CCHF Double Antigen Multi-species ELISA (IDvet)	367/1444 (25.4%); seropositive population: 50/82 (61.0%)	Animal-level: 23.2–27.7%; population-level: 50.2–70.8%	NA	NA
Dzikwi-Emennaa et al., 2022 [99]	Plateau State, Nigeria	Cattle: 184	ID Screen^®^ CCHF Double Antigen Multi-species ELISA (IDvet)	56/184 (30.4%)	24.2–37.4%	NA	NA
Fanelli et al., 2022 [100]	Basilicata Region, southern Italy	Cattle: 794	ID Screen^®^ CCHF Double Antigen Multi-species ELISA (IDvet)	Animal-level: 15/794 (1.9%); herds: 8/27 (29.6%)	Animal-level: 1.1–3.1%; herd-level: 15.9–48.5%	NA	NA
Goletić et al., 2022 [101]	Bosnia and Herzegovina	Cattle: 481; RT-qPCR subset: 206	ID Screen^®^ CCHF Double Antigen Multi-species ELISA (IDvet) + RT-qPCR (S segment)	72/481 (15.0%); herds: 10/72 (13.9%)	Animal-level: 12.1–18.4%; herd-level: 7.7–23.7%	0/206 (0.0%)	0.0–1.8%
González Gordon et al., 2022 [102]	Cameroon	Pastoral cattle: 1498; dairy cattle: 60	ID Screen^®^ CCHF Double Antigen Multi-species ELISA (IDvet)	Pastoral cattle: 807/1498 (53.9%); dairy cattle: 4/60 (6.7%)	Pastoral: 51.3–56.4%; dairy: 2.6–15.9%	NA	NA
Lule et al., 2022 [103]	Uganda	Cattle: 419	ID Screen^®^ CCHF Double Antigen Multi-species ELISA (IDvet)	292/419 (69.7%)	65.1–73.9%	NA	NA
Lysholm et al., 2022 [104]	Zambia	Goat: 962	ID Screen^®^ CCHF Double Antigen Multi-species ELISA (IDvet)	33/962 (3.4%)	2.5–4.8%	NA	NA
Mesquita et al., 2022 [105]	Portugal	Sheep: 459	ID Screen^®^ CCHF Double Antigen Multi-species ELISA (IDvet)	2/459 (0.4%)	0.1–1.6%	NA	NA
Mhamadi et al., 2022 [106]	Agnam, northeastern Senegal	Sheep: 34; sera: 453	In-house recombinant CCHFV glycoprotein Gn IgG ELISA + RT-qPCR	Sheep: 13/34 (38.2%)	Sheep: 23.9–55.0%	0/453 (0.0%)	0.0–0.8%
Canakoglu et al., 2022 [107]	Türkiye	Wild boar: 40; cattle: 259; sheep: 130; goat: 132; hare: 21	In-house CCHFV IgG ELISA	Overall: 79/582 (13.6%); wild boar: 1/40 (2.5%); cattle: 28/259 (10.8%); sheep: 25/130 (19.2%); goat: 20/132 (15.2%); hares: 5/21 (23.8%)	Overall: 11.0–16.6%; wild boar: 0.4–12.9%; cattle: 7.6–15.2%; sheep: 13.4–26.8%; goat: 10.0–22.2%; hares: 10.6–45.1%	NA	NA
Šatrović et al., 2022 [108]	Bosnia and Herzegovina	Sheep: 176	ID Screen^®^ CCHF Double Antigen Multi-species ELISA (IDvet) + CCHFV Mosaic 2 IFA (Euroimmun)	ELISA: 17/176 (9.7%); IFA: 13/17 (76.5%)	ELISA: 6.1–14.9%; IFA: 52.7–90.4%	NA	NA
Zhabari et al., 2022 [109]	Malisheve and Vushtrri, Kosovo	Cattle: 285; sheep: 87; goat: 13	ID Screen^®^ CCHF Double Antigen Multi-species ELISA (IDvet)	Overall: 66/385 (17.1%); Malisheve: 59/239 (24.7%); Vushtrri: 7/146 (4.8%)	Overall: 13.7–21.2%; Malisheve: 19.6–30.5%; Vushtrri: 2.3–9.6%	NA	NA
Karagulov et al., 2022 [110]	Kazakhstan	Bat samples: 1149 total (393 swabs; 349 brain; 407 guano); CCHFV-tested swabs + guano: 800	OM-Screen-CCHF/Q-RT real-time RT-PCR (Syntol, Russia)	NA	NA	0/800	0.0–0.5%
Balinandi et al., 2021 [111]	Uganda	Cattle: 500	In-house CCHFV ELISA + ID Screen^®^ CCHF Double Antigen Multi-species ELISA (IDvet) + IFA + RT-PCR	In-house ELISA: 63/500 (12.6%); IDVet ELISA: 375/500 (75.0%); IFA: 24/37 (64.9%)	In-house ELISA: 10.0–15.8%; IDVet ELISA: 71.0–78.6%; IFA: 48.8–78.2%	0/37 (0.0%)	0.0–9.4%
Blanco-Penedo et al., 2021 [112]	Kenya (Maasai Mara ecosystem, Nanyuki and Ol Pejeta)	Cattle: 148	ID Screen^®^ CCHF Double Antigen Multi-species ELISA (IDvet)	46/148 (31.1%)	24.2–38.9%	NA	NA
Bouaicha et al., 2021 [113]	Southern Tunisia	Dromedary camel: 273	In-house camel CCHFV IgG ELISA + ID Screen^®^ CCHF Double Antigen Multi-species ELISA (IDvet) + CCHFV Mosaic 2 IFA (Euroimmun) + RT-qPCR	245/273 (89.7%)	85.6–92.8%	0/273 (0.0%)	0.0–1.4%
Camp et al., 2021 [114]	Abu Dhabi livestock market, United Arab Emirates	Camel: 90; cattle: 51; goat: 45; sheep: 55	ID Screen^®^ CCHF Double Antigen Multi-species ELISA (IDvet) + RT-qPCR	Camel: 72/90 (80.0%); cattle: 7/51 (13.7%); goat: 1/45 (2.2%); sheep: 4/55 (7.3%)	Camel: 70.6–87.0%; cattle: 6.8–25.7%; goat: 0.4–11.6%; sheep: 2.9–17.3%	Camel: 2/90 (2.2%); cattle: 0/51 (0.0%); goat: 0/45 (0.0%); sheep: 0/55 (0.0%)	Camel: 0.6–7.7%; cattle: 0.0–7.0%; goat: 0.0–7.9%; sheep: 0.0–6.5%
Esmaeel et al., 2021 [115]	Mosul, Iraq	Cattle: 184	CCHFV indirect ELISA	40/184 (21.7%)	16.4–28.2%	NA	NA
Ghasemian et al., 2021 [116]	Kohgiluyeh and Boyer-Ahmad Province, Iran	Sheep: 191; goat: 310	In-house CCHFV IgG ELISA	Overall: 4/501 (0.8%); sheep: 4/191 (2.1%); goat: 0/310 (0.0%)	Overall: 0.3–2.0%; sheep: 0.8–5.3%; goat: 0.0–1.2%	NA	NA
Kajihara et al., 2021 [117]	Zambia	Cattle: 1047; RT-PCR subset: 526	CCHFV NP IgG IFA + nested RT-PCR (S segment)	88/1047 (8.4%)	6.9–10.2%	0/526 (0.0%)	0.0–0.7%
Khalafalla et al., 2021 [118]	United Arab Emirates	Dromedary camel: 238	Semi-nested RT-PCR (L segment) + WGS	NA	NA	16/238 (6.7%)	4.2–10.6%
Khamassi Khbou et al., 2021 [119]	Tunisia	Sheep: 270	ID Screen^®^ CCHF Double Antigen Multi-species ELISA (IDvet) + VNT	1/270 (0.4%); two additional borderline ELISA samples were VNT-negative	0.1–2.1%	NA	NA
Msimang et al., 2021 [120]	South Africa	Cattle: 700	In-house CCHFV IgG sandwich ELISA	515/700 (73.6%); herds: 92/93 (98.9%)	70.2–76.7%; herd-level: 94.2–99.8%	NA	NA
Obaidat et al., 2021 [121]	Jordan	Cattle: 152; sheep: 509; goat: 327	CCHFV IgG indirect ELISA (CDC)	Cattle: 4/152 (2.6%); sheep: 85/509 (16.7%); goat: 48/327 (14.7%)	Cattle: 1.0–6.6%; sheep: 13.7–20.2%; goat: 11.3–18.9%;	NA	NA
Obanda et al., 2021 [122]	Kenya	Buffalo: 191; cattle: 139	ID Screen^®^ CCHF Double Antigen Multi-species ELISA (IDvet)	Buffalo: 144/191 (75.4%); cattle: 39/139 (28.1%)	Buffalo: 68.8–81.0%; cattle: 21.3–36.0%	NA	NA
Phonera et al., 2021 [123]	Malawi	Cattle: 416	ID Screen^®^ CCHF Double Antigen Multi-species ELISA (IDvet)	195/416 (46.9%)	42.1–51.7%	NA	NA
Schulz et al., 2021 [124]	Mauritania	Cattle: 201; sheep: 247; goat: 233; camel: 247	In-house CCHFV IgG ELISAs + CCHFV IgG ELISA (Vector-Best)/CCHFV IFA; camel-specific in-house IgG ELISA	Overall: 411/928 (44.3%); cattle: 138/201 (68.7%); sheep: 39/247 (15.8%); goat: 35/233 (15.0%); camel: 199/247 (80.6%)	Overall: 41.1–47.5%; cattle: 61.9–74.7%; sheep: 11.8–20.9%; goat: 11.0–20.2%; camel: 75.2–85.0%	NA	NA
Trivedi et al., 2021 [125]	Bhavnagar District, India	Livestock: 191	CCHFV cattle IgG ELISA + RT-qPCR	30/191 (15.7%)	11.2–21.5%	NR	—
Vaziri et al., 2021 [126]	Sistan and Baluchestan, Iran	Dromedary camel: 99	In-house CCHFV IgG ELISA	33/99 (33.3%); 12 sera were borderline	24.8–43.1%	NA	NA
Zouaghi et al., 2021 [127]	Tunisia	Cattle: 388; sheep: 325; goat: 166	ID Screen^®^ CCHF Double Antigen Multi-species ELISA (IDvet) + CCHFV IFA	Overall: 76/879 (8.6%); cattle: 43/388 (11.1%); sheep: 20/325 (6.2%); goat: 13/166 (7.8%)	Overall: 7.0–10.7%; cattle: 8.3–14.6%; sheep: 4.0–9.3%; goat: 4.6–12.9%	NA	NA
Camp et al., 2020 [128]	United Arab Emirates	Dromedary camel: 125	ID Screen^®^ CCHF Double Antigen Multi-species ELISA (IDvet) + RT-qPCR + RT-PCR + Sanger sequencing	84/125 (67.2%)	58.6–74.8%	2/125 (1.6%)	0.4–5.6%
Grech-Angelini et al., 2020 [129]	Corsica, France	Cattle: 1731; sheep: 1124; goat: 1035	ID Screen^®^ CCHF Double Antigen Multi-species ELISA (IDvet) + PPRNT	Overall: 290/3890 (7.5%); cattle: 230/1731 (13.3%); sheep: 28/1124 (2.5%); goat: 32/1035 (3.1%). Among 35 samples selected for neutralization, 23 had neutralizing antibodies at 1:40 and 10 at 1:80.	Overall: 6.7–8.3%; cattle: 11.8–15.0%; sheep: 1.7–3.6%; goat: 2.2–4.3%; PPRNT 1:40 among selected: 49.2–79.2%	NA	NA
Kar et al., 2020 [130]	East Thrace, Türkiye	*T. hermanni*: 2; *T. graeca*: 19	Nested PCR + sequencing	NA	NA	RNA: 2/21 (9.5%)	2.7–28.9%
Kasi et al., 2020 [131]	Balochistan, Pakistan	Sheep: 800; goat: 800	In-house CCHFV IgG ELISA + CCHFV IgG ELISA (Vector-Best) + CCHFV IgG IFA (Euroimmun) + RT-qPCR (S segment)	Sheep: 149/800 (18.6%); goat: 37/800 (4.6%)	Sheep: 16.1–21.5%; goat: 3.4–6.3%	Sheep serum pools: 8/160 (5.0%); goat serum pools: 0/160 (0.0%)	Sheep pools: 2.6–9.6%; goat pools: 0.0–2.3%
Li et al., 2020 [132]	Inner Mongolia, China	Sheep: 30; camel: 82	CCHFV NP immunoblot	Sheep: 2/30 (6.7%); camel: 47/82 (57.3%)	Sheep: 1.8–21.3%; camel: 46.5–67.5%	NA	NA
Mangombi et al., 2020 [133]	Northwestern Senegal	Cattle: 14; sheep: 136; goat: 29; horse: 64; donkey: 29; dog: 11	ID Screen^®^ CCHF Double Antigen Multi-species ELISA (IDvet)	Overall: 92/283 (32.5%); cattle: 8/14 (57.1%); sheep: 30/136 (22.1%); goat: 2/29 (6.9%); horse: 45/64 (70.3%); donkey: 5/29 (17.2%); dog: 2/11 (18.2%)	Overall: 27.3–38.2%; cattle: 32.6–78.6%; sheep: 15.9–29.7%; goat: 1.9–22.0%; horse: 58.2–80.1%; donkey: 7.6–34.5%; dog: 5.1–47.7%	NA	NA
Oluwayelu et al., 2020 [134]	Nigeria	Cattle: 50; sheep: 50; goat: 50	In-house CCHFV NP IgG ELISA + NP IFAT	ELISA — Cattle: 12/50 (24.0%); sheep: 0/50 (0.0%); goat: 1/50 (2.0%). IFAT — Cattle: 14/50 (28.0%); goat: 1/50 (2.0%); sheep not tested by IFAT.	ELISA — Cattle: 14.3–37.4%; sheep: 0.0–7.1%; goat: 0.4–10.5%. IFAT — Cattle: 17.5–41.7%; goat: 0.4–10.5%	NA	NA
Ozan and Ozkul, 2020 [135]	Samsun, Sivas and Tokat, Türkiye	Cattle: 939; sheep: 383; goat: 15	CCHFV IgG ELISA (Vector-Best) + VNT + RT-qPCR (S segment) + RT-PCR + Sanger sequencing	Cattle: 340/939 (36.2%); sheep: 24/383 (6.3%); goat: 1/15 (6.7%)	Cattle: 33.2–39.3%; sheep: 4.2–9.2%; goat: 1.2–29.8%	Overall: 9/1337 (0.7%); cattle: 7/939 (0.7%); sheep: 2/383 (0.5%); goat: 0/15 (0.0%)	Overall: 0.4–1.3%; cattle: 0.4–1.5%; sheep: 0.1–1.9%; goat: 0.0–20.4%
Zohaib et al., 2020 [136]	Pakistan	Buffalo: 311; cattle: 183; sheep: 424; goat: 440; camel: 480	ID Screen^®^ CCHF Double Antigen Multi-species ELISA (IDvet) + CCHFV antigen ELISA (Vector-Best) + RT-PCR + Sanger sequencing	Overall: 666/1838 (36.2%); buffalo: 92/311 (29.6%); cattle: 81/183 (44.3%); sheep: 138/424 (32.5%); goat: 83/440 (18.9%); camel: 272/480 (56.7%)	Overall: 34.1–38.5%; buffalo: 24.8–34.9%; cattle: 37.3–51.5%; sheep: 28.3–37.1%; goat: 15.5–22.8%; camel: 52.2–61.0%	Buffalo: 4/28 (14.3%); cattle: 0/46 (0.0%); goat: 0/24 (0.0%); RT-PCR: 3/4 (75.0%)	Buffalo: 5.7–31.5%; cattle: 0.0–7.7%; goat: 0.0–13.8%; RT-PCR: 30.1–95.4%
Özüpak et al., 2020 [137]	Northern Türkiye	Cattle: 65; sheep: 42; goat: 64	CCHFV IgG ELISA (Vector-Best) + RT-qPCR	Overall: 113/171 (66.1%); cattle: 25/65 (38.5%); sheep: 35/42 (83.3%); goat: 53/64 (82.8%)	Overall: 58.7–72.8%; cattle: 27.6–50.6%; sheep: 69.4–91.7%; goat: 71.8–90.1%	RT-qPCR: 0/171 (0.0%)	0.0–2.2%
Bilgin et al., 2020 [138]	Türkiye	Cattle: 86; sheep: 81	Quantitative real-time RT-PCR	NA	NA	Cattle: 0/86 (0.0%); sheep: 0/81 (0.0%)	Cattle: 0.0–4.3%; sheep: 0.0–4.5%
Okur-Gumusova et al., 2020 [139]	Türkiye	Water buffalo: 272	ID Screen CCHFV Indirect ELISA kit	0/272 (0.0%)	0.0–1.4%	NA	NA
Földes et al., 2019 [140]	Southwest Hungary (Mecsek Mountain region)	*A. flavicollis*: 1439; *A. agrarius*: 42; *A. sylvaticus*: 156; *M. glareolus*: 448	CCHFV NP IgG dot-blot + CCHFV IgG IFA	Overall: 20/2085 (1.0%); *A. flavicollis*: 18/1439 (1.3%); *A. agrarius*: 1/42 (2.4%); *A. sylvaticus*: 0/156 (0.0%); *M. glareolus*: 1/448 (0.2%)	Overall: 0.6–1.5%; *A. flavicollis*: 0.8–2.0%; *A. agrarius*: 0.4–12.3%; *A. sylvaticus*: 0.0–2.4%; *M. glareolus*: 0.0–1.3%	NA	NA
Saghafipour et al., 2019 [141]	North Khorasan Province, Iran	Cattle: 11; sheep: 99; goat: 26	In-house CCHFV IgG ELISA	Overall: 21/136 (15.4%); cattle: 0/11 (0.0%); sheep: 16/99 (16.2%); goat: 5/26 (19.2%)	Overall: 10.3–22.5%; cattle: 0.0–25.9%; sheep: 10.2–24.7%; goat: 8.5–37.9%	NA	NA
Zabashta et al., 2019 [142]	Rostov Province, Russia	*P. pipistrellus*: 21; *P. pygmaeus*: 3; *P. kuhlii*: 6; *N. noctula*: 15; *E. serotinus*: 2	CCHFV antigen ELISA (Vector-Best)	NA	NA	Overall: 4/47 (8.5%); *P. pipistrellus*: 2/21 (9.5%); *P. pygmaeus*: 0/3 (0.0%); *P. kuhlii*: 0/6 (0.0%); *N. noctula*: 2/15 (13.3%); *E. serotinus*: 0/2 (0.0%)	Overall: 3.4–19.9%; *P. pipistrellus*: 2.7–28.9%; *P. pygmaeus*: 0.0–56.2%; *P. kuhlii*: 0.0–39.0%; *N. noctula*: 3.7–37.9%; *E. serotinus*: 0.0–65.8%
Christova et al., 2018 [143]	Bulgaria	Cattle: 843; sheep: 130; goat: 88	In-house CCHFV NP IgG ELISA + CCHFV IgG ELISA (Vector-Best) + CCHFV IFA (Euroimmun)	Overall: 195/1061 (18.4%); cattle: 165/843 (19.6%); sheep: 10/130 (7.7%); goat: 20/88 (22.7%)	Overall: 16.2–20.8%; cattle: 17.0–22.4%; sheep: 4.2–13.6%; goat: 15.2–32.5%	NA	NA
Kadriaj et al., 2018 [144]	Albania	Cattle: 103; sheep: 45; goat: 4	CCHFV IgG ELISA (Vector-Best)	Overall: 102/152 (67.1%); cattle/bovine: 76/103 (73.8%); sheep: 25/45 (55.6%); goat: 1/4 (25.0%)	Overall: 59.3–74.1%; cattle/bovine: 64.5–81.3%; sheep: 41.2–69.1%; goat: 4.6–69.9%	NA	NA
Shabani et al., 2018 [145]	Jahrom, Fars Province, Iran	Cattle: 29; sheep: 63; goat: 148	BDSL CCHFV IgG sandwich ELISA	Overall: 43/240 (17.9%); cattle: 5/29 (17.2%); sheep: 8/63 (12.7%); goat: 30/148 (20.3%)	Overall: 13.6–23.3%; cattle: 7.6–34.5%; sheep: 6.6–23.1%; goat: 14.6–27.5%	NA	NA
De Liberato et al., 2018 [146]	Italy	Sheep: 540	Indirect IgG ELISA using recombinant CCHFV nucleoprotein (S-gene encoded antigen)	0/540 (0.0%)	0.0–0.7%	NA	NA
Maiga et al., 2017 [147]	Mali	Cattle: 1075	In-house CCHFV IgG ELISA + CCHFV IgG ELISA (Vector-Best) + CCHFV IgG IFA (Euroimmun)	706/1075 (65.7%)	62.8–68.5%	NA	NA
Sas et al., 2017 [148]	Mauritania	Cattle: 495	In-house CCHFV IgG ELISA + CCHFV IgG ELISA (Vector-Best) + CCHFV IgG IFA (Euroimmun)	333/495 (67.3%)	63.0–71.3%	NA	NA
Sas et al., 2017 [149]	Katanga Province, Democratic Republic of the Congo	Cattle: 514; goat: 186; sheep: 138	In-house CCHFV IgG ELISA + CCHFV IgG ELISA (Vector-Best) + CCHFV IgG IFA (Euroimmun)	Overall: 13/838 (1.6%); cattle: 2/514 (0.4%); goat: 11/186 (5.9%); sheep: 0/138 (0.0%)	Overall: 0.9–2.6%; cattle: 0.1–1.4%; goat: 3.3–10.3%; sheep: 0.0–2.7%	NA	NA
Schuster et al., 2017 [150]	Central and Western Macedonia, Greece	Cattle: 538; sheep: 81	In-house indirect CCHFV IgG ELISA + CCHFV IgG ELISA (Vector-Best) + CCHFV IFA (Euroimmun)	Overall: 32/619 (5.2%); cattle: 31/538 (5.8%); sheep: 1/81 (1.2%)	Overall: 3.7–7.2%; cattle: 4.1–8.1%; sheep: 0.2–6.7%	NA	NA
Shahhosseini et al., 2017 [151]	Chabahar County, southeast Iran	Goat: 3; cattle: 3; sheep: 1	In-house recombinant CCHFV IgG ELISA	1/7 (14.3%)	2.6–51.3%	NA	NA
Suliman et al., 2017 [152]	Khartoum State, central Sudan	Dromedary camel: 361	In-house CCHFV IgG ELISA	77/361 (21.3%)	17.4–25.8%	NA	NA
Bażanów et al., 2017 [153]	Poland	Cattle: 592	IgG sandwich ELISA	0/592 (0.0%)	0.0–0.6%	NA	NA
Champour et al., 2016 [154]	North, Razavi and South Khorasan Provinces, Iran	Dromedary camel: 170	In-house CCHFV IgG sandwich ELISA	9/170 (5.3%)	2.8–9.8%	NA	NA
Hosseini-Vasoukolaei et al., 2016 [155]	Ghaemshahr County, Mazandaran Province, Iran	Sheep: 84	In-house CCHFV IgG ELISA	4/84 (4.8%)	1.9–11.6%	NA	NA
Mertens et al., 2016 [156]	Bulgaria; Türkiye	Sheep; cattle Türkiye: 497; Bulgaria: 619; historical Bulgaria: 49	In-house CCHFV IgG ELISA + CCHFV IgG ELISA (Vector-Best) + CCHFV IFA (Euroimmun)	Bulgaria: 26%; Türkiye: 57%; historical Burgas sheep: 86%	Bulgaria: 23–30%; Türkiye: 53–62%; historical Burgas sheep: 73–94%	NA	NA
Müller et al., 2016 [157]	Congo; Gabon; Ghana; Germany; Panama	Bats: 1135	CCHFV glycoprotein IFA (Euroimmun) + VLP neutralization + VNT	IIFT-reactive: 114/1135 (10.0%); VLP neutralization: 11/30 (36.7%); VNT: 5/7 (71.4%)	IIFT: 8.4–11.9%; VLP neutralization: 21.9–54.5%; VNT: 35.9–91.8%	NA	NA
Schuster et al., 2016 [7]	Albania; North Macedonia	Albania — Cattle: 241; sheep: 142; goat: 151; North Macedonia — Cattle: 123; sheep: 124; goat: 83	In-house CCHFV IgG ELISA + CCHFV IgG ELISA (Vector-Best) + CCHFV IgG IFA	Albania — Cattle: 17/241 (7.1%); sheep: 65/142 (45.8%); goat: 42/151 (27.8%); overall: 124/534 (23.2%). North Macedonia — Cattle: 20/123 (16.3%); sheep: 93/124 (75.0%); goat: 49/83 (59.0%); overall: 162/330 (49.1%).	Albania — Cattle: 4.5–11.0%; sheep: 37.8–54.0%; goat: 21.3–35.4%; overall: 19.8–27.0%. North Macedonia — Cattle: 10.8–23.8%; sheep: 66.7–81.8%; goat: 48.3–69.0%; overall: 43.7–54.5%.	NA	NA
Sherifi et al., 2016 [158]	Kosovo	Cattle: 172; sheep: 95	CCHFV IgG/IgM IFA	Cattle — IgG: 7/172 (4.1%); IgM: 0/172 (0.0%). Sheep — IgG: 2/95 (2.1%); IgM: 1/95 (1.1%); any antibody: 3/95 (3.2%)	Cattle — IgG: 2.0–8.2%; IgM: 0.0–2.2%. Sheep — IgG: 0.6–7.4%; IgM: 0.2–5.7%; any antibody: 1.1–8.9%	NA	NA
Wangchuk et al., 2016 [159]	Bhutan	Goat: 81; cattle: 92	NIV Pune CCHFV IgG ELISA	Goat: 31/81 (38.2%); cattle: 0/92 (0.0%)	Goat: 28.4–49.2%; cattle: 0.0–4.0%	NA	NA
Ibrahim et al., 2015 [160]	East Darfur State, Sudan	Cattle: 282	In-house indirect CCHFV IgG ELISA	54/282 (19.1%)	15.0–24.1%	NA	NA
Mertens et al., 2015 [161]	North Macedonia	Cattle: 158	In-house CCHFV NP IgG ELISA + CCHFV IgG ELISA (Vector-Best) + CCHFV IFA (Euroimmun)	Overall: 23/158 (14.6%); Northeastern region: 16/20 (80.0%); Skopje: 3/35 (8.6%); Vardar: 4/23 (17.4%); Southeastern: 0/80 (0.0%)	Overall: 9.9–20.9%; Northeastern: 58.4–91.9%; Skopje: 3.0–22.4%; Vardar: 7.0–37.1%; Southeastern: 0.0–4.6%	NA	NA
Mourya et al., 2015 [162]	India (22 states + 1 union territory)	Cattle: 4781; sheep/goat: 855	NIV Pune in-house CCHFV IgG ELISA	Overall: 354/5636 (6.3%); bovines: 260/4781 (5.4%); sheep/goats: 94/855 (11.0%)	Overall: 5.7–6.9%; bovines: 4.8–6.1%; sheep/goats: 9.1–13.3%	NA	NA

Note: Results are shown as positive/tested (%) when raw counts were available. NA = not applicable; NR = not separately reported; — = not calculable.

**Table 3:** Named tick taxa reported as CCHFV-positive in publications from 2015 through 31 August 2026.

Tick Taxon	Positive Reports (n = 106)	% of Eligible Tick Reports	Publications
*Hyalomma marginatum*	27	25.5%	Gasparine et al. 2026 [163]; Dinçer et al. 2025 [164]; Volynkina et al. 2025 [165]; Bernard et al. 2024 [166]; Kiwan et al. 2024 [167]; Goletić et al. 2022 [101]; Tsapko et al. 2022 [168]; Akyildiz et al. 2021 [169]; Kajihara et al. 2021 [117]; Shahid et al. 2021 [170]; Ergünay et al. 2020 [171]; Kasi et al. 2020 [172]; Maghsood et al. 2020 [173]; Gevorgyan et al. 2019 [174]; Negredo et al. 2019 [175]; Sherifi et al. 2018 [176]; Orkun et al. 2017 [177]; Papa et al. (Albania) 2017 [178]; Sedaghat et al. 2017 [179]; Shahhosseini et al. 2017 [151]; Farhadpour et al. 2016 [180]; Horton et al. 2016 [181]; Kulichenko et al. 2016 [182]; Panayotova et al. 2016 [183]; Fakoorziba et al. 2015 [184]; Kayedi et al. 2015 [185]; Sharifinia et al. 2015 [186]
*Rhipicephalus sanguineus* complex	20	18.9%	Rudoler et al. 2025 [51]; Cuadrado-Matías et al. 2024 [187]; Kiwan et al. 2024 [167]; Riabi et al. 2023 [188]; Jafari et al. 2022 [189]; Adjogoua et al. 2021 [190]; Shahid et al. 2021 [170]; Naidenova et al. 2020 [191]; Gevorgyan et al. 2019 [174]; Saghafipour et al. 2019 [141]; Faghihi et al. 2018 [192]; Papa et al. (Greece) 2017 [193]; Sedaghat et al. 2017 [179]; Shahhosseini et al. 2017 [151]; Biglari et al. 2016 [194]; Farhadpour et al. 2016 [180]; Hosseini-Vasoukolaei et al. 2016 [155]; Mehran et al. 2016 [195]; Mohammadian et al. 2016 [196]; Kayedi et al. 2015 [185]
*Hyalomma asiaticum*	16	15.1%	Cleary et al. 2026 [197]; Zhou et al. 2026 [198]; Nurmakhanov et al. 2025 [199]; Ergunay et al. 2024 [200]; Bryant-Genevier et al. 2022 [95]; Jafari et al. 2022 [189]; Sultankulova et al. 2022 [201]; Moming et al. 2018 [202]; Voorhees et al. 2018 [203]; Zhang et al. 2018 [204]; Biglari et al. 2016 [194]; Mehran et al. 2016 [195]; Mohammadian et al. 2016 [196]; Fakoorziba et al. 2015 [184]; Kayedi et al. 2015 [185]; Sharifinia et al. 2015 [186]
*Hyalomma anatolicum*	15	14.2%	Rasheid et al. 2026 [29]; Berdikulov et al. 2025 [205]; Riabi et al. 2023 [188]; Shahid et al. 2021 [170]; Kasi et al. 2020 [172]; Saghafipour et al. 2019 [141]; Faghihi et al. 2018 [192]; Mohamed et al. 2017 [206]; Sedaghat et al. 2017 [179]; Biglari et al. 2016 [194]; Champour et al. 2016 [154]; Farhadpour et al. 2016 [180]; Mohammadian et al. 2016 [196]; Kayedi et al. 2015 [185]; Sharifinia et al. 2015 [186]
*Hyalomma dromedarii*	12	11.3%	Temani et al. 2025 [207]; Bendary et al. 2022 [208]; Camp et al. 2021 [114]; Schulz et al. 2021 [209]; Shahid et al. 2021 [170]; Camp et al. 2020 [128]; Kasi et al. 2020 [172]; Faghihi et al. 2018 [192]; Mohamed et al. 2017 [206]; Sedaghat et al. 2017 [179]; Champour et al. 2016 [154]; Horton et al. 2016 [181]
*ConHyalomma rufipes*	13	12.3%	Sarr et al. 2026 [210]; Khoule et al. 2025 [211]; Sadio et al. 2025 [52]; Addo et al. 2024 [212]; Ngom et al. 2024 [73]; Sene et al. 2024 [76]; Badji et al. 2023 [213]; Bentil et al. 2023 [214]; Bendary et al. 2022 [208]; Mancuso et al. 2022 [215]; Msimang et al. 2021 [120]; Schulz et al. 2021 [209]; Shahid et al. 2021 [170]
*Rhipicephalus bursa*	12	11.3%	Gasparine et al. 2026 [163]; Volynkina et al. 2025 [165]; Kiwan et al. 2024 [167]; Tsapko et al. 2022 [168]; Gevorgyan et al. 2019 [174]; Hosseini et al. 2019 [216]; Sherifi et al. 2018 [176]; Orkun et al. 2017 [177]; Papa et al. (Greece) 2017 [193]; Kulichenko et al. 2016 [182]; Mehran et al. 2016 [195]; Orkun et al. 2016 [217]
*Amblyomma variegatum*	9	8.5%	Addo et al. 2024 [212]; Amoah et al. 2024 [218]; Badji et al. 2023 [213]; Bentil et al. 2023 [214]; Lule et al. 2022 [103]; Adjogoua et al. 2021 [190]; Naidenova et al. 2020 [191]; Akuffo et al. 2016 [219]; Horton et al. 2016 [181]
*Dermacentor marginatus*	7	6.6%	Berdikulov et al. 2025 [205]; Dinçer et al. 2025 [164]; Cuadrado-Matías et al. 2024 [187]; Sánchez-Seco et al. 2022 [220]; Tsapko et al. 2022 [168]; Moraga-Fernández et al. 2021 [221]; Orkun et al. 2017 [177]
*Hyalomma scupense*	7	6.6%	Gasparine et al. 2026 [163]; Berdikulov et al. 2025 [205]; Gazezova et al. 2025 [222]; Nurmakhanov et al. 2025 [199]; Nurmakhanov et al. 2024 [223]; Tsapko et al. 2022 [168]; Kasi et al. 2020 [172]
*Hyalomma impeltatum*	6	5.7%	Sarr et al. 2026 [210]; Khoule et al. 2025 [211]; Ngom et al. 2024 [73]; Badji et al. 2023 [213]; Bouaicha et al. 2021 [113]; Chitimia-Dobler et al. 2019 [224]
*Hyalomma truncatum*	6	5.7%	Daodu et al. 2025 [225]; Badji et al. 2023 [213]; Bentil et al. 2023 [214]; Simo Tchetgna et al. 2023 [91]; Kajihara et al. 2021 [117]; Msimang et al. 2021 [120]
*Ixodes ricinus*	6	5.7%	Volynkina et al. 2025 [165]; Cuadrado-Matías et al. 2024 [187]; Ahrabi et al. 2023 [226]; Sultankulova et al. 2022 [201]; Sánchez-Seco et al. 2022 [220]; Tsapko et al. 2022 [168]
*Rhipicephalus annulatus*	5	4.7%	Sánchez-Seco et al. 2022 [220]; Tsapko et al. 2022 [168]; Naidenova et al. 2020 [191]; Gevorgyan et al. 2019 [174]; Horton et al. 2016 [181]
*Rhipicephalus turanicus*	6	5.7%	Dinçer et al. 2025 [164]; Rudoler et al. 2025 [51]; Bryant-Genevier et al. 2022 [95]; Tsapko et al. 2022 [168]; Orkun et al. 2017 [177]; Fakoorziba et al. 2015 [184]
*Hyalomma excavatum*	5	4.7%	Riabi et al. 2023 [188]; Kasi et al. 2020 [172]; Orkun et al. 2017 [177]; Akuffo et al. 2016 [219]; Horton et al. 2016 [181]
*Hyalomma lusitanicum*	5	4.7%	Sánchez-Mora et al. 2026 [227]; Riabi et al. 2023 [188]; Sánchez-Seco et al. 2022 [220]; Moraga-Fernández et al. 2021 [221]; Negredo et al. 2019 [175]
*Haemaphysalis punctata*	5	4.7%	Gasparine et al. 2026 [163]; Volynkina et al. 2025 [165]; Kiwan et al. 2024 [167]; Tsapko et al. 2022 [168]; Dinçer et al. 2025 [164]
*Rhipicephalus appendiculatus*	4	3.8%	Atim et al. 2023 [81]; Atim et al. 2022 [94]; Lule et al. 2022 [103]; Fakoorziba et al. 2015 [184]
*Hyalomma detritum*	3	2.8%	Jafari et al. 2022 [189]; Moming et al. 2018 [202]; Sharifinia et al. 2015 [186]
*Rhipicephalus decoloratus*	3	2.8%	Badji et al. 2023 [213]; Chiuya et al. 2021 [228]; Naidenova et al. 2020 [191]
*Rhipicephalus evertsi evertsi*	3	2.8%	Sarr et al. 2026 [210]; Badji et al. 2023 [213]; Mhamadi et al. 2022 [106]
*Haemaphysalis parva*	2	1.9%	Dinçer et al. 2025 [164]; Orkun et al. 2017 [177]
*Hyalomma aegyptium*	2	1.9%	Panayotova et al. 2023 [229]; Kar et al. 2020 [130]
*Hyalomma impressum*	2	1.9%	Adjogoua et al. 2021 [190]; Shahid et al. 2021 [170]
*Rhipicephalus geigyi*	2	1.9%	Adjogoua et al. 2021 [190]; Naidenova et al. 2020 [191]
*Rhipicephalus guilhoni*	2	1.9%	Sarr et al. 2026 [210]; Mhamadi et al. 2022 [106]
*Rhipicephalus microplus*	2	1.9%	Adjogoua et al. 2021 [190]; Shahid et al. 2021 [170]

**Table 4:** CCHFV-positive tick studies included in this review (n = 97).

Author	Year	Country/Region	CCHFV Result/Positive Tick Taxa	Diagnostic Method
Cleary et al. [197]	2026	Mongolia	*Hyalomma asiaticum*	Conventional PCR (S segment) + NGS
Fabi et al. [230]	2026	Tunisia	*Hyalomma* spp.	RT-qPCR (S segment) + Sanger sequencing
Gasparine et al. [163]	2026	France	*Hyalomma marginatum*; *Hyalomma scupense*; *Rhipicephalus bursa*; *Rhipicephalus* spp.; *Haemaphysalis punctata*	RT-qPCR (L segment) + nanopore sequencing
Koshemetov et al. [23]	2026	Kazakhstan	Tested ticks included *Hyalomma marginatum*, *Dermacentor marginatus*, *Dermacentor reticulatus*, *Rhipicephalus sanguineus* and *Ixodes ricinus*; CCHFV-positive taxon not separately reported	RT-qPCR
Rasheid et al. [29]	2026	Iraq	*Hyalomma anatolicum*	CCHFV antigen ELISA (VectoCrimean-CHF)
Sarr et al. [210]	2026	Senegal	*Hyalomma rufipes*; *Hyalomma impeltatum*; *Rhipicephalus evertsi evertsi*; *Rhipicephalus guilhoni*; *Rhipicephalus mushamae*	RT-qPCR
Sulaiman et al. [30]	2026	Iraq	Tested ticks included *Hyalomma*, *Haemaphysalis* and *Ixodes*; CCHFV-positive taxon not separately reported	RT-PCR (S segment) + sequencing
Sánchez-Mora et al. [227]	2026	Spain	*Hyalomma lusitanicum*	RT-qPCR (S segment) + nested RT-PCR (S segment) + sequencing
Zhou et al. [198]	2026	China	*Hyalomma asiaticum*; additional ixodid taxa not retained at named-species level	Metatranscriptomic sequencing
Berdikulov et al. [205]	2025	Kazakhstan	*Dermacentor pictus*; *Dermacentor marginatus*; *Hyalomma scupense*; *Hyalomma anatolicum*	RT-qPCR + nested RT-PCR + sequencing
Daodu et al. [225]	2025	Nigeria	*Hyalomma truncatum*	RT-PCR (S segment) + amplicon sequencing
Dinçer et al. [164]	2025	Türkiye	*Hyalomma marginatum; Dermacentor marginatus; Haemaphysalis parva; Rhipicephalus turanicus; Haemaphysalis punctata*	Conventional PCR (nairovirus L segment) + sequencing
Gazezova et al. [222]	2025	Kazakhstan	*Hyalomma scupense*	PCR + CCHFV antigen ELISA
Khoule et al. [211]	2025	Senegal	*Hyalomma rufipes*; *Hyalomma impeltatum*	RT-qPCR
Nurmakhanov et al. [199]	2025	Kazakhstan	*Hyalomma asiaticum*; *Hyalomma scupense*	RT-qPCR
Rudoler et al. [51]	2025	Israel	*Rhipicephalus turanicus; Rhipicephalus sanguineus complex; Hyalomma spp. (species not resolved)*	RT-qPCR (S segment) + RT-PCR (S segment) + Sanger sequencing
Sadio et al. [52]	2025	Senegal	*Hyalomma rufipes*	RT-qPCR
Temani et al. [207]	2025	Algeria	*Hyalomma dromedarii*; *Hyalomma* spp.	RT-qPCR + sequencing
Volynkina et al. [165]	2025	Armenia	*Hyalomma marginatum*; *Haemaphysalis punctata*; *Ixodes ricinus*; *Rhipicephalus bursa*	RT-qPCR + Sanger sequencing
Addo et al. [212]	2024	Ghana	*Hyalomma rufipes*; *Amblyomma variegatum*	RT-qPCR (S segment) + NGS
Amoah et al. [218]	2024	Ghana	*Amblyomma variegatum*	RT-qPCR (S segment)
Bernard et al. [166]	2024	France	*Hyalomma marginatum*	RT-qPCR + RT-PCR + NGS
Cuadrado-Matías et al. [187]	2024	Spain	*Dermacentor marginatus*; *Ixodes ricinus*; *Rhipicephalus sanguineus* complex	Nested RT-PCR (S segment) + sequencing
Ergunay et al. [200]	2024	Mongolia	*Hyalomma asiaticum*	Nanopore metagenomic sequencing
Kiwan et al. [167]	2024	France	*Hyalomma marginatum*; *Rhipicephalus sanguineus* complex; *Rhipicephalus bursa*; *Haemaphysalis punctata*	RT-qPCR (L and S segments) + sequencing
Ngom et al. [73]	2024	Senegal	*Hyalomma rufipes*; *Hyalomma impeltatum*	RT-PCR
Nurmakhanov et al. [223]	2024	Kazakhstan	*Hyalomma scupense*	RT-qPCR + sequencing
Sene et al. [76]	2024	Senegal	*Hyalomma rufipes*	RT-PCR + NGS (Illumina MiSeq)
Ahrabi et al. [226]	2023	Türkiye	*Ixodes ricinus*; positive immature *Ixodes* spp.; *Haemaphysalis* spp.	Nested RT-PCR (S segment) + sequencing
Atim et al. [81]	2023	Uganda	*Rhipicephalus appendiculatus*	Target-enrichment NGS (ArboCap)
Badji et al. [213]	2023	Senegal	*Hyalomma impeltatum*; *Hyalomma rufipes*; *Hyalomma truncatum*; *Amblyomma variegatum*; *Rhipicephalus evertsi evertsi*; *Rhipicephalus decoloratus*	RT-qPCR
Bentil et al. [214]	2023	Ghana	*Hyalomma rufipes*; *Hyalomma truncatum*; *Amblyomma variegatum*; *Rhipicephalus* spp.	RT-qPCR (S segment) + WGS
El et al. [83]	2023	Mauritania	*Hyalomma* spp.	RT-qPCR
Moraga-Fernández et al. [231]	2023	Spain	*Argas* spp.	PCR (S segment) + sequencing
Panayotova et al. [229]	2023	Bulgaria	*Hyalomma aegyptium*	RT-qPCR
Riabi et al. [188]	2023	Iran	*Hyalomma anatolicum; Hyalomma lusitanicum; Hyalomma excavatum; Rhipicephalus sanguineus complex*	RT-PCR
Simo Tchetgna et al. [91]	2023	Cameroon	*Hyalomma truncatum*	RT-PCR (L segment) + sequencing
Atim et al. [94]	2022	Uganda	*Rhipicephalus appendiculatus*	Target-enrichment NGS (ArboCap)
Bendary et al. [208]	2022	Egypt	*Hyalomma dromedarii*; *Hyalomma rufipes*	Nested RT-PCR (S segment) + RT-qPCR + Sanger sequencing
Bryant-Genevier et al. [95]	2022	Kazakhstan	*Rhipicephalus turanicus*; *Hyalomma turanicum*; *Hyalomma asiaticum*	RT-PCR + antigen-capture ELISA
Goletić et al. [101]	2022	Bosnia and Herzegovina	*Hyalomma marginatum*	RT-qPCR
Jafari et al. [189]	2022	Iran	*Rhipicephalus sanguineus* complex; *Hyalomma detritum*; *Hyalomma asiaticum*	RT-PCR (S segment)
Lule et al. [103]	2022	Uganda	*Rhipicephalus appendiculatus*; *Amblyomma variegatum*	CCHFV antigen ELISA (Vector-Best)
Mancuso et al. [215]	2022	Italy	*Hyalomma rufipes*	RT-qPCR (RealStar CCHFV RT-PCR)
Mhamadi et al. [106]	2022	Senegal	*Rhipicephalus evertsi evertsi*; *Rhipicephalus guilhoni*	RT-qPCR
Sultankulova et al. [201]	2022	Kazakhstan	*Hyalomma asiaticum*; *Ixodes ricinus*	RT-qPCR + RT-PCR (S segment) + sequencing
Sánchez-Seco et al. [220]	2022	Spain	*Hyalomma lusitanicum*; *Dermacentor marginatus*; *Ixodes ricinus*; *Rhipicephalus annulatus*	RT-qPCR (S segment) + nested RT-PCR (S segment) + sequencing
Tsapko et al. [168]	2022	Russia	*Hyalomma marginatum*; *Hyalomma scupense*; *Rhipicephalus rossicus*; *Rhipicephalus turanicus*; *Rhipicephalus bursa*; *Rhipicephalus annulatus*; *Dermacentor marginatus*; *Haemaphysalis punctata*; *Ixodes ricinus*	RT-PCR
Adjogoua et al. [190]	2021	Côte d’Ivoire	*Amblyomma variegatum*; *Hyalomma impressum*; *Rhipicephalus sanguineus* complex; *Rhipicephalus geigyi*; *Rhipicephalus microplus*	RT-PCR
Akyildiz et al. [169]	2021	Türkiye	*Hyalomma marginatum*	Nested PCR
Bouaicha et al. [113]	2021	Tunisia	*Hyalomma impeltatum*	RT-qPCR
Camp et al. [114]	2021	United Arab Emirates	*Hyalomma dromedarii*	RT-qPCR + shotgun transcriptomic sequencing
Chiuya et al. [228]	2021	Kenya	*Rhipicephalus decoloratus*; *Rhipicephalus* sp.	RT-PCR-HRM (nairovirus screening) + amplicon sequencing
Kajihara et al. [117]	2021	Zambia	*Hyalomma marginatum*; *Hyalomma truncatum*	Nested RT-PCR (S segment) + Sanger sequencing
Moraga-Fernández et al. [221]	2021	Spain	*Hyalomma lusitanicum*; *Dermacentor marginatus*	Nested RT-PCR (S segment) + sequencing
Msimang et al. [120]	2021	South Africa	*Hyalomma rufipes*; *Hyalomma truncatum*; *Hyalomma* spp.	RT-qPCR
Schulz et al. [209]	2021	Mauritania	*Hyalomma dromedarii*; *Hyalomma rufipes*	RT-qPCR (S segment) + sequencing
Shahid et al. [170]	2021	Pakistan	*Hyalomma anatolicum*; *Hyalomma marginatum*; *Hyalomma rufipes*; *Hyalomma impressum*; *Hyalomma dromedarii*; *Rhipicephalus microplus*; *Rhipicephalus sanguineus* complex	CCHFV antigen ELISA + one-step RT-PCR + nested PCR (S segment) + sequencing
Zakham et al. [232]	2021	Saudi Arabia	*Hyalomma spp. (positive pool not attributable to a named species)*	Metagenomic sequencing
Camp et al. [128]	2020	United Arab Emirates	*Hyalomma dromedarii*	RT-PCR (S and M segments) + Sanger sequencing
Ergünay et al. [171]	2020	Türkiye	*Hyalomma marginatum (CCHFV Europe 1); other reported taxa associated with Aigai/Europe 2 excluded*	Generic orthonairovirus PCR (L segment) + Sanger sequencing
Kar et al. [130]	2020	Türkiye	*Hyalomma aegyptium*	Nested RT-PCR (S segment) + Sanger sequencing
Kasi et al. [172]	2020	Pakistan	*Hyalomma marginatum*; *Hyalomma excavatum*; *Hyalomma dromedarii*; *Hyalomma anatolicum*; *Hyalomma scupense*	RT-qPCR (S and L segments) + sequencing
Maghsood et al. [173]	2020	Iran	*Hyalomma marginatum*	One-step conventional RT-PCR (S segment)
Naidenova et al. [191]	2020	Guinea	*Amblyomma variegatum*; *Rhipicephalus annulatus*; *Rhipicephalus geigyi*; *Rhipicephalus decoloratus*; *Rhipicephalus sanguineus* complex	CCHFV antigen ELISA + RT-qPCR
Sahay et al. [233]	2020	India	CCHFV-positive *Hyalomma* ticks	RT-qPCR + NGS
Özüpak et al. [137]	2020	Türkiye	Tested ticks included *Hyalomma marginatum*, *Hyalomma detritum*, *Rhipicephalus bursa* and *Dermacentor marginatus*; CCHFV-positive taxon not separately reported	RT-qPCR
Chitimia-Dobler et al. [224]	2019	Sudan	*Hyalomma impeltatum*	RT-qPCR + PCR (S segment) + NGS
Gevorgyan et al. [174]	2019	Armenia	*Hyalomma marginatum*; *Rhipicephalus sanguineus* complex; *Rhipicephalus annulatus*; *Rhipicephalus bursa*	VectoCrimea-CHF antigen-capture ELISA (Vector-Best)
Hosseini et al. [216]	2019	Iran	*Rhipicephalus bursa*	Conventional RT-PCR + RT-qPCR (S segment)
Negredo et al. [175]	2019	Spain	*Hyalomma lusitanicum*; *Hyalomma marginatum*	Nested RT-PCR (S segment) + Sanger sequencing
Saghafipour et al. [141]	2019	Iran	*Hyalomma anatolicum*; *Rhipicephalus sanguineus* complex	RT-PCR (S segment)
Faghihi et al. [192]	2018	Iran	*Hyalomma anatolicum*; *Hyalomma dromedarii*; *Rhipicephalus sanguineus* complex	RT-PCR (S segment) + Sanger sequencing
Moming et al. [202]	2018	China	*Hyalomma asiaticum*; *Hyalomma detritum*; *Dermacentor nuttalli*	RT-PCR (S segment) + sequencing
Sherifi et al. [176]	2018	Albania; Kosovo	*Hyalomma marginatum; Rhipicephalus bursa*	RT-qPCR
Voorhees et al. [203]	2018	Mongolia	*Hyalomma asiaticum*	RT-qPCR + nested RT-PCR (S segment) + Sanger sequencing + NGS
Zhang et al. [204]	2018	China	*Hyalomma asiaticum*	Metagenomic sequencing + RT-PCR
Mohamed et al. [206]	2017	Saudi Arabia	*Hyalomma schulzei*; *Hyalomma anatolicum*; *Hyalomma dromedarii*	RT-qPCR (SYBR Green)
Orkun et al. [177]	2017	Türkiye	*Hyalomma marginatum*; *Dermacentor marginatus*; *Rhipicephalus bursa*; *Rhipicephalus turanicus*; *Hyalomma excavatum*; *Haemaphysalis parva*	Nested RT-PCR + sequencing
Papa et al. (Albania) [178]	2017	Albania	*Hyalomma marginatum*	Nested RT-PCR (S segment) + sequencing
Papa et al. (Greece) [193]	2017	Greece	*Rhipicephalus sanguineus* complex; *Rhipicephalus bursa*	Nested RT-PCR (S segment) + RT-qPCR + sequencing
Sedaghat et al. [179]	2017	Iran	*Hyalomma dromedarii*; *Hyalomma anatolicum*; *Hyalomma marginatum*; *Rhipicephalus sanguineus* complex	RT-PCR
Shahhosseini et al. [151]	2017	Iran	*Hyalomma* spp.; *Rhipicephalus* spp.; named positive taxa include *Hyalomma marginatum* and *Rhipicephalus sanguineus* complex	One-step RT-PCR (S segment) + sequencing
Akuffo et al. [219]	2016	Ghana	*Hyalomma excavatum*; *Amblyomma variegatum*	One-step RT-PCR (S segment)
Biglari et al. [194]	2016	Iran	*Hyalomma anatolicum*; *Hyalomma asiaticum*; *Hyalomma* sp.; *Rhipicephalus sanguineus* complex; *Haemaphysalis sulcata*	One-step conventional RT-PCR (S segment) + sequencing
Champour et al. [154]	2016	Iran	*Hyalomma dromedarii*; *Hyalomma anatolicum*	One-step conventional RT-PCR (S segment)
Farhadpour et al. [180]	2016	Iran	*Hyalomma marginatum*; *Hyalomma anatolicum*; *Rhipicephalus sanguineus* complex	RT-PCR
Horton et al. [181]	2016	Djibouti	*Hyalomma marginatum*; *Amblyomma lepidum*; *Amblyomma variegatum*; *Amblyomma cohaerens*; *Hyalomma dromedarii*; *Hyalomma excavatum*; *Rhipicephalus annulatus*; *Dermacentor* spp.; *Rhipicephalus* spp.	RT-qPCR (S segment) + sequencing
Hosseini-Vasoukolaei et al. [155]	2016	Iran	*Rhipicephalus sanguineus* complex	Conventional RT-PCR + RT-qPCR (S segment) + sequencing
Kulichenko et al. [182]	2016	Russia	*Hyalomma marginatum*; *Rhipicephalus bursa*; additional positive ixodid pools not separately attributable	PCR + sequencing (S, M and L segments) + WGS
Mehran et al. [195]	2016	Iran	*Hyalomma asiaticum*; *Rhipicephalus sanguineus* complex; *Rhipicephalus bursa*	RT-PCR (S segment)
Mohammadian et al. [196]	2016	Iran	*Hyalomma anatolicum*; *Hyalomma asiaticum*; *Rhipicephalus sanguineus* complex	RT-PCR (S segment)
Orkun et al. [217]	2016	Türkiye	*Rhipicephalus bursa*	Nested RT-PCR (S segment) + sequencing
Panayotova et al. [183]	2016	Bulgaria	*Hyalomma marginatum*	RT-qPCR + nested RT-PCR (S segment) + sequencing
Fakoorziba et al. [184]	2015	Iran	*Rhipicephalus appendiculatus*; *Hyalomma asiaticum*; *Hyalomma marginatum*; *Rhipicephalus turanicus*	Conventional RT-PCR + RT-qPCR (S segment) + Sanger sequencing
Kayedi et al. [185]	2015	Iran	*Hyalomma marginatum*; *Hyalomma anatolicum*; *Hyalomma asiaticum*; *Rhipicephalus sanguineus* complex	Conventional RT-PCR + RT-qPCR (S segment) + Sanger sequencing
Sharifinia et al. [186]	2015	Iran	*Hyalomma marginatum*; *Hyalomma anatolicum*; *Hyalomma asiaticum*; *Hyalomma detritum*	RT-PCR

**Table 5:** Tick surveillance studies without detected CCHFV in the tested tick material (n = 9).

Author	Year	Tick Species/Taxa Tested	Diagnostic Method
Castro-Scholten et al. [79]	2024	*Rhipicephalus pusillus*; *Rhipicephalus sanguineus* sensu lato; *Haemaphysalis hispanica*; *Hyalomma lusitanicum*; *Ixodes ventalloi*	Multiplex RT-qPCR
Weidinger et al. [93]	2023	*Hyalomma dromedarii*	RealStar CCHFV RT-PCR Kit 1.0
Rollins et al. [234]	2021	*Hyalomma rufipes*; *Amblyomma variegatum*; *Amblyomma* sp.; *Ixodes ventalloi*	RT-PCR
Bilgin et al. [138]	2020	*Boophilus* spp., *Rhipicephalus* spp., *Ixodes* spp., and *Hyalomma* spp.	TaqMan real-time RT-PCR
Shuaib et al. [235]	2020	*Hyalomma* spp.	Real-time RT-PCR
Brătuleanu et al. [236]	2020	*Dermacentor* spp.; *Rhipicephalus* spp.	SYBR Green real-time RT-PCR
Pascucci et al. [237]	2019	*Hyalomma rufipes*; *Hyalomma marginatum, Amblyomma variegatum*	RealStar CCHFV RT-PCR Kit 1.0
Bażanów et al. [153]	2017	*Dermacentor reticulatus*; *Ixodes ricinus*	RealStar CCHFV RT-PCR Kit (Altona Diagnostics)
Palomar et al. [238]	2017	*Hyalomma marginatum*; *Hyalomma lusitanicum*; *Rhipicephalus bursa*	Conventional PCRs, plus real-time qPCR targeting the S segment

**Table 6:** Sensitivity distribution of one explicit positive/tested result per independent cohort/outcome for human CCHFV antibody positivity, direct/composite viral-detection positivity and case fatality.

Outcome	Independent Publications Contributing (n)	Independent Cohort/Outcome Estimates (n)	Median (%)	Range (%)
Antibody positivity	104	118	3.8	0.0–100.0
Viral detection positivity	71	72	13.6	0.0–100.0
Case fatality	54	54	20.0	0.0–100.0

**Table 7:** Human studies included in this review (n = 180 standardised records). Diagnostic methods are listed as reported; antibody results, viral-detection results and case-fatality outcomes are presented separately.

Author, Year	Country/ Region	Human Population	Sample Size	Diagnostic Method	Antibody Result	Antibody 95% CI	Viral Detection	Viral 95% CI	Case Fatality	CFR 95% CI
Ameen et al., 2026 [239]	Iraq	Confirmed cases	1001; outcomes: 996	CCHFV IgM ELISA (Abbexa) + RT-qPCR	All 1001 cases confirmed by real-time RT-PCR and/or ELISA				175/996 (17.6%)	15.3–20.1%
Bilgin et al., 2026 [240]	Türkiye	Adults with arthropod exposure	500	CCHFV IgM ELISA + RT-PCR	CCHF confirmed: 38/500 (7.6%) 95% CI: 5.6–10.3%				NA	NA
Diarra et al., 2026 [241]	Senegal	Livestock contacts	2019	MAGPIX CCHFV NP IgG/IgM assay	217/2019 (10.7%)	9.5–12.2%	NA	NA	NA	NA
Fırtına Topcu et al., 2026 [242]	Türkiye	Children	31	RT-qPCR	NA	NA	13/31 (41.9%)	26.4–59.2%	NA	NA
Ilboudo et al., 2026 [20]	Burkina Faso	Livestock farmers	717	ID Screen® CCHF Double Antigen Multi-species ELISA (IDvet)	22/717 (3.1%)	2.0–4.6%	NA	NA	NA	NA
Jalal et al., 2026 [243]	Iraq (Wasit)	Suspected cases	369	CCHFV IgM ELISA + RT-qPCR	Overall laboratory-confirmed (RT-qPCR and/or CCHFV-specific IgM): 98/369 (26.6%); 95% CI: 22.3–31.3%				11.5%	Not calculable
Karanfil et al., 2026 [244]	Türkiye	Surveillance cases	7776	ELISA and/or PCR	7776 CCHF cases identified through national surveillance				373/7776 (4.8%)	4.3–5.3%
Kasumba et al., 2026 [245]	DRC	Hunter-gatherers	300	In-house CCHFV NP IgG ELISA	12/300 (4.0%)	2.3–6.9%	NA	NA	NA	NA
Khaleel et al., 2026 [246]	Iraq	Suspected patients	3546; confirmed: 955	CCHFV IgM ELISA (Abbexa) + RT-qPCR (S segment)	IgM ELISA: 40/2631 (1.5%) among RT-PCR-negative	1.1–2.1%	915/3546 (25.8%)	24.4–27.3%	153/955 (16.0%)	13.8–18.5%
Kodama et al., 2026 [247]	Iraq	Confirmed cases	273	CCHFV IgM ELISA (Vector-Best) + RT-qPCR	All 273 cases confirmed by IgM ELISA and/or RT-qPCR				33/273 (12.1%)	8.7–16.5%
Lombe et al., 2026 [24]	DRC	Archived patient sera	1239	In-house CCHFV NP ELISA	55/1239 (4.4%)	3.4–5.7%	NA	NA	NA	NA
Marif et al., 2026 [248]	Iraq	Confirmed cases	1089	Laboratory confirmation	1089 confirmed CCHF cases				90/1089 (8.3%)	6.8–10.1%
Nasirian et al., 2026 [249]	Iran (Sistan and Baluchestan)	Suspected cases	433; confirmed: 91; probable: 16	Surveillance case classification	Confirmed: 91/433 (21.0%); probable: 16/433 (3.7%) 95% CI: Confirmed: 17.4–25.1%; probable: 2.3–5.9%				10/91 (11.0%)	6.1–19.1%
Nyakarahuka et al., 2026 [28]	Uganda	Outbreak samples	8	CDC in-house CCHFV IgM-capture ELISA + RT-qPCR (S segment)	IgM: 2/8 (25.0%)	7.1–59.1%	3/8 (37.5%)	13.7–69.4%	NA	NA
Pınarlık et al., 2026 [250]	Türkiye	Healthcare workers	194	CCHFV IgG ELISA (Euroimmun) + PRNT	IgG: 4/194 (2.1%); PRNT: 4/4 (100%)	IgG: 0.8–5.2%; PRNT: 51.0–100.0%	NA	NA	NA	NA
Rasheid et al., 2026 [29]	Iraq	Confirmed cases	150	CCHFV IgG ELISA (Vector-Best) + RT-PCR	All 150 cases confirmed by ELISA and/or RT-PCR				18/150 (12.0%)	7.7–18.2%
Ricks et al., 2026 [251]	Ghana	Ranchers; abattoir workers	300	MAGPIX CCHFV IgG assay + microneutralization	21/300 (7.0%); showed no clear CCHFV neutralization trend	4.6–10.5%	NA	NA	NA	NA
Schafer et al., 2026 [252]	Liberia	Community participants	456	MAGPIX CCHFV NP IgG assay	108/456 (23.7%)	20.0–27.8%	NA	NA	NA	NA
Umair et al., 2026 [253]	Pakistan	Suspected cases	151	RT-qPCR + shotgun metagenomic WGS	NA	NA	23/151 (15.2%)	10.4–21.8%	NA	NA
Abdulrahman et al., 2025 [254]	Iraq	Suspected cases	2186	RT-qPCR	NA	NA	587/2186 (26.9%)	25.0–28.8%	83/587 (14.1%)	11.6–17.2%
Abid et al., 2025 [255]	Pakistan	Suspected cases	2559	RT-qPCR	NA	NA	547/2559 (21.37%)	19.8–23.0%	44/96 (45.8%)	36.2–55.8%
Ammar et al., 2025 [32]	Pakistan	Participants	253	CCHFV NP LIPS + neutralization	LIPS: 5/253 (2.0%); neutralization: 4/253 (1.6%)	LIPS: 0.8–4.5%; neutralization: 0.6–4.0%	NA	NA	NA	NA
Ashraf et al., 2025 [256]	Uganda	Serum samples	230	Metagenomic sequencing	NA	NA	1/230 (0.4%)	0.1–2.4%	NA	NA
Courtney et al., 2025 [257]	Nigeria	Febrile patients	1200; serology: 596	CCHFV serology + TaqMan Array Card multi-pathogen PCR	Serology: 13/596 (2.2%)	1.3–3.7%	4/1200 (0.3%)	0.1–0.9%	NA	NA
Gasparine et al., 2025 [41]	Cameroon	Participants	465	ID Screen® CCHF Double Antigen Multi-species ELISA (IDvet) + RT-qPCR (L segment) + sequencing	9/465 (1.9%)	1.0–3.6%	2/465 (0.43%)	0.1–1.6%	NA	NA
Gazezova et al., 2025 [222]	Kazakhstan	Enrolled case-patients	Case patients: 38 Controls tested: 55; confirmed cases: 15	CCHFV IgG/IgM ELISA (Vector-Best) + CCHFV PCR (AmpliSens)	Case patients: 14/38 (36.8%); controls IgG: 1/55 (1.8%)	Case patients: 23.4–52.7%; controls IgG: 0.3–9.6%	NR	NR	Confirmed cases: 5/15 (33.3%)	15.2–58.3%
Halbrook et al., 2025 [42]	DRC	Agricultural workers	180	In-house CCHFV NP IgG ELISA	23/180 (12.8%)	8.7–18.4%	NA	NA	NA	NA
Hamdard et al., 2025 [258]	Afghanistan	Suspected cases	4445	CCHFV IgM ELISA + RT-qPCR	IgM ELISA and/or RT-qPCR confirmation: 944/4445 (21.2%) 95% CI: 20.1–22.5%				195/944 (20.7%)	18.2–23.4%
Hashim et al., 2025 [259]	Iraq (Thi-Qar)	Confirmed cases	363	CCHFV IgM ELISA (Abbexa) + RT-qPCR	All 363 cases confirmed by IgM ELISA and/or RT-qPCR				69/363 (19.0%)	15.3–23.4%
Hmmod et al., 2025 [260]	Iraq (Thi-Qar)	Confirmed cases	290	Laboratory confirmation	290 confirmed cases				45/290 (15.5%)	11.8–20.1%
Igan & Hanci, 2025 [261]	Türkiye	Suspected samples	1378	RT-PCR	NA	NA	521/1378 (37.8%)	35.3–40.4%	7/521 (1.3%)	0.7–2.7%
Jakimovski et al., 2025 [43]	North Macedonia	Healthcare workers	52	CCHFV IgG ELISA (Vector-Best)	4/52 (7.7%)	3.0–18.2%	NA	NA	NA	NA
Jakimovski et al., 2025 [262]	North Macedonia	Cases	2	CCHFV IgG/IgM ELISA + RT-PCR + sequencing	IgM: 2/2 (100%); IgG: 0/2 (0.0%)	IgM: 34.2–100.0%; IgG: 0.0–65.8%	2/2 (100%)	34.2–100.0%	NA	NA
Khaleel et al., 2025 [263]	Iraq	Confirmed cases	580	CCHFV IgM ELISA + RT-PCR	All 580 cases confirmed by IgM ELISA and/or RT-PCR				NR	—
Kiwan et al., 2025 [264]	France (Corsica)	High-risk participants; residual sera	High risk: 201; residual sera: 2514	ID Screen® CCHF Double Antigen Multi-species ELISA (IDvet) + neutralization	Overall: 3/2715 (0.11%); high risk: 1/201 (0.5%); residual sera: 2/2514 (0.1%); neutralization: 1/3 (33.3%)	Overall: 0.04–0.32%; high risk: 0.09–2.76%; residual: 0.02–0.29%; neutralization: 6.1–79.2%	NA	NA	NA	NA
Lorenzo Juanes et al., 2025 [265]	Spain	African migrants	485	CCHFV IgG/IgM ELISA + RT-PCR	IgG: 18/485 (3.7%); IgM: 9/485 (1.9%)	IgG: 2.4–5.8%; IgM: 1.0–3.5%	0/485 (0.0%)	0.0–0.8%	NA	NA
Lorenzo Juanes et al., 2025 [266]	Spain	Residents in an endemic area	658	CCHFV IgG/IgM ELISA	IgG: 4/658 (0.6%)	0.2–1.6%	NA	NA	NA	NA
Machuetum et al., 2025 [267]	Cameroon	Animal-exposed workers	790 (ELISA); 100 (neutralization subset)	In-house CCHFV IgG ELISA; VSVΔG*CCHFV-GPc pseudotype neutralization assay	IgG: 36/790 (4.6%); neutralization: 0/100 (0.0%)	IgG: 3.3–6.2%; neutralization: 0.0–3.7%	NA	NA	NA	NA
Mihalakakos et al., 2025 [268]	Uganda	Community participants	1199	ID Screen® CCHF Double Antigen Multi-species ELISA (IDvet) + in-house CCHFV IgG ELISA	51/1199 (4.3%)	3.2–5.5%	NA	NA	NA	NA
Mohammed et al., 2025 [47]	Iraq	Cattle owners	124	ID Screen® CCHF Double Antigen Multi-species ELISA (IDvet)	6/124 (4.8%)	2.2–10.2%	NA	NA	NA	NA
Ngoc et al., 2025 [269]	Bulgaria	Confirmed cases	24	CCHFV IgG/IgM ELISA (Vector-Best/Euroimmun) + RT-qPCR + WGS	IgM: 23/24 (95.8%); IgG: 1/24 (4.2%)	IgM: 79.8–99.3%; IgG: 0.7–20.2%	7/24 (29.2%)	14.9–49.2%	2/24 (8.3%)	2.3–25.8%
Olk et al., 2025 [270]	Benin	Febrile patients	650	CCHFV IgG ELISA (Euroimmun) + Panadea ELISA + CCHFV IFA + RT-PCR	IgG: 40/650 (6.2%); Panadea: 5/92 (5.4%); IFA: 2/650 (0.3%)	IgG: 4.6–8.3%; Panadea: 2.3–12.1%; IFA: 0.1–1.1%	0/650 (0.0%)	0.0–0.6%	NA	NA
Sadio et al., 2025 [52]	Senegal	Participants	903	IPD in-house CCHFV IgG/IgM ELISA + RT-PCR	IgG: 7/903 (0.8%); IgM: 0/903 (0.0%)	IgG: 0.4–1.6%; IgM: 0.0–0.4%	0/903 (0.0%)	0.0–0.4%	NA	NA
Sanou et al., 2025 [271]	Burkina Faso	Hepatitis B/C patients	1808	CCHFV IgG ELISA (Abbexa)	20/1808 (1.1%)	0.7–1.7%	NA	NA	NA	NA
Sene et al., 2025 [272]	Senegal	Contacts; confirmed cases	Contacts: 225; confirmed cases: 3	IPD in-house CCHFV IgG/IgM ELISA + RT-PCR + sequencing	Contacts—IgG: 10/225 (4.4%); IgM: 0/225 (0.0%)	IgG: 2.4–8.0%; IgM: 0.0–1.7%	Contacts: 0/225 (0.0%)	0.0–1.7%	Confirmed cases: 2/3 (66.7%)	20.8–93.9%
Shao et al., 2025 [273]	Tanzania	Febrile patients	735	Luminex CCHFV NP IgG assay + IFA + VNT	Luminex: 23/735 (3.1%); IFA: 13/735 (1.8%); VNT: 3/23 (13.0%)	Luminex: 2.1–4.7%; IFA: 1.0–3.0%; VNT: 4.5–32.1%	NA	NA	NA	NA
Sheek-Hussein et al., 2025 [55]	United Arab Emirates	Abattoir workers	86	CCHFV ELISA (Abbexa) + RT-qPCR	25/86 (29.1%)	20.5–39.4%	0/86 (0.0%)	0.0–4.3%	NA	NA
Zia et al., 2025 [274]	Pakistan	Suspected cases	109	RT-qPCR	NA	NA	15/109 (13.8%)	8.5–21.5%	4/15 (26.7%)	10.9–52.0%
Şahan et al., 2025 [275]	Türkiye	Serum samples	27,269	Laboratory confirmation (assay NR)	11,811/27,269 (43.3%)	42.7–43.9%	NA	NA	552/11,811 (4.7%)	4.3–5.1%
Abuova et al., 2024 [276]	Kazakhstan (Turkestan Region)	Rural residents	4259	CCHFV IgG/IgM ELISA (Vector-Best)	102/4259 (2.4%)	2.0–2.9%	NA	NA	NA	NA
Al Salihi et al., 2024 [277]	Iraq	Suspected cases	212	RT-PCR	NA	NA	97/212 (45.8%)	39.2–52.5%	13/97 (13.4%)	8.0–21.6%
Al-Khafaji et al., 2024 [278]	Iraq (Dhi Qar Province)	Enrolled participants	621	RT-PCR	NA	NA	162/621 (26.1%)	22.8–29.7%	48/621 (7.7%)	5.9–10.1%
Arslan & Comoglu, 2024 [279]	Türkiye (Amasya Province)	Confirmed cases	88	RT-qPCR	NA	NA	88 patients positive	NA	3/88 (3.4%)	1.2–9.5%
Ayari et al., 2024 [280]	Tunisia	Blood donors	377	CCHFV Mosaic 2 IgG/IgM IFA (Euroimmun) + RT-PCR	IgG: 1/377 (0.3%); IgM: 0/1 (0.0%)	IgG: 0.0–1.5%; IgM: 0.0–79.3%	0/1 (0.0%)	0.0–79.3%	NA	NA
Baghdadi et al., 2024 [281]	Iraq	National- surveillance confirmed cases	986	CCHFV IgM ELISA (Abbexa) + RT-qPCR	All 986 cases laboratory-confirmed by IgM ELISA and/or RT-qPCR				166/986 (16.8%)	14.6–19.3%
Balinandi et al., 2024 [282]	Uganda	VHF-suspected specimens	13	RT-qPCR (S segment) + sequencing	NA	NA	13 positive cases	NA	7/13 (53.8%)	29.1–76.8%
Bohou Kombila et al., 2024 [283]	Gabon	Rural residents	3081	ID Screen® CCHF Double Antigen Multi-species ELISA (IDvet) + Blackbox CCHFV IgG ELISA + Luminex/MMIA	ID Screen: 15/3081 (0.5%); Blackbox IgG: 7/15 (46.7%); Luminex/MMIA: 4/15 (26.7%)	ID Screen: 0.3–0.8%; Blackbox: 24.8–69.9%; Luminex/MMIA: 10.9–52.0%	NA	NA	NA	NA
Chen et al., 2024 [284]	Pakistan (Faisalabad)	Healthy participants	725	CCHFV NP LIPS + microneutralization	LIPS: 55/725 (7.6%); MNT: 19/725 (2.6%)	LIPS: 5.9–9.7%; MNT: 1.7–4.1%	NA	NA	NA	NA
Daodu et al., 2024 [285]	Nigeria	Herdsmen; febrile patients	Herdsmen: 91; febrile patients: 646	In-house CCHFV NP IgG ELISA + RT-PCR (S segment)	Herdsmen: 84/91 (92.3%); febrile patients: 46/646 (7.1%)	Herdsmen: 85.0–96.2%; febrile patients: 5.4–9.4%	0/555 (0.0%)	0.0–0.7%	NA	NA
Fazlalipour et al., 2024 [286]	Iran	Healthcare workers	96	CCHFV IgM-capture ELISA (MAC-ELISA) + RT-PCR	IgM: 3/96 (3.1%)	1.1–8.8%	11/96 (11.5%)	6.5–19.4%	2/12 (16.7%)	4.7–44.8%
Hanif et al., 2024 [287]	Pakistan	Healthcare workers	13	PCR	NA	NA	9/13 (69.2%)	42.4–87.3%	2/13 (15.4%)	4.3–42.2%
Hughes et al., 2024 [70]	Tanzania	Community participants	351	ID Screen® CCHF Double Antigen Multi-species ELISA (IDvet)	53/351 (15.1%)	11.7–19.2%	NA	NA	NA	NA
Khaleel et al., 2024 [288]	Iraq	Suspected cases	61	RT-qPCR	NA	NA	18/61 (29.5%)	19.6–41.9%	8/18 (44.4%)	24.6–66.3%
Malonga et al., 2024 [289]	Republic of Congo; Mali	People living with HIV; blood donors	HIV: 352; blood donors: 229	ID Screen® CCHF Double Antigen Multi-species ELISA (IDvet)	HIV: 2/352 (0.6%); blood donors: 4/229 (1.7%)	HIV: 0.2–2.0%; blood donors: 0.7–4.4%	NA	NA	NA	NA
Mukhaye et al., 2024 [72]	Kenya (Isiolo County)	Community participants	580	ID Screen® CCHF Double Antigen Multi-species ELISA (IDvet) + RT-qPCR (S segment)	42/580 (7.2%)	5.4–9.6%	0/16 (0.0%)	0.0–19.4%	NA	NA
Mohammed et al., 2024 [290]	Iraq (Nineveh Province)	Butchers; veterinarians	Butchers: 120; veterinarians: 83	CCHFV antibody ELISA	Butchers: 9/120 (7.5%); veterinarians: 6/83 (7.2%)	Butchers: 4.0–13.6%; veterinarians: 3.4–14.9%	NA	NA	NA	NA
Sene et al., 2024 [76]	Senegal	Cases	2	In-house CCHFV IgM ELISA + RT-PCR + sequencing	Case 1: IgM positive; Case 2: initial IgM negative, follow-up IgM positive	NA	Case 1: RT-PCR negative; Case 2: RT-PCR positive	NA	0/2 (0.0%)	0.0–65.8%
Shaker et al., 2024 [291]	Iraq	Suspected cases	206	CCHFV IgG/IgM ELISA + RT-qPCR	17/206 (8.3%) by ELISA and/or RT-PCR 95% CI: 5.2–12.8%				9/17 (52.9%)	31.0–73.8%
Simo et al., 2024 [77]	Cameroon	Cattle herders; febrile patients	Herders: 28; febrile patients: 60	ID Screen® CCHF Double Antigen Multi-species ELISA (IDvet)	Herders: 5/28 (17.9%); febrile patients: 5/60 (8.3%)	Herders: 7.9–35.6%; febrile patients: 3.6–18.1%	NA	NA	NA	NA
Syed et al., 2024 [292]	Pakistan (Sindh Province)	Confirmed cases	81	RT-PCR	NA	NA	All 81 cases positive	NA	36/81 (44.4%)	34.1–55.3%
Umair et al., 2024 [293]	Pakistan	Suspected cases	795	RT-qPCR + WGS	NA	NA	75/795 (9.4%)	7.6–11.7%	12/34 (35.3%)	21.5–52.1%
Zia et al., 2024 [294]	Pakistan (Khyber Pakhtunkhwa)	Suspected cases	150	RT-qPCR	NA	NA	40/150 (26.7%)	20.2–34.3%	NA	NA
Rusanganwa et al., 2024 [295]	Rwanda	Febrile patients	2294 patients; 230 pools	Conventional RT-PCR	NA	NA	0/230 pools (0%)	0.0–1.6%	NA	NA
Doğan et al., 2023 [296]	Türkiye (Bayburt)	Confirmed cases	100	CCHFV IgM ELISA + RT-PCR	IgM: 5/100 (5.0%)	2.2–11.2%	95/100 (95.0%)	88.8–97.8%	1/100 (1.0%)	0.2–5.4%
El Ghassem et al., 2023 [83]	Mauritania	Slaughterhouse workers; animal herders	Slaughterhouse workers: 141; animal herders: 122	ID Screen® CCHF Double Antigen Multi-species ELISA (IDvet) + RT-qPCR	31/263 (11.8%)	8.4–16.2%	2/263 (0.7%)	0.2–2.7%	NA	NA
Jakimovski et al., 2023 [297]	North Macedonia	Confirmed cases	2	CCHFV Mosaic 2 IgM IFA (Euroimmun) + RT-qPCR	IgM positive; case 2 serology NR	—	2/2 (100%)	34.2–100.0%	1/2 (50.0%)	9.5–90.5%
Latinne et al., 2023 [298]	Viet Nam	Participants	30	MAGPIX CCHFV IgG/IgM assay	IgG: 1/30 (3.3%); IgM: 0/30 (0.0%)	IgG: 0.6–16.7%; IgM: 0.0–11.4%	NA	NA	NA	NA
Lorenzo Juanes et al., 2023 [299]	Spain	Confirmed cases	10	CCHFV confirmation + S-segment sequencing	All 10 cases confirmed; S-segment sequences obtained for 8/10 cases				3/10 (30.0%)	10.8–60.3%
Mostafa et al., 2023 [86]	Iraq (Zakho/Duhok)	Slaughterhouse butchers	61	ID Screen® CCHF Double Antigen Multi-species ELISA (IDvet)	2/61 (3.3%)	0.9–11.2%	NA	NA	NA	NA
Oludele et al., 2023 [300]	Mozambique	Pastoralists	218	CCHFV IgG/IgM ELISA (Vector-Best)	IgM: 9/218 (4.1%); IgG: 2/218 (0.9%)	IgM: 2.2–7.7%; IgG: 0.3–3.3%	NA	NA	NA	NA
Omoga et al., 2023 [88]	Kenya	Febrile patients	493	CCHFV IgG/IgM ELISA (Vector-Best) + RT-PCR (S segment) + sequencing	IgG: 29/493 (5.9%); IgM: 9/29 (31.0%)	IgG: 4.1–8.3%; IgM: 17.3–49.2%	3/493 (0.6%)	0.2–1.8%	NA	NA
Sankhe et al., 2023 [301]	Senegal	Participants	1421	In-house CCHFV IgG indirect ELISA	10/1421 (0.7%)	0.4–1.3%	NA	NA	NA	NA
Umair et al., 2023 [302]	Pakistan	Suspected cases	46	RT-qPCR + WGS	NA	NA	6/46 (13.0%)	6.1–25.7%	3/6 (50.0%)	18.8–81.2%
Öner et al., 2023 [303]	Türkiye	Confirmed cases	38	CCHFV IgM ELISA + RT-qPCR	15/38 (39.5%)	25.6–55.3%	23/38 (60.5%)	44.7–74.4%	0/38 (0.0%)	0.0–9.2%
Atim et al., 2022 [94]	Uganda (Arua and Nakaseke districts)	Livestock farmers; non-livestock farmers	Livestock farmers: 386; non-livestock farmers: 414	CCHFV IgG ELISA (Vector-Best)	221/800 (27.6%)	24.6–30.8%	NA	NA	NA	NA
Boushab et al., 2022 [304]	Mauritania	Suspected cases	88	CCHFV IgM ELISA + RT-PCR	NR	—	7/88 (8.0%)	3.9–15.5%	2/7 (28.6%)	8.2–64.1%
Khan et al., 2022 [305]	Pakistan (Peshawar)	Confirmed cases	10	RT-PCR	NA	NA	10 confirmed cases	NA	0/10 (0.0%)	0.0–27.8%
Lule et al., 2022 [103]	Uganda	Abattoir workers	478	CCHFV IgG/IgM ELISA (Vector-Best)	IgM: 14/478 (2.9%); IgG: 37/478 (7.7%); both: 2/478 (0.4%); any antibody: 49/478 (10.3%)	IgM: 1.8–4.9%; IgG: 5.7–10.5%; both: 0.1–1.5%; any antibody: 7.8–13.3%	NA	NA	NA	NA
Mhamadi et al., 2022 [106]	Senegal (Agnam, Matam Region)	Febrile patients	364	In-house CCHFV IgM immunocapture ELISA + in-house CCHFV Gn IgG ELISA + RT-qPCR	IgM: 1/364 (0.3%); IgG: 37/364 (10.2%)	IgM: 0.0–1.5%; IgG: 7.5–13.7%	0/364 (0.0%)	0.0–1.0%	NA	NA
Nyakarahuka et al., 2022 [306]	Uganda (Kyangwali Refugee Settlement)	Confirmed cases	2	CDC in-house CCHFV IgG/IgM ELISA + RT-qPCR + NGS	IgM: 2/2 (100%); IgG: 2/2 (100%)	IgM: 34.2–100.0%; IgG: 34.2–100.0%	2/2 (100%)	34.2–100.0%	0/2 (0.0%)	0.0–65.8%
Sağmak Tartar et al., 2022 [307]	Türkiye (Elazığ)	Confirmed cases	12	CCHFV IgM ELISA and/or RT-PCR	Laboratory-confirmed: 12 cases by IgM ELISA and/or RT-PCR				NR	—
Frías et al., 2022 [308]	Spain	Healthy blood donors	1384	IDScreen® CCHF Double Antigen Multispecies ELISA (IDVet)	0/1384 (0.0%)	0.0–0.3%	NA	NA	NA	NA
Arteaga et al., 2021 [309]	Spain	Febrile patients	133	CCHFV IgG/IgM ELISA (Vector-Best) + in-house ELISA + CCHFV IFA (Euroimmun) + RT-qPCR	IgG: 3/133 (2.3%); IgM: 1/133 (0.8%)	IgG: 0.8–6.4%; IgM: 0.1–4.1%	1/133 (0.8%)	0.1–4.1%	NA	NA
Balinandi et al., 2021 [310]	Uganda	Suspected VHF cases	1261	CCHFV IgM ELISA + RT-qPCR + NGS	NR	—	32/1261 (2.5%)	1.8–3.6%	10/32 (31.3%)	18.0–48.6%
Evans et al., 2021 [311]	Myanmar	Healthy participants from elephant-logging communities	102	MAGPIX CCHFV NP IgG assay + PCR	IgG: 10/102 (9.8%)	5.4–17.1%	0/102 (0.0%)	0.0–3.6%	NA	NA
Gürbüz et al., 2021 [312]	Türkiye (Gümüşhane)	Suspected cases	362	CCHFV IgM ELISA + PCR	242/362 (66.9%) by IgM ELISA and/or PCR 95% CI: 61.8–71.5%				8/362 (2.2%)	1–4.3%;
Habibzadeh et al., 2021 [313]	Iran (northwest)	Suspected cases	51	CCHFV IgM ELISA + RT-PCR	IgM: 1/10 (10.0%)	1.8–40.4%	10/51 (19.6%)	11.0–32.5%	1/10 (10.0%)	1.8–40.4%
Hamidi et al., 2021 [314]	Türkiye	Suspected cases	125; confirmed: 38	CCHFV IgM assay + RT-qPCR	IgM: 3/125 (2.4%); confirmed: 3/38 (7.9%)	Overall: 0.8–6.8%; confirmed: 2.7–20.8%	35/125 (28.0%)	20.9–36.4%	2/38 (5.3%)	1.5–17.3%
Magyar et al., 2021 [315]	Hungary	Blood donors	2700	In-house CCHFV IgG IFA + recombinant CCHFV rNP IgG ELISA (Vector-Best)	IFA-reactive: 12/2700 (0.44%); IFA + ELISA-confirmed seropositive: 10/2700 (0.37%)	IFA: 0.25–0.78%; confirmed: 0.20–0.68%	NA	NA	NA	NA
Mirembe et al., 2021 [316]	Uganda	Suspected cases	409	RT-PCR	NA	NA	14/409 (3.4%)	2.0–5.7%	5/14 (35.7%)	16.3–61.2%
Msimang et al., 2021 [120]	South Africa	Farm/wildlife workers; veterinary personnel; abattoir workers	Farm/wildlife workers: 541; veterinary personnel: 117; abattoir workers: 382	In-house CCHFV IgG sandwich ELISA	Farm/wildlife workers: 21/541 (3.9%); veterinary personnel: 0/117 (0.0%); abattoir workers: 0/382 (0.0%)	Farm/wildlife: 2.6–5.9%; veterinary: 0.0–3.2%; abattoir: 0.0–1.0%	NA	NA	NA	NA
Orhan & Gülsoy, 2021 [317]	Türkiye (Oltu, Erzurum)	Confirmed cases	57	RT-PCR	NA	NA	57 cases positive	—	NR	—
Qaderi et al., 2021 [318]	Afghanistan	Suspected/admitted patients	231	CCHFV IgG/IgM ELISA	51/231 (22.1%)	17.2–27.9%	NA	NA	11/51 (21.6%)	12.5–34.6%
Rugarabamu et al., 2021 [319]	Tanzania	Household/health-facility participants	500	CCHFV IgG/IgM ELISA (MyBioSource)	IgG: 6/500 (1.2%); IgM: 4/500 (0.8%);	IgG: 0.6–2.6%; IgM: 0.3–2.0%;	NA	NA	NA	NA
Trivedi et al., 2021 [125]	India (Bhavnagar District, Gujarat)	Confirmed cases	11	ELISA + PCR	11 positive cases by ELISA and/or PCR				6/11 (54.5%)	28.0–78.7%
Çitil et al., 2021 [320]	Türkiye (Tokat)	Adults aged ≥20 years	2319	CCHFV IgG ELISA	130/2319 (5.6%)	4.7–6.6%	NA	NA	NA	NA
Aydin et al., 2020 [321]	Türkiye (Erzurum)	Animal breeders; slaughterhouse workers; veterinarians; blood donors	Animal breeders: 32; slaughterhouse workers: 25; veterinarians: 15; blood donors: 19	CCHFV IgG ELISA (Vector-Best)	Direct-contact group: 5/72 (6.9%); breeders: 4/32 (12.5%); slaughterhouse workers: 1/25 (4.0%); veterinarians: 0/15 (0.0%); donors: 0/19 (0.0%)	Direct-contact: 3.0–15.2%; breeders: 5.0–28.1%; slaughterhouse: 0.7–19.5%; veterinarians: 0.0–20.4%; donors: 0.0–16.8%	NA	NA	NA	NA
Boushab et al., 2020 [322]	Mauritania	Cases	2	CCHFV IgM ELISA + RT-qPCR	IgM: 2/2 (100%)	34.2–100.0%	2/2 (100%)	34.2–100.0%	NR	—
Head et al., 2020 [323]	Kazakhstan	Livestock owners/ farmers	914	CCHFV IgG/IgM ELISA (Vector-Best)	IgM-only: 3/914 (0.3%); IgG-only: 12/914 (1.3%); both: 2/914 (0.2%); any antibody: 17/914 (1.9%)	IgM-only: 0.1–1.0%; IgG-only: 0.8–2.3%; both: 0.1–0.8%; any antibody: 1.2–3.0%	NA	NA	NA	NA
Lozynskyi et al., 2020 [324]	Ukraine (Lviv Oblast)	Healthy adults	966	CCHFV IgG ELISA (Abbexa)	16/966 (1.7%)	1.0–2.7%	NR	—	NA	NA
Monsalve Arteaga et al., 2020 [325]	Spain (Castile-León)	Blood donors	516	CCHFV IgG/IgM ELISA (Vector-Best) + in-house ELISA + CCHFV IFA (Euroimmun)	Reactive in ≥1 assay: 6/516 (1.2%); confirmed ≥2 assays: 3/516 (0.6%)	Reactive ≥ 1 assay: 0.5–2.5%; confirmed ≥2 assays: 0.2–1.7%	NA	NA	NA	NA
Naidenova et al., 2020 [191]	Guinea	Rural residents	2207	CCHFV IgG ELISA (Vector-Best)	67/2207 (3.0%)	2.4–3.8%	NA	NA	NA	NA
Noreen et al., 2020 [326]	Pakistan (Islamabad)	Suspected cases	40	RT-qPCR	NA	NA	20/40 (50.0%)	35.2–64.8%	6/20 (30.0%)	14.5–51.9%
Nyataya et al., 2020 [327]	Kenya	Acute febrile patients	1033	CCHFV IgG/IgM ELISA (Vector-Best)	IgM: 4/1033 (0.4%); IgG/IgM: 0/1033 (0.0%); IgG: 20/1033 (1.9%)	IgM: 0.2–1.0%; IgG/IgM: 0.0–0.4%; IgG: 1.3–3.0%	NA	NA	NA	NA
Shahid et al., 2020 [328]	Pakistan (Punjab Province)	Adults	1052	CCHFV IgG ELISA (Vector-Best)	22/1052 (2.1%)	1.4–3.1%	NA	NA	NA	NA
Shahid et al., 2020 [329]	Pakistan (Chakwal District)	High-risk participants	Abattoir workers: 137; milkmen: 169; animal handlers: 147	CCHFV IgG ELISA (Vector-Best)	Overall: 26/453 (5.7%); animal handlers: 14/147 (9.5%); abattoir workers: 9/137 (6.6%); milkmen: 3/169 (1.8%)	Overall: 3.9–8.3%; animal handlers: 5.8–15.4%; abattoir workers: 3.5–12.0%; milkmen: 0.6–5.1%	NA	NA	NA	NA
Tripathi et al., 2020 [330]	India (Rajasthan)	Confirmed cases	4	CCHFV IgG/IgM ELISA + RT-qPCR	IgM ELISA: 2/4 (50.0%)	15.0–85.0%	3/4 (75.0%)	30.1–95.4%	3/4 (75.0%)	30.1–95.4%
Tumturk et al., 2020 [331]	Türkiye (Çorum)	Confirmed cases	399	CCHFV IgM ELISA + RT-PCR	IgM: 248/399 (62.2%)	57.3–66.8%	151/399 (37.8%)	33.2–42.7%	22/399 (5.5%)	3.7–8.2%
Umair et al., 2020 [332]	Pakistan	Suspected cases	280	RT-qPCR + partial S-segment sequencing	NA	NA	28/280 (10.0%)	7.0–14.1%	11/28 (39.3%)	23.6–57.6%
Yeşilbağ et al., 2020 [333]	Türkiye	Suspected cases	87	CCHFV IgM ELISA and/or RT-PCR	Laboratory-confirmed: 61/87 (70.1%) by IgM ELISA and/or RT-PCR; 95% CI: 59.8–78.7%				2/61 (3.3%)	0.9–11.2%
Zohaib et al., 2020 [136]	Pakistan	Participants	1872	CCHFV antibody ELISA (Vector-Best) + IFA	51/1872 (2.7%)	2.1–3.6%	NA	NA	NA	NA
Latasa et al., 2020 [334]	Spain	High-risk contacts of confirmed CCHF cases	49	CCHFV IgG by Vector Best immunoenzymatic assay and EuroImmun indirect immunofluorescence	0/49 (0.0%)	0.0–7.3%	NA	NA	NA	NA
Abdiyeva et al., 2019 [335]	Kazakhstan (Kyzylorda and Almaty oblasts)	Fever of unknown origin	802	CCHFV IgG/IgM ELISA (Vector-Best) + RT-qPCR + S/L-segment sequencing	IgG: 102/802 (12.7%); IgM: 4/802 (0.5%)	IgG: 10.6–15.2%; IgM: 0.2–1.3%	RT-qPCR: 3/802 (0.4%)	0.1–1.1%	NA	NA
Al-Abri et al., 2019 [336]	Oman	Symptomatic cases	88	CCHFV IgG/IgM ELISA (Vector-Best) + RT-qPCR (S segment) + CCHFV antigen ELISA (Vector-Best)	IgM: 4/88 (4.5%)	1.8–11.1%	RT-qPCR: 82/88 (93.2%)	RT-qPCR: 85.9–96.8%	32/88 (36.4%)	27.1–46.8%
Baumann et al., 2019 [337]	Mali	Malaria- negative paediatric febrile patients	489	RT-qPCR + S-segment sequencing	NA	NA	2/489 (0.4%)	0.1–1.5%	NA	NA
Bower et al., 2019 [338]	Sudan (Darfur)	Outbreak sera	65	CCHFV IgG/IgM ELISA (Vector-Best) + RT-qPCR + NGS	IgG: 11/65 (16.9%); IgM: 6/65 (9.2%)	IgG: 9.7–27.8%; IgM: 4.3–18.7%	7/65 (10.8%)	5.3–20.6%	1/5 (20.0%)	3.6–62.4%
Gozdas, 2019 [339]	Türkiye (Kastamonu)	Suspected cases	76	CCHFV IgM ELISA + PCR	IgM: 2/76 (2.6%)	0.7–9.1%	29/76 (38.2%)	28.1–49.4%	3/31 (9.7%)	3.3–24.9%
Hatami et al., 2019 [340]	Afghanistan (Kabul)	Suspected/ probable/ confirmed cases	120; sera tested: 108	CCHFV IgG/IgM ELISA	Overall: 29/108 (26.9%); IgM: 22/108 (20.4%); IgG: 7/108 (6.5%)	Overall: 19.4–35.9%; IgM: 13.9–28.9%; IgG: 3.2–12.8%	NA	NA	18/120 (15.0%)	9.7–22.5%
Mourya et al., 2019 [341]	India (Gujarat)	Human sera	4978	NIV in-house CCHFV IgG ELISA	25/4978 (0.5%)	0.3–0.7%	NA	NA	NA	NA
Niazi et al., 2019 [342]	Afghanistan (Herat Province)	Hospitalized cases	63	CCHFV IgM ELISA (Vector-Best) + RT-qPCR	IgM: 32/63 (50.8%)	38.8–62.7%	58/63 (92.1%)	82.7–96.6%	14/63 (22.2%)	13.7–33.9%
Rahden et al., 2019 [343]	Sudan (eastern and central regions)	Human sera	464	CCHFV IgG ELISA (Vector-Best) + Blackbox CCHFV IgG ELISA	12/464 (2.6%)	1.5–4.5%	NA	NA	NA	NA
Sahak et al., 2019 [344]	Afghanistan	Surveillance cases	891	CCHFV IgM ELISA + RT-PCR	Laboratory-confirmed: 293/891 (32.9%) by IgM ELISA and RT-PCR 95% CI: 29.9–36.0%				127/293 (43.3%)	37.8–49.1%
Sağmak Tartar et al., 2019 [345]	Türkiye	Confirmed cases	61	CCHFV IgM ELISA + RT-PCR	Laboratory-confirmed: 61 cases by CCHFV IgM ELISA and/or RT-PCR				1/61 (1.6%)	0.3–8.7%
Shahbazi et al., 2019 [346]	Iran (Kurdistan Province)	Participants	Butchers/slaughterhouse workers: 50; hunters/families: 50; healthcare workers: 50; clinical-laboratory attendees: 100	In-house CCHFV NP IgG ELISA (Pasteur Institute of Iran)	Overall: 1/250 (0.4%); butchers/slaughterhouse workers: 1/50 (2.0%); hunters: 0/50 (0.0%); healthcare workers: 0/50 (0.0%); clinical-laboratory attendees: 0/100 (0.0%)	Overall: 0.1–2.2%; butchers/slaughterhouse workers: 0.4–10.5%; hunters: 0.0–7.1%; healthcare workers: 0.0–7.1%; clinical-laboratory attendees: 0.0–3.7%	NA	NA	NA	NA
Yaqub et al., 2019 [347]	Pakistan (Chakwal District)	Suspected cases	3	CCHFV IgG ELISA (Vector-Best)	3 cases confirmed; IgG-positive	—	NA	NA	2/3 (66.7%)	20.8–93.9%;
Zhang et al., 2019 [348]	Sierra Leone	Patients with febrile jaundice	96	Metagenomic sequencing (Ion Torrent)	NA	NA	Sequencing reads: 1/96 (1.0%)	0.2–5.7%	NA	NA
Almasri et al., 2019 [349]	Saudi Arabia	Permanent abattoir workers during Hajj	80	PCR for CCHFV (Roche Diagnostics; LightCycler)	NA	NA	0/80 (0%)	0.0–4.6%	NA	NA
Metanat et al., 2018 [350]	Iran (Zahedan, Sistan and Baluchestan Province)	Family- associated cases	5	CCHFV ELISA + RT-PCR	5/5 (100%)	56.6–100.0%	5/5 (100%)	56.6–100.0%	0/5 (0.0%)	0.0–43.4%
Rehman et al., 2018 [351]	Pakistan (Karak District, Khyber Pakhtunkhwa)	Symptomatic cases	Cases: 6; contacts: 21	ELISA + RT-qPCR	NR	—	PCR: 2/6 (33.3%); high-risk contacts: 0/21 (0.0%)	Cases: 9.7–70.0%; contacts: 0.0–15.5%	Confirmed: 0/2 (0.0%); overall: 4/6 (66.7%)	Confirmed: 0.0–65.8%; overall: 30.0–90.3%
Sadeuh-Mba et al., 2018 [352]	Cameroon (East Region)	Pygmy participants	137	In-house CCHFV NP IgG ELISA	6/137 (4.4%)	2.0–9.2%	NA	NA	NA	NA
Shahhosseini et al., 2018 [353]	Iran (Mashhad, Razavi Khorasan Province)	Slaughterhouse workers	136	In-house CCHFV IgG sandwich ELISA	39/136 (28.7%)	21.7–36.8%	NA	NA	NA	NA
Temoçin et al., 2018 [354]	Türkiye (Yozgat)	Healthcare workers	Contact group: 67; non-contact group: 45	CCHFV IgG ELISA (Vector-Best) + IgM ELISA/PCR follow-up of reactive samples	Contact group: 2/67 (3.0%); non-contact group: 0/45 (0.0%)	Contact: 0.8–10.2%; non-contact: 0.0–7.9%	IgG positive underwent IgM/PCR; no evidence of acute infection reported	—	NA	NA
Vawda et al., 2018 [355]	South Africa (Free State and Northern Cape)	Healthy adults	387	CCHFV Mosaic 2 IgG/IgM IFA (Euroimmun)	IgG: 2/387 (0.5%); IgM: 0/2 (0.0%)	IgG: 0.1–1.9%; IgM: 0.0–65.8%	NA	NA	NA	NA
Voorhees et al., 2018 [203]	Mongolia	Nomadic herders and local residents	1926	In-house CCHFV IgG sandwich ELISA + PRNT	IgG ELISA: 27/1926 (1.4%); PRNT50: 8/12 (66.7%)	IgG ELISA: 1.0–2.0%; PRNT50: 39.1–86.2%	NA	NA	NA	NA
Ziauddin et al., 2018 [356]	Pakistan (Khyber Pakhtunkhwa)	Suspected cases	112	Antibody testing + RT-PCR + antigen testing	15/112 (13.4%) by CCHFV antigen testing and/or serology/RT-PCR 95% CI: 8.3–20.9%				5/15 (33.3%)	15.2–58.3%
Aslani et al., 2017 [357]	Iran	Pediatric suspected cases	908	In-house CCHFV IgM capture ELISA (MAC-ELISA) + one-step RT-PCR (Iran F2/R3)	IgM: 100/908 (11.0%)	9.1–13.2%	RT-PCR: 157/908 (17.3%)	RT-PCR: 15.0–19.9%	19/161 (11.8%)	7.7–17.7%
Bayram et al., 2017 [358]	Türkiye	Healthy participants	368	CCHFV IgG/IgM ELISA (Vector-Best) + RT-qPCR	IgG: 53/368 (14.4%); IgM: 2/53 (3.8%)	IgG: 11.2–18.4%; IgM: 1.0–12.8%	RT-qPCR: 0/2 (0.0%)	0.0–65.8%	NA	NA
Christova et al., 2017 [359]	Bulgaria	General population	1500	CCHFV IgG/IgM ELISA (Vector-Best) + CCHFV IFA (Euroimmun)	IgG: 55/1500 (3.7%); IgM: 0/55 (0.0%)	IgG: 2.8–4.7%; IgM subset: 0.0–6.5%	NA	NA	NA	NA
Khan et al., 2017 [360]	Pakistan	Confirmed cases	11	PCR (VetPCR™ CCHF Detection Kit, Bioin Gentech)	NA	NA	PCR: 11/11 (100%)	74.1–100.0%	4/11 (36.4%)	15.2–64.6%
Krasniqi et al., 2017 [361]	Albania	Suspected cases	128	CCHFV IgG/IgM ELISA + PCR	ELISA-reactive IgM/IgG: 27/128 (21.1%); IgM positive: 7/128 (5.5%)	ELISA-reactive: 14.9–29.0%; IgM: 2.7–10.9%	3/128 (2.3%)	0.8–6.7%	NR	—
Kuşcu et al., 2017 [362]	Türkiye	Index patients; healthcare workers	Index patients: 2; healthcare workers: 20	CCHFV IgG/IgM ELISA (Vector-Best) + RT-qPCR + S-segment sequencing	Exposed HCWs: 0/20 (0.0%)	0.0–16.1%	Index patients—RT-qPCR: 2/2 (100%)	34.2–100.0%	Index patients: 2/2 (100%)	34.2–100.0%
Mostafavi et al., 2017 [363]	Iran	Butchers; slaughterhouse workers	190	CCHFV IgG ELISA	IgG: 31/190 (16.3%)	11.7–22.2%	NA	NA	NA	NA
Mourya et al., 2017 [364]	India	Suspected cases	69	CCHFV IgM ELISA + RT-qPCR	21/69 (30.4%) by IgM ELISA and/or real-time RT-PCR 20.8–42.1%				7/21 (33.3%)	17.2–54.6%
Muianga et al., 2017 [365]	Mozambique	Febrile patients	300	CCHFV IgG ELISA (Vector-Best)	IgG: 8/300 (2.7%)	1.4–5.2%	NA	NA	NA	NA
Salehi-Vaziri et al., 2017 [366]	Iran	Outbreak cases	3	CCHFV IgM capture ELISA + RT-PCR + sequencing	IgM: 3/3 (100.0%)	43.8–100.0%	3/3 (100.0%)	43.8–100.0%	0/3 (0.0%)	0.0–56.2%
Shahhosseini et al., 2017 [367]	Iran	Three hospitalized cases from northern Iran, June–July 2015	3	In-house CCHFV IgG/IgM ELISA + one-step RT-PCR (S segment) + Sanger sequencing	IgM: 2/3 (66.7%); IgG: 0/3 (0.0%)	IgM: 20.8–93.9%; IgG: 0.0–56.2%	3/3 (100%)	43.8–100.0%	1/3 (33.3%)	6.1–79.2%
Palomar et al., 2017 [238]	Spain	Human participants in Iberian Peninsula surveillance	228	Crimean–Congo Fever Virus Mosaic 2 indirect IFA (Euroimmun AG, Lübeck, Germany)	0/228 (0.0%)	0.0–1.7%	NA	NA	NA	NA
Akuffo et al., 2016 [219]	Ghana	Abattoir workers	Baseline: 108; serial sera: 188	CCHFV IgG/IgM ELISA (Vector-Best)	Baseline IgG: 6/108 (5.6%); serial IgG: 7/188 (3.7%); serial IgM: 1/188 (0.5%)	Baseline IgG: 2.6–11.6%; serial IgG: 1.8–7.5%; serial IgM: 0.1–3.0%	NA	NA	NA	NA
Bukbuk et al., 2016 [368]	Nigeria	Serology cohort; febrile cohort	Serology: 1189; molecular: 380	In-house recombinant CCHFV nucleoprotein IgG/IgM ELISA + RT-PCR + NGS	IgG: 126/1189 (10.6%); IgM: 42/1189 (3.5%); IgG + IgM: 7/1189 (0.6%)	IgG: 9.0–12.5%; IgM: 2.6–4.7%; both: 0.3–1.2%	1/380 (0.3%)	0.0–1.5%	NA	NA
Cikman et al., 2016 [369]	Türkiye	Community residents	372	CCHFV IgG/IgM ELISA (Vector-Best) + RT-qPCR	IgG: 52/372 (14.0%); IgG + IgM: 7/372 (1.9%)	IgG: 10.8–17.9%; IgG + IgM: 0.9–3.8%	RT-qPCR: 1/7 (14.3%)	2.6–51.3%	NA	NA
Gazi et al., 2016 [370]	Türkiye	Rural residents	324	CCHFV Mosaic 2 IgG IFA (Euroimmun)	IgG: 12/324 (3.7%)	2.1–6.4%	NA	NA	NA	NA
Greiner et al., 2016 [371]	Georgia	Rural residents	435	CCHFV IgG/IgM ELISA (Vector-Best) + RT-PCR for the IgM-reactive participant	IgG: 12/435 (2.8%); initial IgM: 1/435 (0.2%)	IgG: 1.6–4.8%; initial IgM: 0.04–1.3%	0/1 (0.0%)	0.0–79.3%	NA	NA
Kilinc et al., 2016 [372]	Türkiye	Suspected cases	281	CCHFV IgG/IgM ELISA + RT-qPCR	102/281 (36.3%) by serology and PCR 95% CI: 30.9–42.1%				4/102 (3.9%)	1.5–9.7%
Kuchuloria et al., 2016 [373]	Georgia	Febrile patients	537	CCHFV IgM ELISA	IgM: 3/537 (0.6%)	0.2–1.6%	NA	NA	NA	NA
Leblebicioglu et al., 2016 [374]	Türkiye	Healthcare exposures	51	CCHFV IgG/IgM ELISA + PCR	25/51 (49.0%) by IgM/IgG ELISA and/or PCR 95% CI: 35.9–62.3%				4/25 (16.0%)	6.4–34.7%
O’Hearn et al., 2016 [375]	Sierra Leone	Suspected-Lassa sera	Surveyed: 675; valid results: 641	MAGPIX CCHFV NP IgG assay	IgG: 13/641 (2.0%)	1.2–3.4%	NA	NA	NA	NA
Rathod et al., 2016 [376]	India	Confirmed cases	10	RT-PCR	NA	NA	All 10 cases RT-PCR positive	NA	3/10 (30.0%)	10.8–60.3%
Sharififard et al., 2016 [377]	Iran	Probable cases	42	Serology (assay NR)	17/42 (40.5%)	27.0–55.5%	NA	NA	Confirmed: 6/17 (35.3%); probable: 12/42 (28.6%)	Confirmed: 17.3–58.7%; probable cohort: 17.2–43.6%
Singh et al., 2016 [378]	India	Positive serum samples	3	Nested RT-PCR (N gene) + Sanger sequencing	NA	NA	3/3 (100%)	43.8–100.0%	NR	—
Todd et al., 2016 [379]	Afghanistan	Military recruits	809	CCHFV antibody ELISA (Vector-Best)	33/809 (4.1%)	2.9–5.7%	NA	NA	NA	NA
Wasfi et al., 2016 [380]	Tunisia	Febrile patients; slaughterhouse workers	Febrile patients: 181; slaughterhouse workers: 38	CCHFV IgG/IgM ELISA (Vector-Best) + RT-qPCR (S segment)	Febrile patients: IgM 5/181 (2.8%); slaughterhouse workers: IgG 2/38 (5.3%)	Febrile IgM: 1.2–6.3%; slaughterhouse IgG: 1.5–17.3%	Human sera: 0/219 (0.0%)	0.0–1.7%	NA	NA
Yadav et al., 2016 [381]	India	Symptomatic nurses; additional nurses; community contacts	Symptomatic nurses: 4; additional nurses: 5; community contacts: 37	CCHFV IgM ELISA (Vector-Best) + RT-qPCR + RT-PCR + sequencing	Symptomatic nurses—IgM: 4/4 (100%); contacts—IgM: 1/37 (2.7%); additional nurses: 0/5 (0.0%)	Symptomatic nurses: 51.0–100.0%; contacts: 0.5–13.8%; additional nurses: 0.0–43.4%	Symptomatic nurses: 4/4 (100%); contacts: 1/37 (2.7%)	Symptomatic nurses: 51.0–100.0%; contacts: 0.5–13.8%	2/4 (50.0%)	15.0–85.0%
Yildirmak et al., 2016 [382]	Türkiye	Cluster cases	5	RT-PCR	NA	NA	All 5 cases positive	—	1/5 (20.0%);	3.6–62.4%;
Ayatollahi et al., 2015 [383]	Iran	Confirmed Cases	9	CCHFV IgG/IgM ELISA + PCR	PCR and IgM/IgG ELISA reported positive in patient samples				0/9 (0.0%)	0.0–29.9%
Erenler et al., 2015 [384]	Türkiye	Suspected cases	240	RT-PCR	NA	NA	97/240 (40.4%)	34.4–46.7%	21/97 (21.6%)	14.6–30.8%
Karlberg et al., 2015 [385]	Iran	Suspected cases	17	CCHFV IgG/IgM ELISA (Vector-Best) + in-house IgG/IgM IFA + microneutralization + RT-qPCR (S segment) + endpoint RT-PCR	IgM: 6/17 (35.3%) by ELISA and/or in-house IFA	17.3–58.7%	12/17 (70.6%)	46.9–86.7%	0/12 (0.0%)	0.0–24.3%
Khurshid et al., 2015 [386]	Afghanistan; Pakistan	Suspected cases	100	BDSL CCHFV IgM ELISA + nested RT-PCR (S segment) + sequencing	IgM: 35/100 (35.0%)	26.4–44.7%	39/100 (39.0%)	30.0–48.8%	10/49 (20.4%)	11.5–33.6%
Lani et al., 2015 [387]	Malaysia	Orang Asli participants	682	CCHFV IgG ELISA (Vector-Best)	IgG: 1/682 (0.1%)	0.0–0.8%	NA	NA	NA	NA
Tigoi et al., 2015 [388]	Kenya	Febrile patients	379	CCHFV IgG/IgM ELISA (Vector-Best)	IgG: 97/379 (25.6%); IgM: 0/379 (0.0%)	IgG: 21.5–30.2%; IgM: 0.0–1.0%	NA	NA	NA	NA
Mohd Shukri et al., 2015 [389]	Malaysia	Farm workers	85	VectoCrimean-CHF-IgG ELISA (Vector-Best, Novosibirsk, Russia)	0/85 (0.0%)	0.0–4.3%	NA	NA	NA	NA

Note: Results are shown as positive/tested (%) when raw counts were available. Source-reported percentages are retained when the corresponding positive/tested counts cannot be recovered without assumption. 95% CIs are Wilson score intervals unless otherwise stated. NA = not applicable; NR = not separately reported; — = not calculable.

## Data Availability

Supplementary Data S1 (XLSX) provides reconstructed search strategies, eligibility/coding rules, study-level datasets, study-family relationships, quality/context fields and descriptive summaries. Supplementary File S2 (PY) contains reproducibility checks for report and stream reconciliation, descriptive summaries, sensitivity analyses and confidence intervals.

## Supplementary Materials

The following supporting information can be downloaded at https://www.mdpi.com/article/10.3390/ani16203221/s1, Supplementary Data S1 (XLSX): Database search strategies, eligibility rules, animal, tick and human datasets, linked/nonadditive report relationships, descriptive summaries and structured quality/context fields. Supplementary File S2 (PY): Reproducibility checks for report reconciliation, stream counts, descriptive summaries, sensitivity analyses and Wilson 95% confidence intervals. Linked/nonadditive publications recorded in Supplementary Data S1 are cited here for reference completeness [397,398,399,400,401,402,403,404,405,406,407].
